# Proteomic profiling of serum identifies a molecular signature that correlates with clinical outcomes in COPD

**DOI:** 10.1371/journal.pone.0277357

**Published:** 2022-12-08

**Authors:** Rania Dagher, Paul Fogel, Jingya Wang, David Soussan, Chia-Chien Chiang, Jennifer Kearley, Daniel Muthas, Camille Taillé, Patrick Berger, Arnaud Bourdin, Cécile Chenivesse, Sylvie Leroy, Gary Anderson, Alison A. Humbles, Michel Aubier, Roland Kolbeck, Marina Pretolani, on behalf of the COBRA Consortium

**Affiliations:** 1 Bioscience COPD/IPF, Research and Early Development, Respiratory & Immunology, BioPharmaceuticals R&D, AstraZeneca, Gaithersburg, Maryland, United States of America; 2 Independent Consultant, Paris, France; 3 Translational Science and Experimental Medicine, Research and Early Development, Respiratory & Immunology, BioPharmaceuticals R&D, AstraZeneca, Gaithersburg, Maryland, United States of America; 4 Inserm UMR1152, Physiopathologie et Epidémiologie des Maladies Respiratoires, Université Paris Cité, Faculté de Médecine, Site Bichat, Paris, France; 5 Laboratory of Excellence INFLAMEX, Université Paris-Cité, Paris, France; 6 Data Sciences and AI, BioPharmaceuticals R&D, AstraZeneca, Gaithersburg, Maryland, United States of America; 7 Translational Science and Experimental Medicine, Research and Early Development, Respiratory & Immunology, BioPharmaceuticals R&D, AstraZeneca, Gothenburg, Sweden; 8 Service de Pneumologie A - Groupement Hospitalier Universitaire Nord Bichat-Claude Bernard, Paris, France; 9 Inserm UMR1045, Université de Bordeaux, Service d’explorations Fonctionnelles Respiratoires, Centre Hospitalo-Universitaire de Bordeaux, Bordeaux, France; 10 Inserm UMR1046, Université de Montpellier, Département de Pneumologie et Addictologie, Centre Hospitalo-Universitaire de Montpellier, Montpellier, France; 11 Inserm UMR1158, Université Pierre et Marie Curie, Service de Pneumologie et Réanimation médicale, Centre Hospitalo-Universitaire La Pitié Salpêtrière, Paris, France; 12 Université de Nice and Service de Pneumologie Hôpital Pasteur, Centre Hospitalo-Universitaire de Nice, Nice, France; 13 Lung Health Research Centre, Department of Pharmacology and Therapeutics, University of Melbourne, Melbourne, Victoria, Australia; Imperial College London, UNITED KINGDOM

## Abstract

**Objective:**

Novel biomarkers related to main clinical hallmarks of Chronic obstructive pulmonary disease (COPD), a heterogeneous disorder with pulmonary and extra-pulmonary manifestations, were investigated by profiling the serum levels of 1305 proteins using Slow Off-rate Modified Aptamers (SOMA)scan technology.

**Methods:**

Serum samples were collected from 241 COPD subjects in the multicenter French Cohort of Bronchial obstruction and Asthma to measure the expression of 1305 proteins using SOMAscan proteomic platform. Clustering of the proteomics was applied to identify disease subtypes and their functional annotation and association with key clinical parameters were examined. Cluster findings were revalidated during a follow-up visit, and compared to those obtained in a group of 47 COPD patients included in the Melbourne Longitudinal COPD Cohort.

**Results:**

Unsupervised clustering identified two clusters within COPD subjects at inclusion. Cluster 1 showed elevated levels of factors contributing to tissue injury, whereas Cluster 2 had higher expression of proteins associated with enhanced immunity and host defense, cell fate, remodeling and repair and altered metabolism/mitochondrial functions. Patients in Cluster 2 had a lower incidence of exacerbations, unscheduled medical visits and prevalence of emphysema and diabetes. These protein expression patterns were conserved during a follow-up second visit, and substanciated, by a large part, in a limited series of COPD patients. Further analyses identified a signature of 15 proteins that accurately differentiated the two COPD clusters at the 2 visits.

**Conclusions:**

This study provides insights into COPD heterogeneity and suggests that overexpression of factors involved in lung immunity/host defense, cell fate/repair/ remodelling and mitochondrial/metabolic activities contribute to better clinical outcomes. Hence, high throughput proteomic assay offers a powerful tool for identifying COPD endotypes and facilitating targeted therapies.

## Introduction

Chronic obstructive pulmonary disease (COPD) is a difficult to treat disease, characterized by irreversible airflow obstruction and often associated with lung emphysema. These events result from persistent lung inflammation and tissue remodeling that leads to respiratory insufficiency and functional disability [1]. Cigarette smoke, but also genetic/epigenetic alterations leading to lung accelerated aging, have been shown to predispose individuals to COPD [2–4]. However, whether markers of these processes are detected in peripheral blood of patients and relate to clinical traits of the disease remains elusive.

COPD manifests in different clinical phenotypes according to the degree of airflow obstruction, frequency of acute exacerbations, emphysema and airway inflammation [5]. In addition, pulmonary and cardio-metabolic comorbidities may impact COPD prognosis and therapeutic management [6]. Although numerous studies have addressed analytical approaches for identifying novel COPD endotypes underlying these clinical phenotypes [7–14], those approaches did not include the measurement of biomarkers of lung injury/repair and of pulmonary, or extra-pulmonary co-morbidities, these factors being major contributors of COPD onset and progression [6].

Given the heterogeneity in COPD pathophysiology and clinical presentation, robust and wide analytic methodologies are required to characterize novel endotypes. To this end, High-throughput proteomic technology, Slow Off-rate Modified Aptamers (SOMA)scan, has been developed for quantitatively assessing hundreds of proteins specifically in serum and plasma samples with high sensitivity and specificity [15, 16]. This platform has previously proved useful in being able to measure simultaneously large numbers of proteins in different organ diseases, including lung [17, 18].

The current study was aimed at identifying novel biomarkers related to main clinical hallmarks of COPD, to its most frequent co-morbidities and to medication. This was performed by profiling the serum levels of 1305 proteins by SOMAscan in 241 patients included in the multicenter prospective French Cohort of Bronchial Obstruction and Asthma (COBRA) [19, 20]. Unsupervised clustering of differentially expressed proteins combined to gene ontology (GO) pathway analysis, classified COPD patients into clinical clusters. Findings were validated in another independent series of COPD patients enrolled in the Melbourne Longitudinal COPD Cohort (MLCC) [21]. Further analyses were carried out for determining a short protein fingerprint linked to GO biological processes, as a useful tool to discriminate patient clusters, for monitoring its stability at a follow-up and, ultimately, for identifying potential novel therapeutic targets to adapt clinical managements.

## Methods

### Study populations

Stable COPD patients (n = 241) were included in the COBRA cohort [19, 20] (CPP Ile-de-France I Ethics Committee, n° 09–11962) and written informed consent was obtained before inclusion (S1 Table in S1 File). Serum aliquots were collected for the measurement of the levels of hemoglobin and of C reactive protein (CRP) and for SOMAscan analyses. The evolution of the clinical outcomes and of proteomic profiles was assessed in 163 COPD patients out of the 241 having a follow up visit 7.5 ± 6.6 months (mean ± SD) after inclusion (S1 Table in S1 File). SOMAscan data were validated in a separate series of 47 COPD patients originating from the MLCC [21] (S2 Table in S1 File). Serum samples from n = 50 control healthy subjects were used for comparisons.

### SOMAscan analysis

A total of 1305 analytes were quantified in patient serum using the SOMAscan high throughput proteomic assay (SomaLogic, Boulder, CO, USA) at National Jewish Health (Denver, Colorado, United States of America) [22]. The raw SOMAscan data were standardized by four steps: hybridization normalization, place scaling, median signal normalization and calibration, according to manufacturer’s instructions (http://somalogic.com/wp-content/uploads/2017/06/SSM-071-Rev-0-Technical-Note-SOMAscan-Data-Standardization.pdf). The normalized expression values were then log2 transformed for downstream analyses. Proteins were described in the manuscript using SOMAscan target names.

### Data processing and bioinformatical approaches

**Fig 1:**
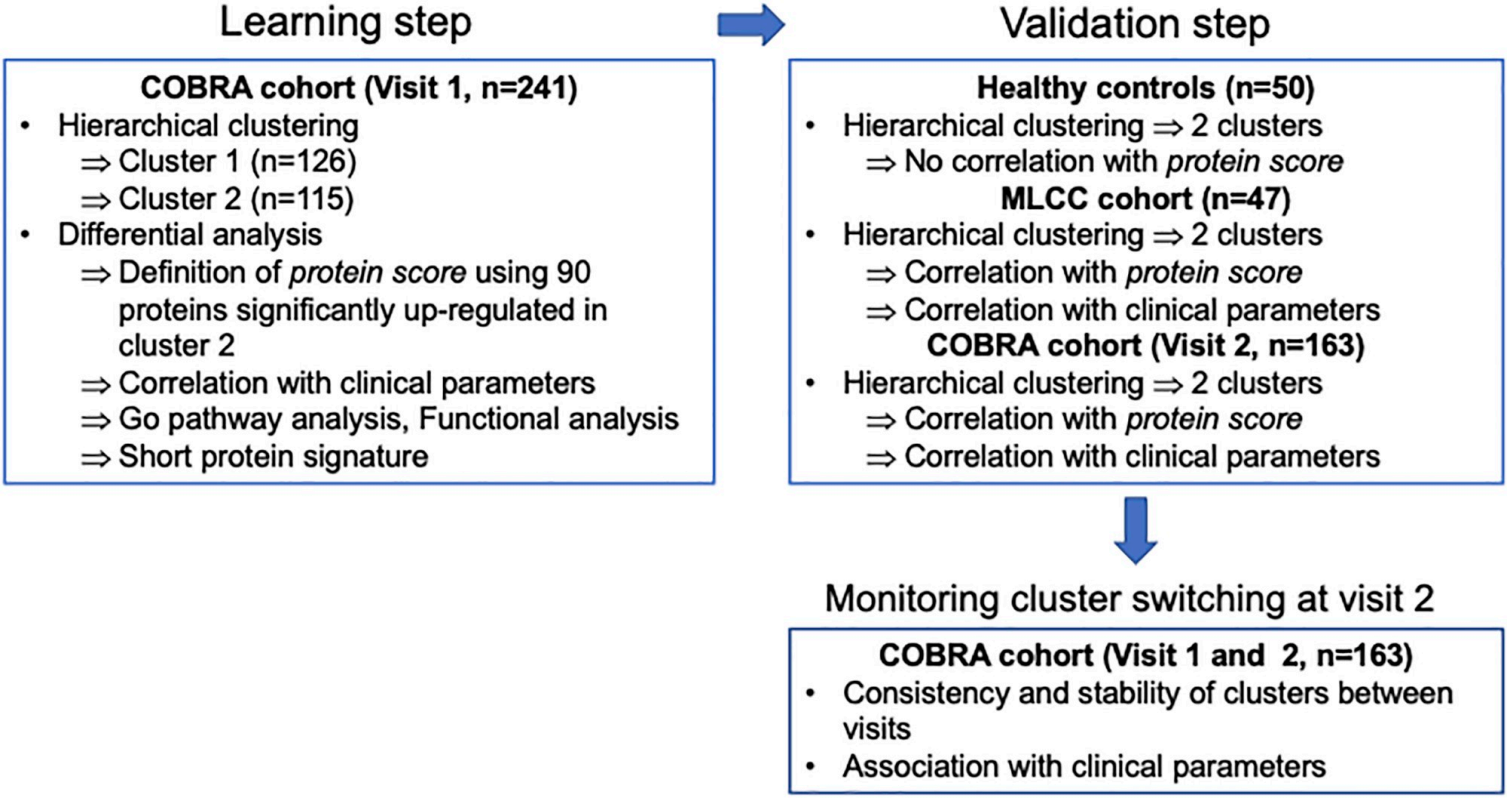
Study flow diagram. Stable COPD patients (n = 241) were included in the COBRA cohort at visit 1 and serum SOMAscan analyses were performed. Two COPD clusters, Cluster 1 (n = 126) and Cluster 2 (n = 115) with distinct protein expression patterns were identified through unsupervised hierarchical clustering. The differentially expressed proteins in Cluster 2 were submitted to GO pathways analysis for further functional enrichment study, then median expression values from 90 proteins up-regulated in Cluster 2 (defined as “protein scores”) were generated for each COPD cluster. Association studies were performed between COPD clusters or corresponding protein and clinical parameters. The evolution of the clinical outcomes and of proteomic profiles was assessed in 163 COPD patients out of the 241 having a follow up visit (visit 2) after inclusion (visit 1). Expression patterns associated with Cluster 1 (n = 97) and Cluster 2 (n = 66) were verified in COBRA cohort at visit 2 on the basis of protein scores previously defined at visit 1. Protein signatures representative of each COPD cluster were identified to monitor clinical patient stability at visit 2. SOMAscan data obtained in the COBRA cohort were validated in a separate group of 47 COPD patients originating from the MLCC cohort. Serum samples from n = 50 control healthy subjects were included for comparisons.

The bioinformatics approaches are summarized in Fig 1 and follow a typical machine learning approach with a learning step and a validation step. For each cohort consisting exclusively of either diseased or healthy patients, hierarchical clustering was performed using R, based on the top 10% proteins by expression variation, to first segment the population. Differential expression analyses between the detected clusters were performed using the Limma package [23]. Proteins with fold change >1.5 and false discovery rate (FDR) <0.05 were defined as significant. Functional analyses of the differentially expressed proteins were performed using the GO pathway database.

During the learning step, hierarchical clustering was performed for COPD patients from the COBRA cohort at the time of inclusion (n = 241). Two COPD clusters: Cluster 1 (n = 126) and Cluster 2 (n = 115) with different protein expression patterns were identified. Using the 10% of proteins that differed most between the two clusters, the protein score for each patient was defined as the median expression of 90 proteins that were upregulated in Cluster 2. In addition, a short signature consisting of 15 proteins WAS associated with each cluster. Cluster 2-associated biomarkers were identified by sorting 10% of the most differentially expressed proteins representative of highly enriched pathways from GO pathway analysis and exhibiting the highest odd-ratios (OD) within their corresponding pathways; Cluster-1 associated biomarkers included top four of the 6 up-regulated biomarkers after exclusion of C3b that failed to show any differential expression in the MLCC cohort. Association studies between COPD clusters or corresponding proteins and clinical parameters were performed.

During the validation step, the reproducibility of the clustering patterns found in the learning step was assessed in 163 COPD patients of the 241 patients from the COBRA cohort who had a follow-up visit (visit 2) after inclusion, in 47 COPD patients from the MLCC cohort, and in n = 50 healthy subjects, included in the analysis as negative controls. Two main clusters were identified for each data set by hierarchical clustering. Importantly, clustering was based on all proteins (*i*.*e*., significant proteins found at visit 1 in the COBRA cohort were not considered) to ensure that our validation approach was agnostic to the results found at visit 1. We then tested whether clusters found in subsequent clusters were significantly different on the basis of the protein score defined in the learning step. In addition, we identified differentially expressed proteins and evaluated their enrichment with respect to significant proteins found in the learning step.

Finally, the short protein signature identified during the learning step was used to monitor the evolution of patients from the COBRA cohort between visit 1 and visit 2.

Data processing and statistical analyses are described in details in the S1 File.

## Results

### Subject analyzed

GOLD stage distribution, smoking habits, respiratory function, and medication were comparable in COPD patients included in the COBRA cohort, when comparing visit 1 and visit 2 (S1 Table in S1 File). In contrast, a significant reduction in the incidence of patients having exacerbations (p = 0.003) was observed at visit 2, as compared to visit 1 (S1 Table in S1 File). The remaining 78 COPD patients having only visit 1 had less severe disease than the 163 having 2 visits. This was attested by their higher distribution into GOLD 1 group, values of % predicted post-bronchodilator forced expiratory volume in one second (FEV_1_) and FEV_1_/forced vital capacity (FVC), transfer factor of the lung for carbon monoxide (DLCO), unscheduled medical visits, oral corticosteroid (OCS) use, but similar onset and number of exaberbations and comorbidities (S1 Table in S1 File).

The 47 COPD patients from the MLCC cohort were more severe and symptomatic than those included in the COBRA cohort, in terms of GOLD stage, airflow obstruction, incidence of cough, treatments with muscarinic antagonists, long-term inhaled steroids (ICS) and oxygen therapy (S1 and S2 Tables in S1 File).

### Identification of two COPD subtypes in association with distinct biological hallmarks

**Fig 2:**
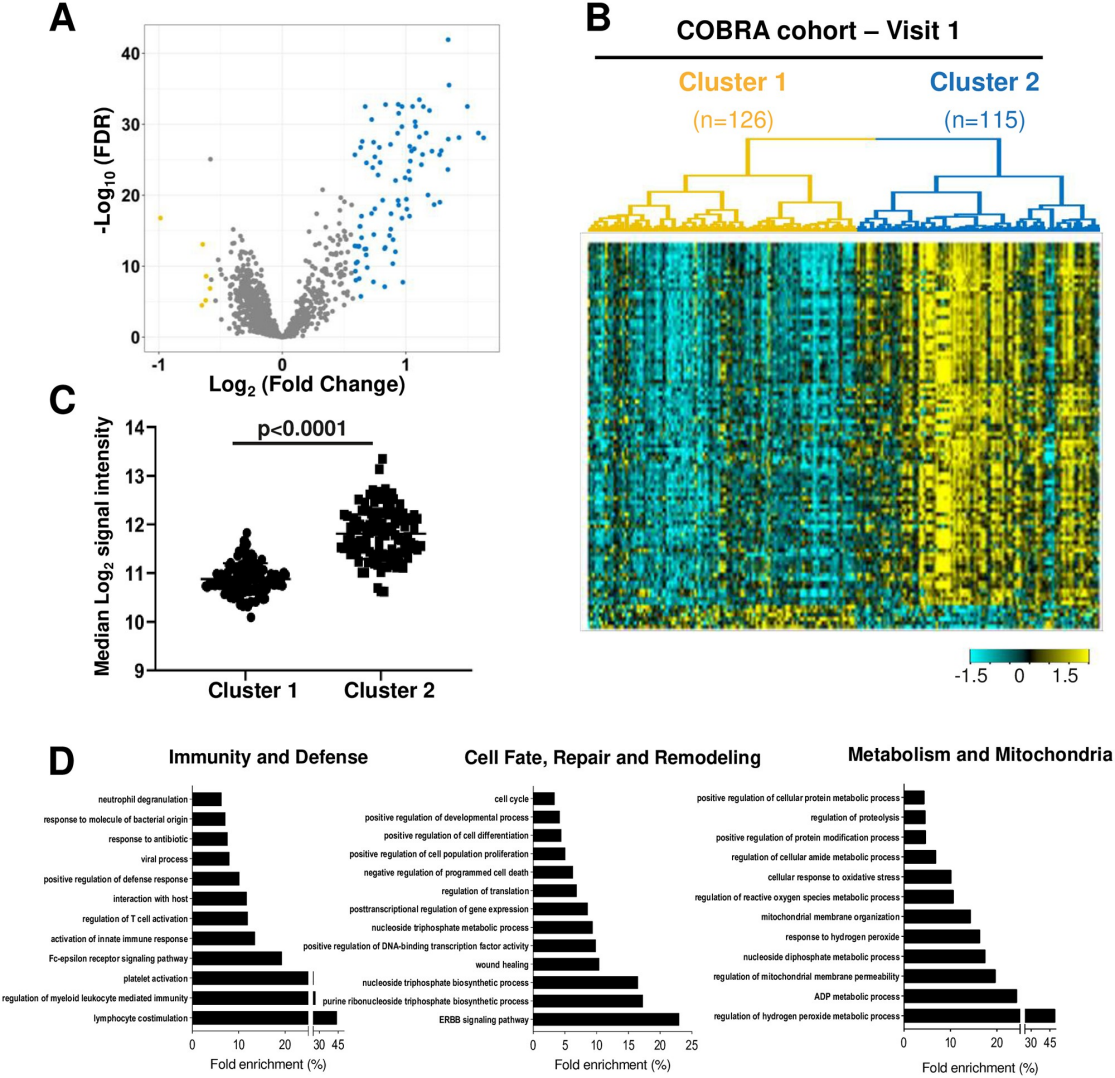
Two COPD patient Clusters show differentially expressed proteins enriched for distinct biological pathways. (A) Volcano plot showing differentially expressed proteins between Cluster 1 and Cluster 2. X axis corresponds to log2 (fold change) and Y axis corresponds to -log10 (FDR). Yellow indicates proteins with significant downregulation and blue indicates those with significant upregulation (Fold change >1.5 and FDR <0.05 for both comparisons); (B) Heatmap showing the expression pattern of differentially expressed proteins between the two clusters. (C) Median expression of 90 proteins associated with Cluster 2; (D) Top GO pathways comparing Cluster 2 *versus* Cluster 1, regrouped in three core functions defined as “Immunity and defense”, “Cell fate, repair and remodeling”, and “Metabolism and mitochondria”.

Hierarchical clustering, with the top 10% proteins by expression variation, revealed two COPD subtypes (126 and 115 patients in Clusters 1 and 2, respectively) within the 241 patients from the COBRA cohort at visit 1. A total of 96 proteins were differentially expressed between the two clusters (fold change >1.5 and FDR<0.05). Of these, 6 and 90 proteins were significantly up-regulated, in Clusters 1 and 2 respectively (Fig 2A and 2B, Table 1 and S3 Table in S1 File). Evaluation of the protein score (median expression) of the 90 proteins associated with Cluster 2, confirmed this significantly elevated levels (Fig 2C).

**Table 1:** Biological classification of the top 90 proteins enriched in Cluster 2 COPD patients.

Biological processes (originating from the GO database)	Proteins
• ERBB signaling pathway	**AREG**, GRB2 adapter protein, SRCN1, PDPK1, PKC-A, **SHC1**, MK01
• Wound healing	PKC-B-II, VAV, SRCN1, LYN, SMAD2, LYNB, Caspase-3, PDPK1, RAC1, Haemoglobin, METAP1, PRKACA, annexin I, PKC-A, FYN, 14-3-3 protein ζ/δ, PTP-1C, GPVI, ERK-1, NCC27, MK01
• Posttranscriptional regulation of gene expression	**eIF-4H**, **14-3-3 protein β/α**, 14-3-3 protein ζ/δ, GAPDH, PKC-A, RPS6KA3, hnRNP A2/B1, ERK-1, MK01, eIF-5A-1
• Negative regulation of programmed cell death	SRCN1, NDP kinase B, Caspase-3, PDPK1, CK2-A1:B, Sphingosine kinase 1, α-Synuclein, annexin I, PKC-A, RPS6KA3, UFM1, FYN, 14-3-3 protein ζ/δ, PPID, TCTP, PA2G4, **SHC1**, BAD, eIF-5A-1, **CD40 ligand**, Ubiquitin+1, Lactoferrin, Azurocidin, STAT3
• Positive regulation of cell proliferation	**AREG**, **FGF16**, **SHC1**, CK2-A1:B, PTP-1C, BAD, MK01, eIF5A-1, STAT3
• Positive regulation of cell differentiation	**BTK**, LYN, SMAD2, LYNB, NDP kinase B, RAC1, annexin I, PKC-A, RPS6KA3, FYN, PA2G4, BAD, CPNE1, NCC27, eIF-5A-1, Lactoferrin
• Positive regulation of developmental process	**BTK**, PKC-B-II, SRCN1, LYN, SMAD2, LYNB, NDP kinase B, PDPK1, RAC1, Sphingosine kinase 1, annexin I, PKC-A, RPS6KA3, FYN, HXK2, PA2G4, BAD, CPNE1, NCC27, eIF5A-1, Lactoferrin, STAT3
• Cell cycle	FER, PKC-B-II, IMB1, SRCN1, NSF1C, SBDS, CK2-A1:B, IF4G2, PRKACA, PKC-A, RPS6KA3, RAN, 41, PTP-1C, PA2G4, UBC9, ERK-1, MK01
• Regulation of myeloid leukocyte mediated immunity	**BTK**, FER, LYN, LYNB, PDPK1, ARGI1, Sorting nexin 4
• Fce receptor signaling pathway	**BTK**, FER, VAV, GRB2 adapter protein, LYN, LYNB, PDPK1, RAC1, **SHC1**, ERK-1, MK01
• Activation of innate immune response	**BTK**, SP-D, SRCN1, LYN, LYNB, PDPK1, UBE2N, PRKACA, RPS6KA3, FYN, Ubiquitin+1
• Regulation of T cell activation	**CD40 ligand**, CSK, SRCN1, Caspase-3, PDPK1, PTP-1C, DUSP3
• Positive regulation of defense response	**BTK**, SP-D, VAV, SRCN1, LYN, LYNB, PDPK1, UBE2N, α-Synuclein, PRKACA, RPS6KA3, FYN, ARGI1, ERK-1, Ubiquitin+1, Lactoferrin, Sorting nexin 4
• Neutrophil degranulation	IMB1, NDP kinase B, CK2-A1:B, RAC1, Haemoglobin, IMDH1, PTP-1C, PA2G4, ARGI1, Cyclophilin A, CPNE1, BPI, MK01, Lactoferrin, Azurocidin
• ADP metabolic process	GAPDH, Myokinase, HXK2, Triosephosphate isomerase, BAD
• Mitochondrial membrane organisation and permeability	**Cyclophilin F**, α-Synuclein, 14-3-3 protein ζ/δ, HXK2, BAD, STAT3, **14-3-3 protein** β/α
• Cellular response to oxidative stress	**Carbonic anhydrase XIII**, FER, SRCN1, NDP kinase B, α-Synuclein, annexin I, ARGI1, ERK-1, MK01
• Positive regulation of protein modification process	**AREG**, CSK, SRCN1, LYN, LYNB, PDPK1, CK2-A1:B, RAC1, Sphingosine kinase 1, UBE2N, α-Synuclein, PRKACA, PKC-A, FYN, UBC9, EDAR, **SHC1**, ERK-1, MK01, **CD40 ligand**, Ubiquitin+1, Lactoferrin, Azurocidin, STAT3, Ubiquitin+1
• Cellular response to environmental stimulus	**H2A3**, GRB2 adapter protein
• Actin filament organisation	**Tropomyosin 4**, GRB2 adapter protein, FER

41: Protein 4.1; AREG: Amphiregulin; ARGI1: Arginase-1; BAD: Bcl2-associated agonist of cell death; BPI: Bactericidal permeability-increasing protein; BTK: Tyrosine-protein kinase BTK; CK2A1:B: Casein kinase II 2-alpha:2-beta haeterotetramer; CPNE1: Copine1; CSK: Tyrosine-protein kinase CSK; PPID: Peptidyl-prolyl cis-trans isomerase D or Cyclophilin D; Cyclophilin F; DUS3: Dual specificity protein phosphatase 3; EDAR: Tumor necrosis factor receptor superfamily member EDAR; eIF-4H: Eukaryotic translation initiation factor 4H; eIF-5A-1: Eukaryotic translation initiation factor 5A-1; ERK-1: Mitogen-activated protein kinase 3 (MAPK3); FER: Tyrosine-protein kinase Fer; FGF-16: Fibroblast growth factor 16; FYN: Tyrosine-protein kinase Fyn; GAPDH: Glyceraldehyde-3-phosphate dehydrogenase; GPVI: Platelet glycoprotein VI; GRB2 adapter protein: Growth factor receptor-bound protein 2; hnRNP A2/B1: Heterogeneous nuclear ribonucleoproteins A2/B1; HXK2: Hexokinase-2; IF4G2: Eukaryotic translation initiation factor 4 gamma 2; IMB1: Importin subunit β-1; IMDH1: Inosine-5’-monophosphate dehydrogenase 1; LYN: Tyrosine-protein kinase Lyn; LYNB: Tyrosine-protein kinase Lyn, isoform B; MK01: Mitogen-activated protein kinase 1; M2-PK: Pyruvate kinase PKM; METAP1: Methionine aminopeptidase 1; NCC27 or CLIC1: Chloride intracellular channel protein 1; NDP kinase B: Nucleoside diphosphate kinase B; NSF1C: NSFL1 cofactor p47; PA2G4: Proliferation-associated protein 2G4; PDPK1: 3-phosphoinositide-dependent protein kinase 1; PRKACA: cAMP-dependent protein kinase catalytic subunit α; PKC-A: Protein kinase C, α type; PKC-B-II: Protein kinase C, β type (splice variant β-II); PTP-1C or PTPN6: Tyrosine-protein phosphatase non-receptor type 6; RAC1: Ras-related C3 botulinum toxin substrate 1; RAN: GTP-binding nuclear protein Ran; RPS6KA3: Ribosomal protein S6 kinase α3; SBDS: Ribosome maturation protein SBDS; SHC1: SHC-transforming protein 1; SMAD2: Mothers against decapentaplegic homolog 2; SP-D: Pulmonary surfactant associated protein D; SRCN1 or SRC: Proto-oncogene tyrosine-protein kinase Src; STAT3: Signal transducer and activator of transcription 3; SUMO3: Small ubiquitin-related modifier 3; TCTP: Translationally-controlled tumour protein; UBC9: SUMO-conjugating enzyme UBC9; UBE2N: Ubiquitin-conjugating enzyme E2 N; UFM1: Ubiquitin-fold modifier 1; VAV: Proto-oncogene vav.

In bold are indicated the proteins belonging to the short signature.

To further ascertain the biological processes and molecular functions associated with each COPD subtype, we performed GO pathway analysis on the Top90 up-regulated proteins in Cluster 2 (FDR<0.0001). Our data highlighted the enrichement of this cluster in hallmarks of lung immunity/host defense, cell fate/repair/remodelling and mitochondrial/metabolic activities (Fig 2D, Table 1 and S3 Table in S1 File).

### Clinical parameters in Cluster 1- and Cluster-2-related COPD patients

We then examined whether COPD patients from the two clusters showed distinct clinical characteristics by considering the parameters describing the COBRA cohort at visit 1 (Table 2). Cluster 2 patients had a lower incidence (p = 0.01) and number (p = 0.02) of exacerbations and of unscheduled medical visits (p = 0.0002) in the previous year, compared to those in Cluster 1 (Table 2). The proportion of COPD patients with emphysema, as assessed by CT scan and/or by the measurement DLCO, was also lower in Cluster 2 than in Cluster 1 (p<0.0001) (Table 2). Further, Cluster 2 patients had a lower prevalence of hypertension (p = 0.02), diabetes (p = 0.009) and obstructive sleep apnea (p = 0.04) (Table 2). In addition, the proportion of patients requiring long-acting β_2_-agonists (LABA) alone was significantly lower in Cluster 2 than in Cluster 1 (p = 0.007) and this was accompanied by a trend towards a lower rate of patients treated with LABA, in combination with long-lasting muscarinic antagonists (LAMA) (p = 0.06) (Table 2). In contrast, the incidence of ICS use, alone or in combination with LABA and LAMA, was not significantly different between the two clusters (p = 0.79) (Table 2).

**Table 2:** Differences in clinical characteristics between COPD patients of Cluster 1 and Cluster 2 at visit 1.

Parameter	Cluster 1—number *	Cluster 2—number *	Values in Cluster 1	Values in Cluster 2	p value **
Male sex—no. (%)	126	115	82 (65)	82 (71)	
Age (years)	126	115	63.7 ± 9.7	62.5 ± 9.9	0.34
Caucasian origin—no. (%)	125	114	115 (91)	101 (88)	0.39
*GOLD stages*					
GOLD I—no. (%)	125	114	23 (18)	31 (27)	0.12
GOLD II—no. (%)	125	114	49 (39)	37 (32)	0.28
GOLD III—no. (%)	125	114	29 (23)	24 (21)	0.76
GOLD IV—no. (%)	125	114	23 (18)	21 (18)	1.00
Body Mass Index (kg per m^2^)	126	115	27.1 ± 6.8	25.8 ± 5.1	0.09
*Smoking history*					
Never smokers—no. (%)	126	115	1 (1)	7 (6)	**0.02**
Active smokers—no. (%)	126	115	46 (37)	38 (33)	0.59
*Biology*					
Blood leukocytes (no. per mm^3^)	97	68	7400 (6450–12500)	7600 (6525–8875)	0.42
Blood eosinophils (no. per mm^3^)	97	68	168 (118–228)	158 (88–240)	0.80
With blood eosinophils ≥ 300 per mm^3^—no. (%)	97	68	15 (15)	12 (18)	0.83
Hemoglobin—g per deciliter	84	31	14.6 (13.7–15.2)	14.1 (13.2–15.7)	0.74
CRP—mg per Liter	85	27	4.0 (3.0–8.9)	5.0 (2.3–7.5)	0.79
With CRP ≥ 3 mg per Liter—no. (%)	85	27	65 (76)	23 (85)	0.43
*Respiratory function*					
Pre-bronchodilator FEV_1_ (% predicted)	122	76	58.0 ± 21.1	61.3 ± 27.3	0.36
Post-bronchodilator FEV_1_ (% predicted)	111	95	60.8 ± 20.9	66.0 ± 28.9	0.22
Pre-bronchodilator FEV_1_ / FVC (% predicted)	122	80	52.4 ± 13.7	54.2 ± 16.9	0.51
Post-bronchodilator FEV_1_ / FVC (% predicted)	113	96	52.2 ± 14.1	55.3 ± 17.6	0.21
FRC—%	43	81	138.9 ± 49.3	137.2 ± 31.2	0.82
RV—%	49	86	160.4 ± 61.1	154.1 ± 48.1	0.51
TLC—%	52	85	114.0 ± 23.1	113.6 ± 16.5	0.92
DLCO—%	42	73	54.4 ± 22.6	61.7 ± 20.9	0.08
*Symptoms*					
With emphysema no. (%)	126	115	65 (52)	27 (31)	**< 0.0001**
With exacerbations in the previous 12 months—no. (%)	126	115	77 (61)	51 (44)	**0.01**
Number of exacerbations in the previous 12 months—no.	126	115	1.61 ± 0.25	1.24 ± 0.20	**0.02**
With unscheduled medical visits in the previous 12 months—no. (%)	126	115	67 (53)	38 (33)	**0.0002**
With hospitalizations for COPD in the previous 12 months—no. (%)	126	115	31 (25)	17 (15)	0.08
*Comorbidities*					
Cardiovascular—no. (%)	126	115	61 (48)	42 (37)	0.07
Hypertension—no. (%)	126	115	49 (39)	28 (24)	**0.02**
Diabetes—no. (%)	126	115	21 (17)	7 (6)	**0.009**
Obstructive sleep apnea—no. (%) *Treatments*	126	115	17 (13)	5 (4)	**0.04**
On SABA—no. (%)	125	106	85 (68)	59 (56)	0.06
On LABA alone—no. (%)	125	108	20 (16)	6 (6)	**0.02**
On LAMA alone—no. (%)	125	108	9 (7)	7 (6)	0.83
On ICS alone—no. (%)	125	108	1 (1)	3 (3)	0.25
Daily dose of ICS alone—μg of equivalents beclomethasone	1	3	1000	517 ± 275	0.27
On OCS—no. (%)	125	108	4 (3)	4 (4)	1.00
Daily dose of prednisone (mg)	125	108	24.5 ± 10.4	16.3 ± 11.1	0.32
On LABA + LAMA—no. (%)	125	108	15 (12)	7 (6)	0.06
On LABA + LAMA + ICS—no. (%)	125	108	44 (35)	34 (31)	0.55
On anti-hypertensive drugs—no. (%)	124	108	52 (42)	28 (26)	**0.01**
On statins—no. (%)	124	108	37 (30)	19 (18)	**0.03**
Other—no. (%)	124	107	66 (53)	36 (34)	**0.003**
Adherence to treatment—no. (%)	124	100	107 (86)	96 (96)	**0.02**

Data are expressed as numbers (%) and as means ± SD

DLCO = transfer factor of the lung for carbon monoxide; ICS = inhaled corticosteroids; SABA = short-acting b2-agonists; LABA = long-acting b2-agonists; LAMA = long lasting muscarinic antagonists.

* indicates the number of patients with each available variable;

** Students’ t test or Fisher exact test 2-tailed

Finally, the proportion of COPD patients necessitating anti-hypertensive drugs (p = 0.01), statins (p = 0.03), or other therapies (p = 0.003) was lower in Cluster 2 than in Cluster 1 (Table 2). No difference was observed between the two clusters in terms of gender, age, ethnic origin, GOLD stages, smoking history, biology (including serum levels of CRP), respiratory function, symptoms and treatements (Table 2). Failure to observe differences in these parameters may be linked, at least in part, to the higher proportion of COPD patients belonging to GOLD I and II stages in each cluster. For example, when considering the distribution of values of pre-bronchodilator FEV_1_ across COPD severity, 56% and 61% patients belonged to GOLD I and II in Cluster 1 and 2, respectively (S1 Fig).

### Correlations between cluster-associated proteins and key clinical parameters

Correlation analyses showed positive associations between lower incidence of exacerbations and/or prevalence of emphysema and circulating levels of different biomarkers implicated in the regulation of EGFR pathway (*eg*. SHC1, AREG, GRB2 adapter protein), of host defense and innate immune responses (BTK), oxidant stress (*eg*. cyclophilin F, α-Synclein and Carbonic anhydrase XIII, 14-3-3 protein β/α and ζ/δ, BAD), wound healing and cell survival (*eg*. Tropomyosin 4, 14-3-3 protein β/α and ζ/δ, eIF-5A-1, FGF-16, PA2G4, TCTP, BAD), as well as some components of epithelial-mesenchymal remodelling (*eg*. RAC1, GRB2 adapter protein, ARGI1, Prostatic binding protein, DRG-1, CPNE1). Positive correlations were also found between lower prevalence of emphysema and levels of biomarkers of T cell activation (CD40 ligand), metabolism (M2-PK and 6-phosphogluconate dehydrogenase) and proteostasis (*eg*. Ubiquitin^+1^, SUMO3, UBC9, Sorting nexin 4, SNAA, UFM1) markers (S4 Table in S1 File).

Finally, a negative association was found between serum levels of MMP-12 and renin, which were up-regulated in Cluster 1, and lower prevalence of emphysema (S4 Table in S1 File).

### Examination of Clusters 1 and 2 in an independent COPD cohort and in healthy subjects

**Fig 3:**
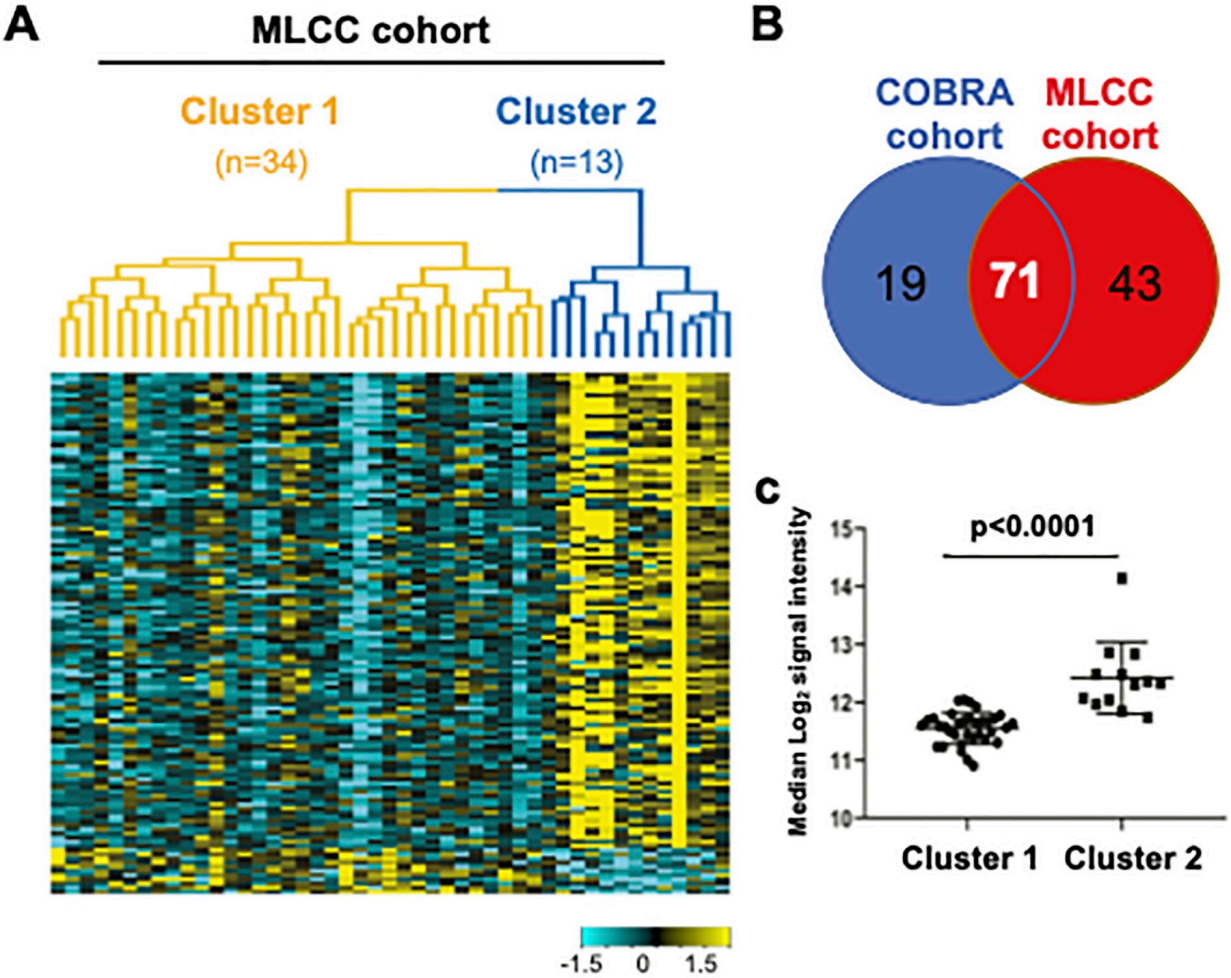
COPD Clusters are observed in an independent patient cohort, but not in healthy controls. (A) Heatmap showing levels of differentially expressed proteins in the MLCC cohort. Yellow indicates high expression and cyan indicates low expression. (B) Overlap of the Cluster 2-associated up-regulated proteins (Fold change >1.5 for Cluster 2 *vs* Cluster 1 and FDR <0.05) between COBRA and MLCC cohorts. (C) Protein score (defined in CORBA cohort) in Cluster 1- and 2 in the MLCC cohort.

We then attempted to validate these clusters in another COPD population of 47 patients [21]. Unsupervised hierarchical clustering, with the top 10% proteins by expression variation, showed two clusters within these COPD subjects (34 and 13 subjects in Clusters 1 and 2, respectively) where 125 proteins were differentially expressed between Cluster 1 and Cluster 2 (Fold change>1.5 and FDR<0.05) (Fig 3A). While 114 proteins were significantly up-regulated in Cluster 2, 71 of these (62%), overlapped with the proteins that were higher in Cluster 2 of the COBRA cohort at visit 1 (Fig 3B). We also found no similarities between the 2 cohorts regarding the few up-regulated proteins in Cluster 1. We calculated the protein score (median expression of the 90 Cluster 2-associated proteins defined in the COBRA cohort), and showed that it was significantly up-regulated in Cluster 2, as compared to Cluster 1 in the MLCC cohort (p<0.0001) (Fig 3C).

Comparisons of the clinical parameters between the two clusters showed significant lower prevalence of emphysema and of incidence of hospitalizations for COPD during the last 12 months, as well as a diminished requirement of treatment with LABA, in Cluster 2 compared to Cluster 1 (S6 Table in S1 File). This was accompanied by a trend towards a lower onset of exacerbations and of cardio-metabolic co-morbidities, although the differences between the two clusters failed to achieve statistical significance (S5 Table in S1 File).

To determine whether the subtyping was specific of COPD, we performed the same analyses in serum samples from a group of 50 healthy donors. Although hierarchical clustering generated two subtypes within these subjects, the protein score, as defined in the COBRA cohort, failed to show significant difference between the 2 Clusters (median log2 signal intensity between Cluster 1 (n = 19 subjects) and Cluster 2 (n = 31 subjects) [5%-95%]: 10.6 [10.5–10.7] and 10.7 [10.6–10.8], respectively, p = 0.129).

### Identification of a protein fingerprint associated with COPD endotypes

To establish a short protein signature reflecting changes in clinical outcomes in relation with lung biological processes, we generated a fingerprint of 15 biologically relevant biomarkers of the top 96 differentially expressed proteins that were mainly selected from the different GO biological processes previously identified (Fig 2B, S3 and S4 Tables in S1 File). These biomarkers included 11 and 4 proteins representative of Cluster 2 and 1, respectively (Table 3).

**Table 3:** Correlation analyses between the 15 differentially regulated proteins and main clinical parameters in COPD patients of the COBRA cohort.

Biological processes	Target	With exacerbations (Y/N)		With emphysema (Y/N)		With unscheduled medical visits (Y/N)	
		OR (2.5–97.5%)	FDR	OR (2.5–97.5%)	FDR	OR (2.5–97.5%)	FDR
**Cell fate, remodeling and repair**	**AREG**	0.78 (0.53–1.15)	0.310	0.49 (0.3–0.8)	**0.004**	0.69 (0.45–1.05)	0.122
	**FGF16**	0.81 (0.55–1.19)	0.347	0.54 (0.34–0.85)	**0.007**	0.74 (0.5–1.1)	0.164
	**SHC1**	0.64 (0.4–1.03)	0.102	0.33 (0.19–0.59)	**0.001**	0.58 (0.36–0.95)	0.057
	**14-3-3 protein β/α**	0.51 (0.32–0.81)	**0.026**	0.4 (0.24–0.68)	**0.002**	0.46 (0.28–0.75)	**0.015**
	**eIF-4H**	0.63 (0.46–0.86)	**0.026**	0.58 (0.41–0.82)	**0.003**	0.62 (0.45–0.86)	**0.015**
	**Tropomyosin 4**	0.78 (0.64–0.96)	**0.042**	0.71 (0.57–0.89)	**0.004**	0.78 (0.63–0.96)	**0.049**
**Metabolism and mitochondria**	**Cyclophilin F**	0.72 (0.57–0.91)	**0.026**	0.65 (0.5–0.84)	**0.002**	0.69 (0.54–0.88)	**0.015**
	**Carbonic anhydrase XIII**	0.80 (0.65–0.99)	0.068	0.74 (0.59–0.94)	**0.013**	0.77 (0.62–0.96)	**0.049**
	**H2A3**	0.87 (0.69–1.09)	0.310	0.81 (0.63–1.04)	0.099	0.87 (0.69–1.1)	0.268
**Immunity and defense**	**BTK**	0.72 (0.57–0.92)	**0.026**	0.63 (0.49–0.82)	**0.002**	0.73 (0.57–0.93)	0.035
	**CD40 ligand**	0.91 (0.64–1.3)	0.598	0.54 (0.35–0.81)	**0.004**	0.72 (0.5–1.05)	0.127
	**Midkine**	1.4 (1.06–1.84)	**0.042**	1.45 (1.08–1.94)	**0.012**	1.32 (1–1.74)	0.093
	**Lactadherin**	1.57 (1.07–2.3)	**0.042**	1.97 (1.31–2.95)	**0.002**	1.38 (0.94–2.01)	0.131
**Tissue injury**	**MMP-12**	1.13 (0.82–1.55)	0.484	1.45 (1.04–2.03)	**0.031**	1.11 (0.81–1.53)	0.561
	**Renin**	1.10 (0.86–1.41)	0.484	0.99 (0.77–1.27)	0.933	1.07 (0.84–1.37)	0.567

*Abbreviations*: DLCO, transfer factor of the lung for carbon monoxide; OR, odds-ratio; FDR, false discovery rate; AREG, Amphiregulin; FGF16, Fibroblast growth factor 16; SHC, SHC-transforming protein 1; eIF-4H: Eukaryotic translation initiation factor 4H; BTK, Tyrosine-protein kinase BTK; MMP-12, metallopreoteinase-12.

OR was obtained through a logistic model.

Bold denotes statistical significance

Specifically, Cluster 2-associated Metabolism/Mitochondria markers, namely Cyclophilin F, Carbonic anhydrase XIII and H2A3, were sorted from GO pathways involved in mitochondrial membrane organization and permeability and cellular response to oxidative stress and to environmental stimulus, respectively, whereas immunity/defense markers, including BTK and CD40 ligand, were sorted from activation of innate immune response and T cell regulation, respectively (S4 Table in S1 File).

To cover the different aspects of cell fate/repair/remodeling processes, rational ranking of differentially expressed proteins led to the prioritisation of: 1) the representative transcription factor, eIF-4H, that is involved in the post-translational regulation of gene expression; 2) the two growth factors, AREG and FGF-16, that promote tissue regeneration through ERBB signaling pathway and that positively regulate cell proliferation; 3) the cytosolic protein regulating cell proliferation, SHC1, 4) the key regulator of apoptotic and nutrient-sensing signaling, 14-3-3 protein β/α; and 5) the tissue-remodeling mediator, Tropomypsin 4, that contributes to actin filament organization. Lastly, to predict Cluster 1-COPD endotype, a selection of 4 of the 6 up-regulated proteins in Cluster 1 (S3 Table in S1 File), was performed, after exclusion of C3b that failed to show any differential expression in the MLCC cohort. These 4 proteins included two hallmarks of tissue injury (MMP-12 and renin) and two modulators of lung immune responses (Midkine and Lactadherin).

We next investigated the association between each of the 15 proteins composing this short signature and main hallmarks of COPD, such as the prevalence of emphysema, the incidence of exacerbations and of unscheduled medical visits and the number of exacerbations and values of DLCO recorded during the previous 12 months (Table 3). We showed that 7 and 5 out of the 15 proteins were associated with the incidence of exacerbations and with that of unscheduled medical visits, respectively, and that 12 of the 15 proteins correlated with the prevalence of emphysema (Table 3). The proteins that displayed the most significant correlations with exacerbations and unscheduled medical visits were 14-3-3 protein β/α, eIF-4H, that regulate cell renewal by controlling apoptosis, and cyclophilin F, a marker of metabolism and of mitochondrial functions (FDR values between 0.015 and 0.026) (Table 3). The 12 proteins associated with the prevalence of emphysema fitted wth all biological processes and they showed FDR values ranging from 0.03 and 0.001 (Table 3). In contrast, we found no significant correlation between the expression of each of the 15 proteins and the number of exacerbations, or DLCO values (rho between 0.01 and 0.11, p values from 0.79 and 0.94, for exacerbations, and rho between 0.02 and 0.15, p values ranging from 0.90 and 0.38, for DLCO, Pearson correlation).

### Monitoring cluster switching at a second visit

To determine whether the 2 protein clusters were maintained in the 163 subjects from COBRA cohort during the follow-up visit, we first performed unsupervised hierarchical clustering on these subjects. Consistent with the results at inclusion, the two distinct expression patterns were confirmed at visit 2 (97 subjects in Cluster 1 and 66 subjects in Cluster 2) (S1A and S1B Fig). The expression of 86 proteins was significantly different between the two clusters (Fold change>1.5 and FDR<0.05), with 83 and 3 up-regulated proteins in Cluster 2 and Cluster 1, respectively, when compared to their Cluster 1 and 2 counterparts. Seventy-five out of these 83 upregulated proteins (*eg*. 90%) were also enriched in Cluster 2 at visit 1, whereas only one of the 3 up-regulated proteins in Cluster 1 (*eg*. Lactadherin) was also elevated at visit 1. Furthermore, the protein score (median expression) of the 90 Cluster 2-associated proteins defined at visit 1 was significantly higher in Cluster 2 at visit 2, as compared to Cluster 1 at visit 1 (p<0.0001, S1C Fig).

Next, we demonstrated that clustering pattern was significantly consistent between the two visits, with 108 subjects among 163 showing the same cluster identify (68 for Cluster 1 and 40 for Cluster 2) (p<0.001, Fisher exact test). However, 55 subjects changed their cluster identity, since 29 patients belonging to Cluster 1 at visit 1 exhibited mixed profile combining Cluster 1 and 2 protein signatures at visit 2 and 26 switched from Cluster 2 at visit 1 to Cluster 1 at visit 2 (S6 Table in S1 File). COPD patient switching from to Cluster 1 to 2 displayed lower incidence of exacerbations (p = 0.02), of unscheduled medical visits (p = 0.04) and of hospitalizations for COPD (p = 0.02) in the previous year. COPD patients switching from Cluster 2 to 1 showed a significant higher incidence of hospitalizations for COPD (p = 0.05) and of LAMA use (p = 0.04) (S6 Table in S1 File).

## Discussion

Using SOMAscan, we profiled the serum levels of 1305 proteins in 241 COPD patients from the COBRA cohort and identified two distinct subtypes throught unsupervised hierarchical clustering of 96 differential expressed proteins. These two clusters appeared to be clinically relevant since Cluster 2 showed lower number and incidence of exacerbations, unscheduled medical visits, hospitalizations for COPD within the previous year and reduced prevalence of emphysema than Cluster 1.

Lung function was similar between the 2 clusters, which could be explained by the higher proportion of GOLD I and II patients in the COBRA cohort, who manifested little or no airflow obstruction.

The occurrence of co-morbidities, particularly hypertension, diabetes and obstructive sleep apnea, the need of LABA of treatements for cardiovascular and metabolic comorbidities (*i*.*e*., anti-hypertensive drugs and statins), were all lower in Cluster 2 than in Cluster 1.

Despite the limited number of COPD patients included in the MLCC patient group, we were able to cross-validate, at least in part, our findings by showing a reduction in the prevalence of emphysema and on the incidence of hospitalizations for COPD during the last 12 months and a trend towards a lower occurrence of exacerbations and of cardio-metabolic co-morbidities months in Cluster 2 *versus* Cluster 1. Therefore, our results highlight the valuable use of SOMAscan technology as reproducible screening tool to identify specific serum biomarkers associated to COPD phenotypes.

To initiate mechanism understanding, we combined GO pathway analysis with mapping of the 96 differentially expressed proteins into biological processes. This approach led us to characterize the enrichment of Cluster 2 with hallmarks of lung immunity/host defense, cell fate/repair/remodelling and mitochondrial/metabolic activities. These findings reflect high glycolytic activity, mitochondrial functions and proteostasis that may maintain mucosal barrier by regulating metabolic energy in proliferating immune and structural cells upon tissue remodelling under oxidative stress in Cluster 2 [24]. Therefore, these results are consitent with proteomic profiling of the bronchoalveolar lavages from COPD and lung cancer patients [25].

To distinguish between the different COPD clusters, the top96 differentially expressed proteins were refined into a clinically feasible fingerprint composed of 15 biologically biomarkers, including 11 and 4 up-regulated proteins in Cluster 2 and Cluster 1, respectively. The 11 up-regulated biomarkers in Cluster 2 have been reported to promote tissue repair and they included the pro-survival protein, 14-3-3 protein β/α, the transcription factor-regulating stem cell renewal, eIF4H, and the growth factors-promoting stem cell activity, AREG, SHC1 and FGF-16. These findings suggest that Cluster 2-patients exhibit better lung regeneration than those in Cluster 1. Of note, the mesenchymal biomarker, Tropomyosin 4, and Carbonic anhydrase XIII, one of the main hallmarks of systemic and local oxidative stress, were also augmented in Cluster 2 patients. Previous studies have shown upregulation of Tropomyosin 4 expression in muscle fibres of diaphragm of COPD patients under enhanced resistance to fatigue [26] and elevated levels Carbonic anhydrase XIII in COPD skeletal muscles in relation with a gain of force [27]. Together, these results suggest that enrichement of Tropomyosin 4 and of Carbonic anhydrase XIII in Cluster 2 translated into a better efficiency of the antioxidant systems to improve muscle dysfunction in response to chronic exercise [28]. Representative hallmarks of proteostasis (*eg*. Cyclophilin F, Carbonic anhydrase XIII and H2A3), and of innate immune response, host defense and inflammasome activity (BTK and CD40 ligand) [29–31] were also higher in Cluster 2 than in Cluster 1. Given that Cyclophilin F, an ubiquitously expressed immunophilin involved in protein folding/trafficking and mitochondrial permeability [32], can be secreted under inflammatory stimuli and oxidative stress [33], its elevated expression, along with BTK and CD40 ligand, may indicate an improved immune/inflammatory responses upon lung injury and oxidative stress in Cluster 2.

Finally, among the 4 proteins of our fingerprint that were up-regulated in Cluster 1, Midkine and Lactadherin, were reported to be prominent during several chronic immune and metabolic disorders, such as atherosclerosis, cardiac, kidney and metabolic diseases [34–36]. Elevated levels of these proteins in Cluster 1 COPD patients are consistent with higher incidence of hypertension, diabetes and use of anti-hypertensive drugs and statins and they support the contribution of cardiometabolic co-morbidities to COPD severity [6, 37]. MMP-12 levels were also higher in Cluster 1 than in Cluster 2 and they were associated with greater prevalence of emphysema. This finding is consistent with MMP-12 involvement in lung injury secondary to the degradation of extracellular matrix in COPD [38, 39]. Likewise, elevated renin expression, an enzyme involved in the renin-angiotensin II-aldosterone axis regulating blood pressure [40], was associated with higher prevalence of hypertension in Cluster 1, as previously demonstrated [41].

Correlation analyses between each of the 15 proteins and major clinical determinants of COPD demonstrated that our signature reflected mainly the prevalence of emphysema, rather than the incidence of exacerbations and of unscheduled medical visits, although 5 to 7 proteins out of the 15 and related to cell fate, remodeling and repair (14-3-3 protein β/α, eIF-4H and tropomyosin 4) and to immunity and defense (BTK, midkine and lactadherin) were associated with these two latter clinical parameters.

Importantly, 12 proteins belonging to all classes of biological processes were highly-significantly associated with emphysema. These included AREG, FGF16, SHC1, 14-3-3 protein β/α, eIF-4H and tropomyosin 4 (cell fate remodeling and repair), cyclophilin F and carbonic anhydrase XIII (metabolism and mitochondria), BTK, midkine and lactadherin (immunity and defense) and MMP-12 (tissue injury). These data indicated that lung injury and renewal, oxidative stress, diaphragm muscle dysfuntions, immune responses and subcellular alterations, predominated in our patient cohort in association with emphysema.

Intriguingly, we found no association between the protein fingerprint and values of DLCO. However, in the COBRA cohort, emphysema was not monitored homogeneously across the hospital centers participating in patient inclusion, since either quantitative computed tomography (CT) scan, or DLCO was used. The latter being also a marker of altered alveolar-capillary permeability, its use to map emphysema may have confounded the analyses and explain this discrepancy.

Serum proteomic profiling at visit 2 showed that approximately 34% of COPD COBRA patients changed their clusters with no link to acute events (*eg*. exacerbations, pneumonia), or co-morbidities, all patients being in a stable state during the month preceding blood sampling. Patients exhibiting combined Cluster 1 and Cluster 2 protein profiles (18%) displayed significant lower incidence of exacerbations, of unscheduled medical visits and of hospitalizations for COPD, whereas switching from Cluster 2 to Cluster 1 (16% of the patients) was associated with an increase in the onset of hospitalizations for COPD and with a trend towards higher LAMA use.

Whereas emphysema status did not improve in patients with combined protein profiles, the increase in markers of host defense and lung repair after 6–12 months at visit 2, suggested a better recovering of the lung tissue, potentially as a result of the activation of repairing mechanisms. Whether the enhancement in lung repair can translate into a long-term improvement of lung function within this group of patients, requires further investigation.

Among the 15 protein fingerprint that correlated with the prevalence of emphysema and the incidence of exacerbations, FGF-16, a growth factor contributing to tissue regeneration [42, 43], may represent a starting point for the development of new therapeutics. Consistently, the airway administration of FGF-2, another member of FGF family promoting tissue repair, was reported to reduce emphysema and to enhance lung repair in cigarette smoke-exposed or elastase-induced COPD mouse models, possibly by attenuating inflammation and alveolar cell death [44]. In addition, a few randomized clinical trials performed in patients with periodontitis and osteoarthritis, two chronic inflammatory diseases with progressive tissue degeneration, demonstrated improved tissue repair upon the administration of recombinant human FGF-2 [45, 46]. Whether FGF-16 promotes similar beneficial effects in COPD patients deserves further investigation.

In an effort to profile COPD patients, specific markers have been previously established for identifying patients with high *versus* low rate of exacerbations [10], or by defining COPD-subtypes using CT imaging, namely, emphysema- and airway-dominant diseases [11, 12]. In an extention of these latter studies, CT-based phenotying was shown to be associated with gender signatures involving different types of leukocytes and of mitochondrial-related genes, as assessed in bronchial brushes [13]. These generated profiles, however, have not been validated across multiple cohorts. An additional integrative sputum microbiome analysis stratified COPD patients into two neutrophilic subgroups differing by the predominance of airway *Haemophilus* infection and by the interchangeability with eosinophilic inflammation [14], suggesting that different therapeutic strategies may succesfully target these phenotypes. Of significance, our study provides novel insights into COPD heterogeneity and suggests that overexpression of factors involved in lung immunity/host defense, cell fate/repair/remodelling and mitochondrial/metabolic activities contribute to better clinical outcomes. Despite the non-invasive nature of our COPD phenotyping through SOMAscan proteomic analysis applied to serum samples, this study has some limitations.

Firstly, SOMAscan can detect very low levels of proteins compared to immunoassays, but, as a discovery tool, this platform provides only relative quantification, rather than absolute concentrations. Although this technology is rapidly growing in popularity due to the quantification of large numbers of proteins efficiently and cost-effectively, comparisons to conventional immunoassays are required potentially due to lack of specificity for some aptamers, or differences in signal to noise ratios.

Secondly, the association studies between the different protein signatures and the COPD clusters as shown herein suggest that distinct molecular mechanisms of lung inflammation, injury/repair and oxidative stress operate differently in Cluster 1 and Cluster 2-COPD patients. Whether our cluster-specific protein signatures can predict disease pathophysiology and progression requires further investigations.

Thirdly, we were able to cross-validated our findings only in a small group of COPD patients belonging to the MLCC cohort, that included mainly frequent exacerbators. Therefore, it would be appropriate to further confirm the existence of the 2 COPD clusters currently described in a larger series of fully-clinically defined COPD patients. Also, additional tissue validation studies using patient biopsies, or primary lung cells, are required to confirm the presence of our 15-protein signature.

Fourthly, exacerbations were reported at 6- or 12-month intervals in the COBRA cohort and relied on self-reporting. This method could lead to underreporting of mild or moderate events that may manifest potentially in patients switching clusters in the COBRA cohort, but is unlikely to influence the identification of severe exacerbators, as those included in MLCC cohort.

Lastly, the nature of exacerbation triggers, such as viral, or bacterial respiratory cultures, air pollution and others, that may influence the cluster switch, is not detailed in either cohorts, COBRA and MLCC.

## Conclusion

Overall, this study demonstrates that the SOMAscan technique offers a powerful and reproducible screening tool to identify specific serum proteins and pathways that allows a better understanding of the largely unknown heterogeneity of COPD and the characterization of novel endotypes. In addition, the currently described short protein fingerprint may lead to a better management of COPD patients in terms of treatment options and monitoring, with more frequent follow-up visits for the patients belonging to Cluster 1. Also, this signature may offer a valuable tool for selecting patients to be included in clinical trials and for identifying potential new therapeutic targets.

## Supporting information

S1 File Methods and supplementary tables. (PDF)

S1 Table Characteristics of COPD patients of the COBRA cohort. (PDF)

S2 Table Characteristics of COPD patients of the MLCC cohort. (PDF)

S3 Table Changes in the serum levels of the significantly-regulated proteins between COPD patients from Cluster 1 and Cluster 2. (PDF)

S4 Table Significant correlations between differentially regulated proteins and the incidence of exacerbations and emphysema in patients with COPD at visit 1. (PDF)

S5 Table Differences in clinical characteristics COPD patients from the MLCC cohort between Cluster 1 and Cluster 2 at inclusion. (PDF)

S6 Table Main clinical characteristics of COPD patients switching of Clusters between visit 1 and visit 2. (PDF)

S1 Fig Differetially expressed proteins in the COBRA cohort at visit 2. (TIFF)

S2 Fig Values of pre-bronchodilator FEV1 in Cluster 1 and Cluster 2, according to GOLD stages. (TIFF)
