# Supplementary material for: Proteomic profiling of serum identifies a molecular signature that correlates with clinical outcomes in COPD
Source: PLoS One. 2022 Dec 8;17(12):e0277357. doi: 10.1371/journal.pone.0277357 (PMC9731494; doi:10.1371/journal.pone.0277357)
Supplement: S1 File — (PDF) [file pone.0277357.s001.pdf]

Supporting information

Methods

Study populations

Between March 2007 and November 2016, 241 patients with stable COPD aged between 26 and 82 years and without concurrent asthma were included in the COBRA cohort, a multicenter prospective cohort that recruits patients with asthma and COPD from 15 University Pulmonology Departments in France (S1 Table) [S1,S2]. COPD patients were considered stable since they did not report any exacerbation, as defined below, within the month before each visit.

Patients were managed according to the best standard care of tertiary hospital's outpatient clinics in each centre participating in the study. This cohort was approved by the CPP Ile-de-France I Ethics Committee (n° 09-11962) and all subjects gave their written informed consent before being enrolled.

During the first visit (inclusion), demographic data, smoking history, peripheral cell counts, number of exacerbations, of unscheduled hospital visits and hospitalizations for COPD in the previous year, symptoms (cough and wheezing), pulmonary and extra-pulmonary co-morbidities and baseline medications were recorded in a validated standardized folder (S1 Table).

In addition, all patients underwent spirometry, and forced expiratory volume in one sec (FEV_1) and forced vital capacity (FVC) were monitored before and after the inhalation of 400 µg of salbutamol. Functional Residual Capacity (FRC), Residual Volume (RV) and Total Lung Capacity (TLC) were also determined. Lung emphysema was assessed by computed tomography (CT) and by the measurement of transfer

factor of the lung for carbon monoxide (DLCO), the latter correlating well with CT-determined emphysema for all Global Initiative for Obstructive Lung Disease (GOLD) stages [S3,S4].

COPD severity was graded into stage I (22.7%), II (36.5 %), III (22.3%) and IV (18.5%), following GOLD [S5] (S1 Table). Most of the COPD patients were former smokers (61.8%) and they showed airflow obstruction with mean values of pre- and post-bronchodilator FEV_1 lower than 70% of predicted and of pre- and post-bronchodilator FEV_1 to FVC ratio lower than 60%. Approximately 60% of the patients had fixed airflow obstruction, with values of post-bronchodilator FEV_1/FVC lower than 70% [S5] and 38.2% of them exhibited emphysema on CT scan. Around 40% of the patients experienced cough and 13.7 % had wheezing.

The majority of the patients (between 62.3 and 69.1%) were treated with short- and long-acting β2-agonists (SABA and LABA, respectively), long-acting muscarinic antagonists (LAMA, 52.4%) and inhaled corticosteroids (ICS, 52.8%). These drugs were administered alone, or in combination (LABA + LAMA, or LABA + ICS + LAMA) (S1 Table).

As previously reported [S6-S9], principal co-morbidities were of cardiovascular origin (42.7%), but also metabolic disorders, such as dyslipidemia (24.1%) and diabetes (11.6%). A sub-group of COPD patients also manifested allergic rhinitis and sinusitis (15.8 and 11.2%, respectively) and gastro-esophageal reflux (29%). Because of the high frequency of cardiovascular disorders and dyslipidemia, 34.5% of the patients were treated with anti-hypertensive drugs and 24.2% with statins. The proportion of patients adhering to treatments was of more than 90% (S1Table).

Exacerbations were defined as "acute worsening of respiratory symptoms that results in additional therapy". Patients were classified as mild, if they were treated with SABA only, moderate if they were treated with LABA plus antibiotics and/or oral corticosteroids (OCS) bursts, or severe, if the patient visits the emergency room, or requires hospitalization [S5]. Unscheduled visits were recorded, either to the emergency department or to patient's usual general practitioner.

Peripheral venous blood was obtained in each patient and total and differential leukocyte counts (in numbers per mm^3), levels of hemoglobin (in g per decilitre) and C reactive protein (CRP, in mg per Litre) were assessed. Median numbers of total and differential blood leukocytes were in a normal range and 16.4 % of patients showed numbers of eosinophils ≥ 300 per µL [S10] and 75.9 % had values of CRP ≥ 3 mg/Litre (S1 Table) [S11].

Serum aliquots (250 µL) were prepared and stored at -80°C at the Biological Resources Centre of the Bichat Hospital in Paris (Dr. Sarah Tubiana) for the determination of proteomic profile by SOMAscan. All samples were tracked through a dedicated database.

The evolution in the clinical parameters and in proteomic profiles was also assessed in 163 COPD patients, out of the 241 initially included, since they provided one satisfactory follow-up visit and blood sampling (S1 Table). The time elapsing from the 1st (inclusion) and the 2nd visit was of 7.5 ± 6.6 months (mean ± SD).

SOMAscan data obtained in the COBRA cohort were validated in a separate series of 47 COPD patients originating from the Melbourne Longitudinal COPD Cohort (MLCC) cohort, that was established to study the etiology of frequent exacerbators in

a community setting [S12] (Table S2). These 47 COPD subjects were more severe and symptomatic in comparison to those from the COBRA cohort. Indeed, 61.7% and 40.8% of patients in the MLCC and COBRA cohorts, respectively, belonged to GOLD 3-4 stages (p=0.01, Fisher exact test), 82.6% and 40.2%, respectively experienced cough, 97.7% and 53.0 % of patients were treated with LAMA, 91.5% and 52.8% received long-term ICS and 57.4% and 15.5 % were on oxygen therapy (all p<0.0001) (S1 and S2 Tables). Airflow obstruction was also more severe in MLCC than in COBRA cohort and the prevalence of emphysema was higher in the former than in the latter cohort (94.0 % and 38.2%, respectively, p<0.0001, S2 Table).

Serum samples were also prepared from peripheral blood of n=50 non-COPD, non-asthmatic healthy volunteers who served as controls. These subjects were anonymously enrolled in the AstraZeneca Research Specimen Collection Program and they provided signed informed consent. They were aged of (mean ± SD) 41.2±10.7 years and 80% were of male gender.

SOMAscan proteomic assay, data processing and statistical analysis

Total of 1305 analytes were quantified in patient serum using the SOMAscan high throughput proteomic assay (SomaLogic, Boulder, Colorado, United States of America) at National Jewish Health (Denver, Colorado, United States of America) [S13].

The raw SOMAscan data was standardized by four steps: hybridization normalization, place scaling, median signal normalization and calibration according to manufacturer's instructions (details can be found in SomaLogic's technical notes

(http://somalogic.com/wp-content/uploads/2017/06/SSM-071-Rev-0-Technical-Note-SOMAscan-Data-Standardization.pdf). The standardized expression values were then log2 transformed for downstream analysis. Unsupervised clustering was performed in R, including hierarchical clustering using Pearson correlation as distance measures, and K means clustering with K=2. Functional enrichment was performed with pathway databases including KEGG Reactome and Gene Ontology (GO) pathway database. Differential expression analysis between the two clusters were performed using Limma package [S14]. Proteins with Fold change >1.5 and False discovery rate (FDR)<0.05 was defined as significant. Functional analysis of the differentially expressed proteins were performed based on GO (Biological processes). Additional statistical analysis and plots, including volcano plots and heatmaps, were generated using R, or GraphPad Prism 8.

For two group comparisons, *t* test was performed for data with normal distribution, and Wilcoxon Rank Sum, or Friedman Rank tests was used when normal distribution could not be assumed. Pearson correlation was applied for data with normal distribution and Spearman correlation when normal distribution could not be assumed. ANOVA was used for multi-group comparisons. Fisher exact test and Friedman Rank test were performed for binary data.

Bioinformatical approaches

The bioinformatical approaches are described in Fig 1 in the main manuscript and can be decomposed into three steps:

1. SOMAscan analysis at inclusion: (*i*) hierarchical clustering and identification of contrasted clusters; (*ii*) identification of differentially expressed proteins; (*iii*) GO pathway analysis; *iv*) calculation of a protein score (median expression of identified proteins); (*v*) correlation with clinical parameters; (*vi*) validation of the protein score in an external COPD cohort and in healthy subjects;

2. Identification of short protein signatures associated with each cluster. After exclusion of redundant kinases, phosphatases and adaptor proteins eleven Cluster-2 associated biomarkers were selected by sorting the 10% of the top differentially expressed proteins representative of highly enriched biological processes selected from GO pathway analysis (Tables 1 and S4) performed at visit 1, and exhibited the highest odd-ratios within their corresponding pathways. Cluster-1 associated biomarkers included top four of the 6 up-regulated biomarkers after exclusion of C3b that failed to show any differential expression in the MLCC cohort;

3. follow-up analysis at the second visit: (*i*) hierarchical clustering and identification of contrasted clusters; (*ii*) identification of differentially expressed proteins and assessment of overlap with proteins found at inclusion; (*iii*) monitoring of stable and unstable COPD patients that switched their clusters at follow-up visit using the protein signature.

References

S1. Pretolani M, Soussan D, Poirier I, Thabut G, Aubier M; COBRA Study Group; COBRA cohort Study Group. Clinical and biological characteristics of the French COBRA cohort of adult subjects with asthma. *Eur Respir J*. 2017 Aug 24;50(2):1700019. doi: 10.1183/13993003.00019-2017. PMID: 28838976.

S2. Bourdin A, Suehs CM, Marin G, Vachier I, Matzner-Lober E, Chanez P, Molinari N; COBRA Consortium. Asthma, COPD, and overlap in a national cohort: ACO on a gradient. *J Allergy Clin Immunol*. 2018 Apr;141(4):1516-1518. doi: 10.1016/j.jaci.2017.11.049. Epub 2018 Jan 31. PMID: 29355677.

S3. D'Anna SE, Asnaghi R, Caramori G, Appendini L, Rizzo M, Cavallaro C, et al. High-resolution computed tomography quantitation of emphysema is correlated with selected lung function values in stable COPD. *Respir. Int. Rev. Thorac. Dis.* 2012;83(5):383-90. doi: 10.1159/000329871. Epub 2011 Aug 11. PMID: 21832824.

S4. Criner RN, Hatt CR, Galbán CJ, Kazerooni EA, Lynch DA, McCormack MC, et al. c. *Respir. Res.* 2019 Dec 2;20(1):269. doi: 10.1186/s12931-019-1237-1. PMID: 31791337; PMCID: PMC6889734.

S5. Vogelmeier CF, Criner GJ, Martinez FJ, Anzueto A, Barnes PJ, Bourbeau J, et al. Global Strategy for the Diagnosis, Management, and Prevention of Chronic Obstructive Lung Disease 2017 Report: GOLD Executive Summary. 2017 Apr;22(3):575-601. doi: 10.1111/resp.13012. Epub 2017 Mar 7. PMID: 28150362

S6. Brown JP, Martinez CH. Chronic obstructive pulmonary disease comorbidities. *Curr Opin Pulm Med*. 2016 Mar;22(2):113-8. doi: 10.1097/MCP.0000000000000241.

S7. Hunninghake DB. Cardiovascular disease in chronic obstructive pulmonary disease. *Proc Am Thorac Soc*. 2005;2(1):44-9. doi: 10.1513/pats.200410-050SF. PMID: 16113468.

S8. Cazzola M, Rogliani P, Calzetta L, Lauro D, Page C, Matera MG. Targeting Mechanisms Linking COPD to Type 2 Diabetes Mellitus. *Trends Pharmacol Sci*. 2017 Oct;38(10):940-951. doi: 10.1016/j.tips.2017.07.003. Epub 2017 Aug 7. PMID: 28784329.

S9. Cebron Lipovec N, Beijers RJ, van den Borst B, Doehner W, Lainscak M, et al. The Prevalence of Metabolic Syndrome In Chronic Obstructive Pulmonary Disease: A Systematic Review. *COPD*. 2016 Jun;13(3):399-406. doi: 10.3109/15412555.2016.1140732. Epub 2016 Feb 25. PMID: 26914392

S10. Yun JH, Lamb A, Chase R, Singh D, Parker MM, Saferali A, et al; COPDGene and ECLIPSE Investigators. Blood eosinophil count thresholds and exacerbations in patients with chronic obstructive pulmonary disease. *J Allergy Clin Immunol*. Jun;141(6):2037-2047.e10. doi: 10.1016/j.jaci.2018.04.010. Epub 2018 Apr 28. PMID: 29709670; PMCID: PMC5994197.

S11. Dahl M, Vestbo J, Lange P, Bojesen SE, Tybjaerg-Hansen A, Nordestgaard BG. C-reactive protein as a predictor of prognosis in chronic obstructive pulmonary disease. *Am J Respir Crit Care Med*. 2007 Feb 1;175(3):250-5. doi: 10.1164/rccm.200605-713OC. Epub 2006 Oct 19. PMID: 17053205.

S12. Hutchinson AF, Ghimire AK, Thompson MA, Black JF, Brand CA, Lowe AJ, et al. A community-based, time-matched, case-control study of respiratory viruses and exacerbations of COPD. *Respir. Med*. 2007 Dec;101(12):2472-81. doi: 10.1016/j.rmed.2007.07.015. Epub 2007 Sep 5. PMID: 17822891.

S13. Rohloff JC, Gelinas AD, Jarvis TC, Ochsner UA, Schneider DJ, Gold L, et al. Nucleic Acid Ligands With Protein-like Side Chains: Modified Aptamers and Their Use as Diagnostic and Therapeutic Agents. Mol. *Ther. Nucleic Acids* 2014

Oct 7;3(10):e201. doi: 10.1038/mtna.2014.49. PMID: 25291143; PMCID: PMC4217074.

S14. Ritchie ME, Phipson B, Wu D, Hu Y, Law CW, Shi W, et al. Limma powers differential expression analyses for RNA-sequencing and microarray studies. *Nucleic Acids Res*. 2015 Apr 20;43(7):e47. doi: 10.1093/nar/gkv007. Epub 2015 Jan 20. PMID: 25605792; PMCID: PMC4402510.

Supporting information

S1 Table: characteristics of COPD patients of the COBRA cohort

Parameter	n *	All patients (n = 241)	n *	Patients with 1 visit (n=78)	n *	Patients with 2 visits (n = 163)		p value [a]	p value [b]
		Visit 1		Visit 1 only		Visit 1	Visit 2		
Male sex – no. (%)	241	164 (68.1)	78	50 (64.1)	163	114 (69.9)	-	0.37	-
Age (years)	241	63.1 ± 9.8	78	61.6 ± 11.4	163	63.8 ± 8.9	64.5 ± 9.0	0.14	-
Caucasian origin – no. (%)	239	224 (93.7)	76	70 (92.1)	163	154 (94.5)	-	0.49	-
Other origin – no. (%)	239	15 (6.3)	76	6 (7.9)	163	9 (5.5)	-	0.49	-
GOLD stages									
GOLD I – no. (%)	238	54 (22.7)	75	24 (32.0)	159	28 (17.6)	28 (17.6)	**0.03**	0.99
GOLD II – no. (%)	238	87 (36.5)	75	20 (26.6)	159	66 (41.5)	66 (41.5)	**0.03**	0.99
GOLD III – no. (%)	238	53 (22.3)	75	14 (18.7)	159	38 (23.9)	36 (22.7)	**0.03**	0.99
GOLD IV – no. (%)	238	44 (18.5)	75	17 (22.7)	159	27 (17.0)	29 (18.2)	**0.03**	0.99
Smoking history									
Former smoker – no. (%)	241	149 (61.8)	78	41 (52.6)	163	108 (66.3)	108 (66.3)	**0.002**	1.00
Packs per year in former smokers – no.	147	49.1 ± 23.9	39	49.7 ± 22.2	101	49.7 ± 25.1	49.9 ± 25.1	0.99	0.96
Active smokers – no. (%)	241	84 (34.9)	78	30 (38.5)	163	54 (33.1)	54 (33.1)	**0.002**	1.00
Packs per year in active smokers – no.	82	35.8 ± 21.7	28	35.1 ± 20.8	47	36.0 ± 23.8	34.9 ± 21.9	0.89	0.83
Body Mass Index (kg per m^2)	240	26.5 ± 6.1	77	25.7 ± 5.9	163	26.8 ± 6.2	-	0.21	-
Biology									
Blood leukocytes (no. per mm^3)	165	7500 (6500 - 8900)	43	7400 (6400 - 10000)	96	7350 (6500 - 8750)	7100 (6150 - 8150)	0.37	0.34
Blood eosinophils (no. per mm^3)	165	162 (104 - 230)	43	146 (94 - 213)	96	165 (103 - 225)	162 (117 - 244)	0.37	0.67
With blood eosinophils ≥ 300 per mm^3 – no. (%)	165	27 (16.4)	43	4 (9.3)	96	17 (17.7)	12 (12.5)	0.21	0.32
Blood neutrophils (no. per mm^3)	163	4752 (3840 - 5950)	42	4479 (3782 - 6486)	95	4680 (3835 - 5950)	4560 (3843 - 5265)	0.74	0.39
Blood lymphocytes (no. per mm^3)	165	1830 (1406 - 2280)	43	1920 (1539 - 2438)	96	1648 (1302 - 2190)	1782 (1387 - 2184)	**0.04**	0.49
Blood monocytes (no. per mm^3)	161	560 (444 - 720)	40	603 (469 - 740)	96	532 (404 - 698)	555 (463 - 667)	**0.03**	0.25
Hemoglobin – g per deciliter	115	14.4 ± 1.4	32	14.1 ± 1.6	72	14.6 ± 1.3	14.3 ± 1.4	0.10	0.25
CRP – mg per Liter	112	7.6 ± 7.6	22	6.5 ± 4.6	73	7.8 ± 8.1	6.8 ± 7.5	0.35	0.47
With CRP ≥ 3 mg per Liter – no. (%)	112	85 (75.9)	22	17 (77.3)	73	54 (74.0)	54 (74.0)	0.76	1.00
Blood gases									
PaO_2 - mmHg	181	74.5 ± 14.1	57	74.8 ± 14.2	91	72.9 ± 13.7	74.7 ± 13.2	0.42	0.37
$PaCO_2$ - mmHg	182	39.6 ± 4.8	57	40.0 ± 4.8	90	39.8 ± 4.9	40.0 ± 6.0	0.86	0.86
pH	182	7.42 ± 0.03	57	7.41 ± 0.03	92	7.41 ± 0.03	7.41 ± 0.04	0.78	0.70

S1 Table (continued)

Respiratory function									
Pre-bronchodilator FEV_1 (% predicted)	233	59.6 ± 24.3	72	63.0 ± 27.7	144	56.6 ± 21.7	56.1 ± 21.7	0.10	0.84
Post-bronchodilator FEV_1 (% predicted)	171	63.7 ± 25.8	53	71.3 ± 30.4	70	56.6 ± 22.8	56.5 ± 21.5	**0.005**	0.98
With fixed airflow obstruction – no. (%) [c]	176	143 (81.3)	55	34 (61.8)	70	65 (92.9)	65 (92.9)	**< 0.0001**	1.00
Pre-bronchodilator FVC (% predicted)	233	86.6 ± 21.3	73	86.1 ± 21.9	142	85.0 ± 20.4	84.0 ± 20.4	0.73	0.68
Post-bronchodilator FVC (% predicted)	169	91.5 ± 21.1	51	91.9 ± 21.5	69	91.0 ± 20.4	88.8 ± 21.1	0.83	0.54
Pre-bronchodilator FEV_1 / FVC (% predicted)	235	53.5 ± 15.3	74	57.0 ± 16.7	144	51.4 ± 14.0	51.7 ± 14.2	**0.01**	0.89
Post-bronchodilator FEV_1 / FVC (% predicted)	176	53.9 ± 16.2	55	60.6 ± 17.3	70	47.2 ± 14.4	48.4 ± 14.7	**< 0.0001**	0.62
FRC - %	124	137.8 ± 38.3	49	131.5 ± 31.7	37	140.9 ± 43.4	139.9 ± 41.5	0.27	0.92
RV - %	135	156.4 ± 53.0	53	150.7 ± 43.1	41	165.9 ± 62.9	154.7 ± 56.6	0.19	0.41
TLC - %	137	113.8 ± 19.2	55	112.0 ± 16.9	42	114.1 ± 21.7	112.4 ± 19.5	0.60	0.70
DLCO - %	115	59.0 ± 21.7	46	63.9 ± 21.6	34	49.5 ± 18.8	49.9 ± 18.8	**0.003**	0.94
Symptoms									
With cough – no. (%)	241	97 (40.2)	78	27 (34.6)	163	70 (42.9)	56 (34.4)	0.22	0.12
With wheezing – no. (%)	241	32 (13.3)	78	14 (17.9)	163	18 (11.0)	16 (9.8)	0.14	0.72
With emphysema no. (%) [d]	241	92 (38.2)	78	28 (35.9)	163	64 (39.3)	64 (39.3)	0.62	1.00
With exacerbations in the previous 12 months - no. (%)	241	128 (53.1)	78	39 (50.0)	163	89 (54.6)	62 (38.0)	0.51	**0.003**
Exacerbations in the previous 12 months - no.	241	1.44 ± 0.16	78	1.26 ± 0.22	163	1.52 ± 0.21	1.46 ± 0.23	0.12	0.72
Unscheduled medical visits in the previous 12 months - no.	241	1.19 ± 0.17	78	0.96 ± 0.23	163	1.29 ± 0.22	1.08 ± 0.16	**0.03**	0.09
Hospitalizations for COPD in the previous 12 months – no.	241	0.32 ± 0.05	78	0.41 ± 0.11	163	0.27 ± 0.05	0.19 ± 0.06	0.10	0.17
Comorbidities									
Cardiovascular – no. (%)	241	103 (42.7)	78	32 (41.0)	163	71 (43.6)	-	0.71	-
Hypertension – no. (%)	241	67 (27.8)	78	21 (26.9)	163	46 (28.2)	-	0.84	-
Dyslipidemia – no. (%)	241	58 (24.1)	78	14 (17.9)	163	44 (27.0)	-	0.13	-
Diabetes – no. (%)	241	28 (11.6)	78	7 (9.0)	163	21 (12.9)	-	0.38	-
Sinusitis – no. (%)	241	27 (11.2)	78	7 (9.0)	163	20 (12.3)	-	0.45	-
Allergic rhinitis – no. (%)	241	38 (15.8)	78	12 (15.4)	163	26 (15.9)	-	0.92	-
Obstructive sleep apnea – no. (%)	241	22 (9.1)	78	4 (5.1)	163	18 (11.0)	-	0.14	-
Gastro Esophageal Reflux – no. (%)	241	70 (29.0)	78	21 (26.9)	163	49 (30.1)	-	0.62	-
Bronchial dilatation – no. (%)	241	11 (4.6)	78	3 (3.8)	163	8 (4.9)	-	0.72	-

S1 Table (continued)

Treatments									
On SABA – no. (%)	231	144 (62.3)	70	33 (47.1)	158	110 (69.6)	115 (72.8)	**0.002**	0.54
On LABA alone – no. (%)	233	26 (11.2)	70	5 (7.1)	160	21 (13.1)	18 (11.2)	0.19	0.61
On LAMA alone – no. (%)	233	16 (6.9)	70	4 (5.7)	160	12 (7.5)	12 (7.5)	0.63	1.00
On ICS alone – no. (%)	233	4 (1.7)	70	1 (1.4)	160	3 (1.9)	3 (1.9)	0.82	1.00
Daily dose of ICS - µg of equivalents beclomethasone	119	1304 ± 669	28	1499 ± 811	77	1253 ± 633	1242 ± 626	0.11	0.92
On OCS – no. (%)	232	8 (3.4)	69	7 (10.1)	160	1 (0.6)	2 (1.2)	**0.0003**	0.57
Daily dose of prednisone (mg)	8	20.4 ± 10.9	7	22.6 ± 9.6	1	5	5	0.14	-
On LABA + LAMA – no. (%)	233	22 (9.4)	70	6 (8.6)	160	16 (10.0)	21 (13.1)	0.74	0.39
On LABA + LAMA + ICS – no. (%)	233	78 (33.5)	70	22 (31.4)	160	55 (34.4)	63 (39.4)	0.67	0.36
On anti-histamine – no. (%)	241	20 (8.3)	78	8 (10.3)	162	12 (7.4)	8 (4.9)	0.46	0.36
On theophylline – no. (%)	232	1 (0.4)	69	0 (0.0)	160	1 (0.6)	1 (0.6)	0.52	1.00
On gastro esophageal reflux inhibitors – no. (%)	241	56 (23.2)	78	17 (21.8)	163	39 (23.9)	29 (17.8)	0.72	0.18
On anti-hypertensive drugs – no. (%)	232	80 (34.5)	70	24 (34.3)	160	55 (34.4)	54 (33.7)	0.99	0.91
On statins – no. (%)	231	56 (24.2)	69	11 (15.9)	160	44 (27.5)	49 (30.6)	0.07	0.54
On fibrates – no. (%)	229	5 (2.2)	69	1 (1.4)	157	4 (2.5)	3 (1.9)	0.61	0.71
On anti-platelet aggregation – no. (%)	231	63 (27.3)	69	15 (21.7)	159	47 (29.6)	42 (26.4)	0.23	0.54
Other – no. (%)	227	102 (44.9)	68	20 (29.4)	157	81 (51.6)	75 (47.8)	**0.003**	0.50
On oxygen therapy – no. (%)	238	37 (15.5)	75	11 (14.7)	161	26 (16.1)	16 (9.9)	0.78	0.10
Adherence to treatments – no. (%)	224	203 (90.6)	64	55 (85.9)	155	144 (92.9)	143 (92.3)	0.11	0.83

Data are n (%), or means ± SD, or median (25-75 IQR), or means ± SEM, in case of number of exacerbations, of unscheduled hospital visits and hospitalizations for COPD in the previous 6 months

CRP = C reactive protein; FEV_1 = Forced Expiratory Volume in 1 second; FVC = Forced Vital Capacity; FRC = Functional Residual Capacity; RV = Residual Volume; TLC = Total Lung Capacity; DLCO = transfer factor of the lung for carbon monoxide; ICS = inhaled corticosteroids; SABA = short-acting β2-agonists; LABA = long-acting β2-agonists; OCS = oral corticosteroids; LAMA = long-lasting muscarinic antagonists.

* denotes the number of patients with each available variable

[a] $p \leq 0.05$, between COPD patients with 1 and 2 visits (n=78 and n=163, respectively) (Students' t test, or Mann-Whitney U-test, or Fisher exact test, 2-tailed, or Poisson test).
[b] $p \leq 0.05$, between visits 1 and 2 for n=163 COPD patients (Students' t test, or Mann-Whitney U-test, or Fisher exact test, 2-tailed, or Poisson test).

Supporting information

S2 Table: characteristics of COPD patients of the MLCC cohort

Parameter	n *	All patients, n = 47
Male sex – no. (%)	47	29 (61.7)
Age (years)	47	72.5 ± 9.6
Caucasian origin – no. (%)	47	47 (100)
Other origin – no. (%)	47	0
GOLD stages		
GOLD 1 – no. (%)	47	2 (4.3)
GOLD 2 – no. (%)	47	16 (34.0)
GOLD 3 – no. (%)	47	19 (40.4)
GOLD 4 – no. (%)	47	10 (21.3)
Smoking history		
Never smokers – no. (%)	47	0
Former smoker – no. (%)	47	41 (87.2)
Packs per year in former smokers – no.	47	56.9 ± 32.6
Active smokers – no. (%)	47	6 (12.8)
Packs per year in active smokers – no.	47	89.2 ± 52.1
Body Mass Index (kg per m^2)	31	25.2 ± 6.0
Biology		
Blood leukocytes (no. per mm^3)	45	7800 (6500-9800)
Blood eosinophils (no. per mm^3)	45	200 (100-300)
With blood eosinophils ≥ 300 per mm^3 – no. (%)	45	14 (31.1)
Blood neutrophils (no. per mm^3)	45	5300 (4300-6400)
Blood lymphocytes (no. per mm^3)	45	1600 (1100-2000)
Blood monocytes (no. per mm^3)	45	700 (600-800)
Hemoglobin – g per deciliter	45	13.1 ± 1.6
CRP – mg per Liter	34	1.4 ± 2.9
With CRP ≥ 3 mg per Liter – no. (%)	34	2 (5.9)
Blood gases		
PaO_2 - mmHg	9	66.1 ± 5.4
$PaCO_2$ - mmHg	9	40.5 ± 4.6
Respiratory function		
Pre-bronchodilator FEV_1 (% predicted)	28	39.1 ± 10.5
Post-bronchodilator FEV_1 (% predicted)	29	41.9 ± 14.2
Pre-bronchodilator FVC (% predicted)	28	87.1 ± 16.9
Post-bronchodilator FVC (% predicted)	30	93.1 ± 18.0
Pre-bronchodilator FEV_1 / FVC (% predicted)	28	35.5 ± 9.0
Post-bronchodilator FEV_1 / FVC (% predicted)	29	35.5 ± 10.0
With fixed airflow obstruction – no. (%) [a]	29	28 (96.6)
DLCO (%)	25	41.4 ± 12.4
Symptoms		
With cough – no (%)	46	38 (82.6)
With wheezing – no (%)	47	7 (14.9)
With emphysema no. (%)	25	24 (96.0)
Exacerbations in 12 months - no. (%) [b]	47	1.6 ± 0.7
Hospitalizations for COPD in 12 months - no. [b]	17	0.7 ± 0.7

S2 Table (continued)

Comorbidities		
Cardiovascular – no. (%)	47	22 (46.8)
Diabetes – no. (%)	47	9 (19.1)
Obstructive sleep apnea – no. (%)	47	1 (2.1)
Treatments		
On SABA – no. (%)	47	44 (93.6)
On LABA – no. (%)	43	42 (97.7)
On LAMA – no. (%)	47	25 (53.0)
On ICS – no. (%)	47	43 (91.5)
Daily dose of equivalents beclomethasone	40	842 ± 366
On OCS – no. (%)	47	4 (8.5)
Daily dose of prednisone (mg)	4	4.9 ± 2.3
On oxygen therapy – no. (%)	47	27 (57.4)
Adherence to treatment – no. (%)	12	8 (66.7)

CRP = C reactive protein; FEV_1 = Forced Expiratory Volume in 1 second; FVC = Forced Vital Capacity; DLCO = transfer factor of the lung for carbon monoxide; SABA = short-acting β2-agonists; LABA = long-acting β2-agonists; LAMA = long-lasting muscarinic antagonists; ICS = inhaled corticosteroids = OCS, oral corticosteroids.

Data are n (%), or means ± SD, or median (25-75 IQR), or means ± SEM, for the number of exacerbations

* denotes the number of patients with each available variable

[b] Estimated frequency of exacerbations and hospitalizations based on the events during the study period

S3 Table. Changes in the serum levels of the significantly-regulated proteins between COPD patients from Cluster 1 and Cluster 2

Protein ID	Fold changes	p value *	References
Up-regulated in Cluster 2 versus Cluster 1			
**Tropomyosin 4**	3.093	8.13E-29	S1-S3
**Carbonic anhydrase XIII**	3.005	1.73E-29	S4,S5
**BTK**	2.824	3.08E-33	S6,S7
**Cyclophilin F**	2.694	7.51E-29	S8
CSK	2.549	3.06E-36	S9-S11
FER	2.537	1.26E-28	S12,S13
PKC-B-II	2.535	1.19E-42	S14
SP-D	2.532	2.39E-24	S15
VAV	2.439	5.27E-27	S16-S18
Histone H1.2	2.420	9.64E-20	S19-S22
GRB2 adapter protein	2.417	1.97E-26	S23,S24
**H2A3**	2.344	2.10E-19	S19,S22,S25
IMB1	2.316	6.15E-27	S26
DRG-1	2.284	1.16E-32	S27-S29
SRCN1	2.264	9.30E-21	S30,S31
LYN	2.242	1.72E-29	S32,S33
SMAD2	2.206	3.20E-33	S34, S39, S124, S137
NSF1C	2.196	1.83E-26	S35
LYNB	2.182	4.98E-25	S32,S33
NDP kinase B	2.157	6.04E-29	S36-S38
Caspase-3	2.157	3.50E-34	S39
SBDS	2.112	1.97E-30	S40-S42
PDPK1	2.106	4.36E-31	S43,S44
CK2-A1:B	2.097	2.88E-27	S45,S46
RAC1	2.077	3.08E-33	S47-S50
IF4G2	2.064	5.73E-27	S51,S52
**eIF-4H**	2.051	1.54E-25	S53
Aflatoxin B1 aldehyde reductase	2.045	6.17E-23	S54
Sphingosine kinase 1	2.045	1.28E-27	S55
GAPDH, liver	2.042	8.88E-18	S56,S57
UBE2N	2.034	4.11E-24	S58,S61
α-synuclein	2.011	8.92E-19	S62-S63
SGTA	2.006	3.64E-20	S64
SNAA	1.991	3.45E-23	S65-S68
Haemoglobin	1.966	1.81E-08	S69, S70
METAP1	1.958	3.20E-33	S71,S72
IMDH1	1.957	1.70E-17	S73,S74
PRKACA	1.956	2.18E-30	S75
Annexin I	1.921	2.88E-32	S76,S77

S3 Table (continued)

PKC-A	1.921	2.44E-19	S78
RPS6KA3	1.916	1.57E-33	S79,S80
Histone H2A.z	1.914	5.43E-20	S19,S21,S22,S81,S82
hnRNP A2/B1	1.909	1.84E-29	S83-S85
UFM1	1.888	8.39E-23	S58,S86-S88
FYN	1.886	9.28E-13	S89,S90
RAN	1.861	1.85E-14	S91,S92
Myokinase	1.859	4.43E-11	S93,S94
H2B2E	1.841	4.02E-18	S19,S21,S22,S95
41	1.837	6.00E-16	S96,S97
BARK1	1.835	6.95E-28	S98,S99
M2-PK	1.825	4.82E-15	S100
14-3-3 protein zeta/delta	1.785	1.68E-33	S101-S104
PPID	1.781	2.29E-13	S105-S107
6-phosphogluconate dehydrogenase	1.776	7.82E-8	S108
HXK2	1.775	3.32E-13	S109,S110
PTP-1C	1.756	5.02E-20	S111,S112
DUSP3	1.731	2.39E-25	S113,S114
TCTP	1.723	1.83E-27	S115–S117
Triosephosphate isomerase	1.711	1.39E-23	S118,S119
DLRB1	1.681	8.07E-19	S120,S121
SUMO3	1.680	3.88E-26	S58,S122,S123
PA2G4	1.671	3.51E-15	S124,S125
**14-3-3 protein β/α**	1.669	3.42E-28	S101-S103,S126
PPAC	1.667	1.62E-08	S127,S128
Transketolase	1.660	1.28E-24	S129,S130
UBC9	1.651	2.16E-31	S58,S86,S131
**FGF16**	1.650	3.70E-18	S132,S133
EDAR	1.610	1.56E-10	S134,S135
**SHC1**	1.603	2.71E-25	S136,S137
ARGI1	1.601	2.58E-12	S138-S140
GPVI	1.599	3.60E-13	S141,S142
Cyclophilin A	1.592	3.21E-33	S143,S144
BAD	1.587	3.87E-13	S101,S145
ERK-1	1.566	2.59E-28	S146,S147
PLPP	1.560	9.71E-15	S148,S149
CPNE1	1.560	8.80E-18	S150,S151
BPI	1.554	1.79E-06	S152-S154
NCC27	1.552	1.81E-27	S155,S156
MK01	1.548	2.46E-16	S93,S94
eIF-5A-1	1.544	2.47E-16	S157-S160
NACA	1.539	5.58E-09	S161,S162
**AREG**	1.538	1.51E-13	S163-S165

S3 Table (continued)

ATPO	1.536	1.71E-13	S166
**CD40 ligand**	1.524	2.49E-11	S167-S169
Prostatic binding protein	1.519	1.51E-13	S98,S170-S172
Ubiquitin+1	1.512	3.62E-11	S58
Lactoferrin	1.510	9.58E-09	S173
Azurocidin	1.507	5.01E-10	S174,S175
Sorting nexin 4	1.502	1.39E-13	S176
STAT3	1.502	2.02E-26	S46, S50
Up-regulated in Cluster 1 versus Cluster 2			
Glucagon	0.666	1.36E-07	S177
**MMP-12**	0.652	2.55E-09	S178,S179
**Renin**	0.650	6.58E-06	S180
Lactadherin (MFGM)	0.640	8.42E-14	S181-S183
C3b	0.637	3.26E-05	S184
**Midkine**	0.505	1.63E-17	S185-S187

* False discover rate from group comparisons

Abbreviations are listed in the Table 1 of the main manuscript

Bold indicates the 15 proteins belonging to the short signature

References related to S3 Table

S1. Rostila A, Puustinen A, Toljamo T, Vuopala K, Lindström I, Nyman TA, et al. Peroxiredoxins and tropomyosins as plasma biomarkers for lung cancer and asbestos exposure. *Lung Cancer*. 2012;77:450-459.

S2. Okuda K, Watanabe T, Oda R, Sakane T, Kawano O, Haneda H, et al. Pulmonary inflammatory myofibroblastic tumor with TPM4-ALK translocation. *J Thorac Dis*. 2017;9:E1013-1037.

S3. Zhao X, Jiang M, Wang Z. TPM4 promotes cell migration by modulating F-actin formation in lung cancer. *Onco Targets Ther*. 2019;12:4055-4063.

S4. enry EK, Sy CB, Inclan-Rico JM, Espinosa V, Ghanny SS, Dwyer DF, et al. Carbonic anhydrase enzymes regulate mast cell-mediated inflammation. *J Exp Med*. 2016 22;213:1663-1673.

S5. Ostheimer C, Bache M, Güttler A, Kotzsch M, Vordermark D. A pilot study on potential plasma hypoxia markers in the radiotherapy of non-small cell lung cancer. Osteopontin, carbonic anhydrase IX and vascular endothelial growth factor. *Strahlenther Onkol*. 2014;190:276-282.

S6. de Porto AP, Liu Z, de Beer R, Florquin S, de Boer OJ, Hendriks RW, et al. Btk inhibitor ibrutinib reduces inflammatory myeloid cell responses in the lung during murine pneumococcal pneumonia. *Mol Med*. 2019 15;25:3.

S7. Florence JM, Krupa A, Booshehri LM, Gajewski AL, Kurdowska AK. Disrupting the Btk Pathway Suppresses COPD-Like Lung Alterations in Atherosclerosis Prone ApoE-/- Mice Following Regular Exposure to Cigarette Smoke. *Int J Mol Sci*. 2018;19:343.

S8. Pastor MD, Nogal A, Molina-Pinelo S, Meléndez R, Salinas A, González De la Peña M, et al. Identification of proteomic signatures associated with lung cancer and COPD. *J Proteomics*. 2013;89:227-237.

S9. Zhao T, Liu M, Gu C, Wang X, Wang Y. Activation of c-Src tyrosine kinase mediated the degradation of occludin in ventilator-induced lung injury. *Respir Res*. 2014;15:158.

S10. Li G, Li Y, Zheng S-F, Han Y-B, Bai Q-L, Zhao T. Autophagy in pulmonary macrophages mediates lung inflammatory injury via c-Src tyrosine kinase pathway activation during mechanical ventilation. *Eur Rev Med Pharmacol Sci*. 2019;23:1674-1680.

S11. Kim HI, Lee H-S, Kim TH, Lee J-S, Lee S-T, Lee S-J. Growth-stimulatory activity of TIMP-2 is mediated through c-Src activation followed by activation of FAK, PI3-kinase/AKT, and ERK1/2 independent of MMP inhibition in lung adenocarcinoma cells. *Oncotarget*. 2015;6 :42905-42922.

S12. Dolgachev V, Panicker S, Balijepalli S, McCandless LK, Yin Y, Swamy S, et al. Electroporation-mediated delivery of FER gene enhances innate immune response and improves survival in a murine model of pneumonia. *Gene Ther*. 2018;25:359-375.

S13. Ahn J, Truesdell P, Meens J, Kadish C, Yang X, Boag AH, et al. Fer protein-tyrosine kinase promotes lung adenocarcinoma cell invasion and tumor metastasis. *Mol Cancer Res*. 2013;11:952-963.

S14. Chichger H, Vang A, O'Connell KA, Zhang P, Mende U, Harrington EO, et al. PKC δ and βII regulate angiotensin II-mediated fibrosis through p38: a mechanism of RV fibrosis in pulmonary hypertension. *Am J Physiol Lung Cell Mol Physio*l. 2015 Apr 15;308(8):L827-L836.

S15. Restrepo CI, Dong Q, Savov J, Mariencheck WI, Wright JR. Surfactant protein D stimulates phagocytosis of Pseudomonas aeruginosa by alveolar macrophages. *Am J Respir Cell Mol Biol* 1999;21:576-585.

S16. Katzav S. Vav1: A Dr. Jekyll and Mr. Hyde protein--good for the hematopoietic system, bad for cancer. *Oncotarget*. 2015;6:28731-28742.

S17. Li X, Zhu J, Liu Y, Duan C, Chang R, Zhang C. MicroRNA-331-3p inhibits epithelial-mesenchymal transition by targeting ErbB2 and VAV2 through the Rac1/PAK1/β-catenin axis in non-small-cell lung cancer. *Cancer Sci*. 2019;110:1883-1896.

S18. Sebban S, Farago M, Gashai D, Ilan L, Pikarsky E, Ben-Porath I, et al. Vav1 fine tunes p53 control of apoptosis versus proliferation in breast cancer. *PLoS ONE*. 2013;8:e54321.

S19. Szatmary P, Huang W, Criddle D, Tepikin A, Sutton R. Biology, role and therapeutic potential of circulating histones in acute inflammatory disorders. *J Cell Mol Med*. 2018;22:4617-4629.

S20. Munro S, Hookway ES, Floderer M, Carr SM, Konietzny R, Kessler BM, et al. Linker Histone H1.2 Directs Genome-wide Chromatin Association of the Retinoblastoma Tumor Suppressor Protein and Facilitates Its Function. *Cell Rep*. 2017 13;19:2193-2201.

S21. Zhang Y, Guan L, Zheng Y, Mao L, Li S, Zhao J. Extracellular Histones Promote Pulmonary Fibrosis in Patients With Coal Workers' Pneumoconiosis. *J Occup Environ Med*. 2019;61:89-95.

S22. Zhang Y, Wen Z, Guan L, Jiang P, Gu T, Zhao J, et al. Extracellular histones play an inflammatory role in acid aspiration-induced acute respiratory distress syndrome. *Anesthesiology*. 2015;122:127-139.

S23. Liu L, Yang Y, Liu S, Tao T, Cai J, Wu J, et al. EGF-induced nuclear localization of SHCBP1 activates β-catenin signaling and promotes cancer progression. *Oncogene*. 2019;38:747-764.

S24. Smith MA, Licata T, Lakhani A, Garcia MV, Schildhaus H-U, Vuaroqueaux V, et al. MET-GRB2 Signaling-Associated Complexes Correlate with Oncogenic MET Signaling and Sensitivity to MET Kinase Inhibitors. *Clin Cancer Res*. 2017;23:7084-7096.

S25. Ye X-Y, Xu L, Lu S, Chen Z-W. MiR-516a-5p inhibits the proliferation of non-small cell lung cancer by targeting HIST3H2A. *Int J Immunopathol Pharmacol*. 2019;33:2058738419841481.

S26. Leonard A, Rahman A, Fazal F. Importins α and β signaling mediates endothelial cell inflammation and barrier disruption. *Cell Signal*. 2018;44:103-117.

S27. Nishio S, Ushijima K, Tsuda N, Takemoto S, Kawano K, Yamaguchi T, et al. Cap43/NDRG1/Drg-1 is a molecular target for angiogenesis and a prognostic indicator in cervical adenocarcinoma. *Cancer Lett*. 2008;264:36-43.

S28. Lee M, Hwang Y-S, Yoon J, Sun J, Harned A, Nagashima K, et al. Developmentally regulated GTP-binding protein 1 modulates ciliogenesis via an interaction with Dishevelled. *J Cell Biol*. 2019;218:2659-2676.

S29. Azuma K, Kawahara A, Hattori S, Taira T, Tsurutani J, Watari K, et al. NDRG1/Cap43/Drg-1 may predict tumor angiogenesis and poor outcome in patients with lung cancer. *J Thorac Oncol*. 2012;7:779-789.

S30. Geraghty P, Hardigan A, Foronjy RF. Cigarette smoke activates the proto-oncogene c-src to promote airway inflammation and lung tissue destruction. *Am J Respir Cell Mol Biol*. 2014;50:559-570.

S31. Chung S, Vu S, Filosto S, Goldkorn T. Src regulates cigarette smoke-induced ceramide generation via neutral sphingomyelinase 2 in the airway epithelium. *Am J Respir Cell Mol Biol*. 2015;52:738-748.

S32. Solanki HS, Advani J, Khan AA, Radhakrishnan A, Sahasrabuddhe NA, Pinto SM, et al. Chronic Cigarette Smoke Mediated Global Changes in Lung Mucoepidermoid Cells: A Phosphoproteomic Analysis. *OMICS*. 2017;21:474-487.

S33. Wang W, Ye Y, Li J, Li X, Zhou X, Tan D, et al. Lyn regulates cytotoxicity in respiratory epithelial cells challenged by cigarette smoke extracts. *Curr Mol Med*. 2014;14:663-672.

S34. Sun Q, Fang L, Tang X, Lu S, Tamm M, Stolz D, et al. TGF-β Upregulated Mitochondria Mass through the SMAD2/3→C/EBPβ→PRMT1 Signal Pathway in Primary Human Lung Fibroblasts. *J Immunol*. 2019;202:37-47.

S35. Teng R-J, Du J, Welak S, Guan T, Eis A, Shi Y, et al. Cross talk between NADPH oxidase and autophagy in pulmonary artery endothelial cells with intrauterine persistent pulmonary hypertension. *Am J Physiol Lung Cell Mol Physiol*. 2012;302:L651-L663.

S36. Feng Y, Gross S, Wolf NM, Butenschön VM, Qiu Y, Devraj K, et al. Nucleoside diphosphate kinase B regulates angiogenesis through modulation of vascular endothelial growth factor receptor type 2 and endothelial adherens junction proteins. *Arterioscler Thromb Vasc Biol*. 2014;34:2292-2300.

S37. Gross S, Devraj K, Feng Y, Macas J, Liebner S, Wieland T. Nucleoside diphosphate kinase B regulates angiogenic responses in the endothelium via caveolae formation and c-Src-mediated caveolin-1 phosphorylation. *J Cereb Blood Flow Metab*. 201;37:2471-2484.

S38. Kar A, Saha D, Purohit G, Singh A, Kumar P, Yadav VK, et al. Metastases suppressor NME2 associates with telomere ends and telomerase and reduces telomerase activity within cells. *Nucleic Acids Res*. 2012;40:2554-2565.

S39. Chen H, Liao K, Cui-Zhao L, Qiang-Wen F, Feng-Zeng X, Ping-Wu F, et al. Cigarette smoke extract induces apoptosis of rat alveolar Type II cells via the PLTP/TGF-β1/Smad2 pathway. *Int Immunopharmacol*. 2015;28:707-714.

S40. Liu Y, Liu F, Cao Y, Xu H, Wu Y, Wu S, et al. Shwachman-Diamond Syndrome Protein SBDS Maintains Human Telomeres by Regulating Telomerase Recruitment. Cell Rep. 2018;22:1849-1860.

S41. Nandakumar J, Cech TR. Finding the end: recruitment of telomerase to telomeres. *Nat Rev Mol Cell Biol*. 2013;14:69-82.

S42. Ganapathi KA, Austin KM, Lee C-S, Dias A, Malsch MM, Reed R, et al. The human Shwachman-Diamond syndrome protein, SBDS, associates with ribosomal RNA. *Blood*. 2007;110:1458-1465.

S43. Fan Y, Wang Y, Wang K. Prostaglandin E2 stimulates normal bronchial epithelial cell growth through induction of c-Jun and PDK1, a kinase implicated in oncogenesis. *Respir Res*. 2015;16:149.

S44. Fang R, Cui Q, Sun J, Duan X, Ma X, Wang W, et al. PDK1/Akt/PDE4D axis identified as a target for asthma remedy synergistic with β2 AR agonists by a natural agent arctigenin. *Allergy*. 2015;70:1622-1632.

S45. Sacco F, Humphrey SJ, Cox J, Mischnik M, Schulte A, Klabunde T, et al. Glucose-regulated and drug-perturbed phosphoproteome reveals molecular mechanisms controlling insulin secretion. *Nat Commun*. 2016; 7:13250.

S46. Quotti Tubi L, Canovas Nunes S, Brancalion A, Doriguzzi Breatta E, Manni S, Mandato E, et al. Protein kinase CK2 regulates AKT, NF-κB and STAT3 activation, stem cell viability and proliferation in acute myeloid leukemia. *Leukemia*. 2017;31:292-300.

S47. André-Grégoire G, Dilasser F, Chesné J, Braza F, Magnan A, Loirand G, et al. Targeting of Rac1 prevents bronchoconstriction and airway hyperresponsiveness. *J Allergy Clin Immuno*l. 2018;142:824-33.

S48. Schnoor M, García Ponce A, Vadillo E, Pelayo R, Rossaint J, Zarbock A. Actin dynamics in the regulation of endothelial barrier functions and neutrophil recruitment during endotoxemia and sepsis. *Cell Mol Life Sci*. 2017;74:1985-1997.

S49. Shen H, Sun Y, Zhang S, Jiang J, Dong X, Jia Y, et al. Cigarette smoke-induced alveolar epithelial-mesenchymal transition is mediated by Rac1 activation. *Biochim Biophys Acta*. 2014;1840:1838-1849.

S50. Jiang J-X, Zhang S-J, Shen H-J, Guan Y, Liu Q, Zhao W, et al. Rac1 signaling regulates cigarette smoke-induced inflammation in the lung via the Erk1/2 MAPK and STAT3 pathways. *Biochim Biophys Acta Mol Basis Dis*. 2017;1863:1778-1788.

S51. Das S, Das B. eIF4G-an integrator of mRNA metabolism? *FEMS Yeast Res*. 2016;16: fow087.

S52. Siddiqui N, Sonenberg N. Signalling to eIF4E in cancer. *Biochem Soc Trans*. 2015;43:763-772.

S53. Vaysse C, Philippe C, Martineau Y, Quelen C, Hieblot C, Renaud C, et al. Key contribution of eIF4H-mediated translational control in tumor promotion. *Oncotarget*. 2015;6:39924-39940.

S54. Li D, Ferrari M, Ellis EM. Human aldo-keto reductase AKR7A2 protects against the cytotoxicity and mutagenicity of reactive aldehydes and lowers intracellular reactive oxygen species in hamster V79-4 cells. *Chem Biol Interact*. 2012;195:25-34.

S55. Barnawi J, Tran H, Jersmann H, Pitson S, Roscioli E, Hodge G, et al. Potential Link between the Sphingosine-1-Phosphate (S1P) System and Defective Alveolar Macrophage Phagocytic Function in Chronic Obstructive Pulmonary Disease (COPD). *PLoS ONE*. 2015;10:e0122771.

S56. Nakano T, Goto S, Takaoka Y, Tseng H-P, Fujimura T, Kawamoto S, et al. A novel moonlight function of glyceraldehyde-3-phosphate dehydrogenase (GAPDH) for immunomodulation. *Biofactors*. 2018;44:597-608.

S57. Takaoka Y, Goto S, Nakano T, Tseng H-P, Yang S-M, Kawamoto S, et al. Glyceraldehyde-3-phosphate dehydrogenase (GAPDH) prevents lipopolysaccharide (LPS)-induced, sepsis-related severe acute lung injury in mice. *Sci Rep*. 2014;4:5204.

S58. Collaud S, Tischler V, Atanassoff A, Wiedl T, Komminoth P, Oehlschlegel C, et al. Lung neuroendocrine tumors: correlation of ubiquitinylation and sumoylation with nucleo-cytosolic partitioning of PTEN. *BMC Cancer*. 2015;15:74.

S59. Shi Y, Yuan B, Zhu W, Zhang R, Li L, Hao X, et al. Ube2D3 and Ube2N are essential for RIG-I-mediated MAVS aggregation in antiviral innate immunity. *Nat Commun*. 2017;8:15138.

S60. Gemoll T, Miroll E, Klein O, Lischka A, Eravci M, Thorns C, et al. Spatial UBE2N protein expression indicates genomic instability in colorectal cancers. *BMC Cancer*. 2019;19:710.

S61. Min T, Bodas M, Mazur S, Vij N. Critical role of proteostasis-imbalance in pathogenesis of COPD and severe emphysema. *J Mol Med*. 2011;89:577-593.

S62. Parihar MS, Parihar A, Fujita M, Hashimoto M, Ghafourifar P. Alpha-synuclein overexpression and aggregation exacerbates impairment of mitochondrial functions by augmenting oxidative stress in human neuroblastoma cells. *Int J Biochem Cell Biol*. 2009;41:2015-2024.

S63. Ottolini D, Calí T, Szabò I, Brini M. Alpha-synuclein at the intracellular and the extracellular side: functional and dysfunctional implications. *Biol Chem*. 2017;398:77-100.

S64. Xue Q, Lv L, Wan C, Chen B, Li M, Ni T, et al. Expression and clinical role of small glutamine-rich tetratricopeptide repeat (TPR)-containing protein alpha (SGTA) as a novel cell cycle protein in NSCLC. *J Cancer Res Clin Oncol*. 2013;139:1539-1549.

S65. Abonyo BO, Wang P, Narasaraju TA, Rowan WH, McMillan DH, Zimmerman U-J, et al. Characterization of alpha-soluble N-ethylmaleimide-sensitive fusion attachment protein in alveolar type II cells: implications in lung surfactant secretion. *Am J Respir Cell Mol Biol*. 2003;29(3 Pt 1):273-282.

S66. Lechuga S, Naydenov NG, Feygin A, Jimenez AJ, Ivanov AI. A vesicle trafficking protein αSNAP regulates Paneth cell differentiation in vivo. *Biochem Biophys Res Commun*. 2017;486:951-957.

S67. Naydenov NG, Brown B, Harris G, Dohn MR, Morales VM, Baranwal S, et al. A membrane fusion protein αSNAP is a novel regulator of epithelial apical junctions. *PLoS ONE*. 2012;7:e34320.

S68. Naydenov NG, Feygin A, Wang L, Ivanov AI. N-ethylmaleimide-sensitive factor attachment protein α (αSNAP) regulates matrix adhesion and integrin processing in human epithelial cells. *J Biol Chem*. 2014;289:2424-2439.

S69. Şahin F, Koşar AF, Aslan AF, Yiğitbaş B, Uslu B. Serum Biomarkers in Patients with Stable and Acute Exacerbation of Chronic Obstructive Pulmonary Disease: A Comparative Study. *J Med Biochem*. 2019;38:503-511.

S70. Xu L, Chen Y, Xie Z, He Q, Chen S, Wang W, et al. High hemoglobin is associated with increased in-hospital death in patients with chronic obstructive pulmonary disease and chronic kidney disease: a retrospective multicenter population-based study. *BMC Pulm Med*. 2019;19:174.

S71. Bernier SG, Taghizadeh N, Thompson CD, Westlin WF, Hannig G. Methionine aminopeptidases type I and type II are essential to control cell proliferation. *J Cell Biochem*. 2005;95:1191-1203.

S72. Shimizu H, Yamagishi S, Chiba H, Ghazizadeh M. Methionine Aminopeptidase 2 as a Potential Therapeutic Target for Human Non-Small-Cell Lung Cancers. *Adv Clin Exp Med*. 2016;25:117-128.

S73. Rother M, Gonzalez E, Teixeira da Costa AR, Wask L, Gravenstein I, Pardo M, et al. Combined Human Genome-wide RNAi and Metabolite Analyses Identify IMPDH as a Host-Directed Target against Chlamydia Infection. *Cell Host Microbe*. 2018;23:661-671.e8.

S74. Huang F, Ni M, Chalishazar MD, Huffman KE, Kim J, Cai L, et al. Inosine Monophosphate Dehydrogenase Dependence in a Subset of Small Cell Lung Cancers. *Cell Metab*. 2018;28:369-382.e5.

S75. Porter SE, Dwyer-Nield LD, Malkinson AM. Regulation of lung epithelial cell morphology by cAMP-dependent protein kinase type I isozyme. *Am J Physiol Lung Cell Mol Physiol*. 2001;280:L1282-1289.

S76. Vanessa KHQ, Julia MG, Wenwei L, Michelle ALT, Zarina ZRS, Lina LHK, et al. Absence of Annexin A1 impairs host adaptive immunity against Mycobacterium tuberculosis in vivo. *Immunobiology*. 2015;220:614-23.

S77. Lai T, Li Y, Mai Z, Wen X, Lv Y, Xie Z, et al. Annexin A1 is elevated in patients with COPD and affects lung fibroblast function. *Int J Chron Obstruct Pulmon Dis*. 2018;13:473-486.

S78. Salama MF, Liu M, Clarke CJ, Espaillat MP, Haley JD, Jin T, et al. PKCα is required for Akt-mTORC1 activation in non-small cell lung carcinoma (NSCLC) with EGFR mutation. *Oncogene*. 2019 Nov;38(48):7311-7328.

S79. Artamonov MV, Sonkusare SK, Good ME, Momotani K, Eto M, Isakson BE, et al. RSK2 contributes to myogenic vasoconstriction of resistance arteries by activating smooth muscle myosin and the Na+/H+ exchanger. *Sci Signal*. 2018 30;11:eaar3924.

S80. Passariello CL, Martinez EC, Thakur H, Cesareo M, Li J, Kapiloff MS. RSK3 is required for concentric myocyte hypertrophy in an activated Raf1 model for Noonan syndrome. *J Mol Cell Cardiol*. 2016;93:98-105.

S81. Rispal J, Baron L, Beaulieu J-F, Chevillard-Briet M, Trouche D, Escaffit F. The H2A.Z histone variant integrates Wnt signaling in intestinal epithelial homeostasis. *Nat Commun*. 2019 23;10:1827.

S82. Domaschenz R, Kurscheid S, Nekrasov M, Han S, Tremethick DJ. The Histone Variant H2A.Z Is a Master Regulator of the Epithelial-Mesenchymal Transition. *Cell Rep*. 2017;21:943-952.

S83. Xuan Y, Wang J, Ban L, Lu J-J, Yi C, Li Z, et al. hnRNPA2/B1 activates cyclooxygenase-2 and promotes tumor growth in human lung cancers. *Mol Oncol*. 2016;10:610-624.

S84. Wang T-H, Chen C-C, Hsiao Y-C, Lin Y-H, Pi W-C, Huang P-R, et al. Heterogeneous Nuclear Ribonucleoproteins A1 and A2 Function in Telomerase-Dependent Maintenance of Telomeres. *Cancers* (Basel). 2019;11:334.

S85. Liu X, Zhou Y, Lou Y, Zhong H. Knockdown of HNRNPA1 inhibits lung adenocarcinoma cell proliferation through cell cycle arrest at G0/G1 phase. *Gene*. 2016;576(2 Pt 2):791-797.

S86. Hung P-F, Hong T-M, Chang C-C, Hung C-L, Hsu Y-L, Chang Y-L, et al. Hypoxia-induced Slug SUMOylation enhances lung cancer metastasis. *J Exp Clin Cancer Res*. 2019;38:5.

S87. Li J, Yue G, Ma W, Zhang A, Zou J, Cai Y, et al. Ufm1-Specific Ligase Ufl1 Regulates Endoplasmic Reticulum Homeostasis and Protects Against Heart Failure. *Circ Heart Fail*. 2018;11:e004917.

S88. Li Y-Y, Zhang G-Y, He J-P, Zhang D-D, Kong X-X, Yuan H-M, et al. Ufm1 inhibits LPS-induced endothelial cell inflammatory responses through the NF-κB signaling pathway. *Int J Mol Med*. 2017;39:1119-1126.

S89. Zhang L, Yang Y, Chai L, Bu H, Yang Y, Huang H, et al. FRK plays an oncogenic role in non-small cell lung cancer by enhancing the stemness phenotype via induction of metabolic reprogramming. *Int J Cancer*. 2020;146:208-222.

S90. Suresh K, Servinsky L, Reyes J, Undem C, Zaldumbide J, Rentsendorj O, et al. CD36 mediates H2O2-induced calcium influx in lung microvascular endothelial cells. *Am J Physiol Lung Cell Mol Physiol*. 2017;312:L143-L153.

S91. Cekan P, Hasegawa K, Pan Y, Tubman E, Odde D, Chen J-Q, et al. RCC1-dependent activation of Ran accelerates cell cycle and DNA repair, inhibiting DNA damage-induced cell senescence. *Mol Biol Cell*. 2016;27:1346-1357.

S92. Ning J, Liu W, Zhang J, Lang Y, Xu S. Ran GTPase induces EMT and enhances invasion in non-small cell lung cancer cells through activation of PI3K-AKT pathway. *Oncol Res*. 2013;21:67-72.

S93. Dai J, Zhou Q, Chen J, Rexius-Hall ML, Rehman J, Zhou G. Alpha-enolase regulates the malignant phenotype of pulmonary artery smooth muscle cells via the AMPK-Akt pathway. *Nat Commun*. 2018;9:3850.

S94. Rangarajan S, Bone NB, Zmijewska AA, Jiang S, Park DW, Bernard K, et al. Metformin reverses established lung fibrosis in a bleomycin model. *Nat Med*. 2018;24:1121-1127.

S95. Cole AJ, Clifton-Bligh R, Marsh DJ. Histone H2B monoubiquitination: roles to play in human malignancy. *Endocr Relat Cancer*. 2015;22:T19-T33.

S96. Wang Y, Zhang H, Kang Q, Liu J, Weng H, Li W, et al. Protein 4.1N is required for the formation of the lateral membrane domain in human bronchial epithelial cells. *Biochim Biophys Acta Biomembr*. 2018;1860:1143-1151.

S97. Feng G, Guo K, Yan Q, Ye Y, Shen M, Ruan S, et al. Expression of Protein 4.1 Family in Breast Cancer: Database Mining for 4.1 Family Members in Malignancies. *Med Sci Monit*. 2019;25:3374-3389.

S98. Albano GD, Bonanno A, Moscato M, Anzalone G, Di Sano C, Riccobono L, et al. Crosstalk between mAChRM3 and β2AR, via acetylcholine PI3/PKC/PBEP1/Raf-1 MEK1/2/ERK1/2 pathway activation, in human bronchial epithelial cells after long-term cigarette smoke exposure. *Life Sci*. 2018;192:99-109.

S99. Denfeld QE, Mudd JO, Hasan W, Gelow JM, Hiatt SO, Winters-Stone K, et al. Exploring the relationship between β-adrenergic receptor kinase-1 and physical symptoms in heart failure. *Heart Lung*. 2018;47:281-284.

S100. Morita M, Sato T, Nomura M, Sakamoto Y, Inoue Y, Tanaka R, et al. PKM1 Confers Metabolic Advantages and Promotes Cell-Autonomous Tumor Cell Growth. *Cancer Cell.* 2018 12;33:355-367.

S101. Lim GE, Piske M, Johnson JD. 14-3-3 proteins are essential signalling hubs for beta cell survival. *Diabetologia*. 2013;56:825-837.

S102. Hermeking H, Benzinger A. 14-3-3 proteins in cell cycle regulation. *Semin Cancer Biol*. 2006;16:183-192.

S103. Wilker E, Yaffe MB. 14-3-3 Proteins--a focus on cancer and human disease. *J Mol Cell Cardiol*. 2004;37:633-642.

S104. Li M, Lu H, Liu X, Meng Q, Zhao Y, Chen X, et al. Overexpression of 14-3-3ζ in lung tissue predicts an improved outcome in patients with lung adenocarcinoma. *Oncol Lett*. 2018;16:1051-1058.

S105. Huang S, Zheng B, Jin X, Yu Q, Zhang X, Sun X, et al. Blockade of Cyclophilin D Attenuates Oxidative Stress-Induced Cell Death in Human Dental Pulp Cells. *Oxid Med Cell Longev*. 2019;2019:1729013.

S106. Elrod JW, Wong R, Mishra S, Vagnozzi RJ, Sakthievel B, Goonasekera SA, et al. Cyclophilin D controls mitochondrial pore-dependent Ca(2+) exchange, metabolic flexibility, and propensity for heart failure in mice. *J Clin Invest*. 2010;120:3680-3687.

S107. Baines CP, Kaiser RA, Purcell NH, Blair NS, Osinska H, Hambleton MA, et al. Loss of cyclophilin D reveals a critical role for mitochondrial permeability transition in cell death. *Nature*. 2005;434:658-662.

S108. Lin R, Elf S, Shan C, Kang H-B, Ji Q, Zhou L, et al. 6-Phosphogluconate dehydrogenase links oxidative PPP, lipogenesis and tumour growth by inhibiting LKB1-AMPK signalling. *Nat Cell Biol*. 2015;17:1484-1496.

S109. Ma Y, Yu C, Mohamed EM, Shao H, Wang L, Sundaresan G, et al. A causal link from ALK to hexokinase II overexpression and hyperactive glycolysis in EML4-ALK-positive lung cancer. *Oncogene*. 2016;35:6132-6142.

S110. Wang W, Liu Z, Zhao L, Sun J, He Q, Yan W, et al. Hexokinase 2 enhances the metastatic potential of tongue squamous cell carcinoma via the SOD2-H2O2 pathway. *Oncotarget*. 2017;8:3344-3354.

S111. Jang MK, Kim S-H, Lee K-Y, Kim T-B, Moon KA, Park CS, et al. The tyrosine phosphatase, SHP-1, is involved in bronchial mucin production during oxidative stress. *Biochem Biophys Res Commun*. 2010;393:137-143.

S112. Yuan T, Ma H, Du Z, Xiong X, Gao H, Fang Z, et al. Shp1 positively regulates EGFR signaling by controlling EGFR protein expression in mammary epithelial cells. *Biochem Biophys Res Commun*. 2017;488:439-444.

S113. Amand M, Erpicum C, Gilles C, Noël A, Rahmouni S. Functional Analysis of Dual-Specificity Protein Phosphatases in Angiogenesis. *Methods Mol Biol*. 2016;1447:331-349.

S114. Amand M, Erpicum C, Bajou K, Cerignoli F, Blacher S, Martin M, et al. DUSP3/VHR is a pro-angiogenic atypical dual-specificity phosphatase. *Mol Cancer*. 2014;13:108.

S115. Bae S-Y, Byun S, Bae SH, Min DS, Woo HA, Lee K. TPT1 (tumor protein, translationally-controlled 1) negatively regulates autophagy through the BECN1 interactome and an MTORC1-mediated pathway. *Autophagy*.2017;13:820-833.

S116. Ferrer E, Dunmore BJ, Hassan D, Ormiston ML, Moore S, Deighton J, et al. A Potential Role for Exosomal Translationally Controlled Tumor Protein Export in Vascular Remodeling in Pulmonary Arterial Hypertension. *Am J Respir Cell Mol Biol*. 2018;59:467-478.

S117. Zhang J, de Toledo SM, Pandey BN, Guo G, Pain D, Li H, et al. Role of the translationally controlled tumor protein in DNA damage sensing and repair. *Proc Natl Acad Sci USA*. 2012;109:E926-E933.

S118. Zhang X, Xiao Z, Li C, Xiao Z, Yang F, Li D, et al. Triosephosphate isomerase and peroxiredoxin 6, two novel serum markers for human lung squamous cell carcinoma. *Cancer Sci*. 2009;100:2396-2401.

S119. Hipkiss AR. Energy metabolism and ageing regulation: metabolically driven deamidation of triosephosphate isomerase may contribute to proteostatic dysfunction. *Ageing Res Rev*. 2011;10:498-502.

S120. Tsurumi Y, Hamada Y, Katoh Y, Nakayama K. Interactions of the dynein-2 intermediate chain WDR34 with the light chains are required for ciliary retrograde protein trafficking. *Mol Biol Cell*. 2019;30:658-670.

S121. Sørensen BS, Horsman MR, Vorum H, Honoré B, Overgaard J, Alsner J. Proteins upregulated by mild and severe hypoxia in squamous cell carcinomas in vitro identified by proteomics. *Radiother Oncol*. 2009;92:443-449.

S122. Cai Y, Zhu G, Liu S, Pan Z, Quintero M, Poole CJ, et al. Indispensable role of the Ubiquitin-fold modifier 1-specific E3 ligase in maintaining intestinal homeostasis and controlling gut inflammation. *Cell Discov*. 2019;5:7.

S123. Srikanth CV, Verma S. Sumoylation as an Integral Mechanism in Bacterial Infection and Disease Progression. *Adv Exp Med Biol*. 2017;963:389-408.

S124. Yu M, Wang H, Liu Z, Lu Y, Yu D, Li D, et al. Ebp1 regulates myogenic differentiation of myoblast cells via SMAD2/3 signaling pathway. *Dev Growth Differ*. 2017;59:540-551.

S125. Miao X, Tang Q, Miao X, Wu Y, Qian J, Zhao W, et al. ErbB3 binding protein 1 (EBP1) participates in the regulation of intestinal inflammation via mediating Akt signaling pathway. *Mol Immunol*. 2015;67(2 Pt B):540-51.

S126. Chen C-H, Chuang S-M, Yang M-F, Liao J-W, Yu S-L, Chen JJW. A novel function of YWHAZ/β-catenin axis in promoting epithelial-mesenchymal transition and lung cancer metastasis. *Mol Cancer Res*. 2012;10:1319-1331.

S127. Stanford SM, Aleshin AE, Zhang V, Ardecky RJ, Hedrick MP, Zou J, et al. Diabetes reversal by inhibition of the low-molecular-weight tyrosine phosphatase. *Nat Chem Biol*. 2017;13:624-632.

S128. Lori G, Gamberi T, Paoli P, Caselli A, Pranzini E, Marzocchini R, et al. LMW-PTP modulates glucose metabolism in cancer cells. *Biochim Biophys Acta Gen Subj*. 2018;1862(12):2533-2544.

S129. Xu IM-J, Lai RK-H, Lin S-H, Tse AP-W, Chiu DK-C, Koh H-Y, et al. Transketolase counteracts oxidative stress to drive cancer development. *Proc Natl Acad Sci USA*. 2016;113:E725-E734.

S130. Heller S, Maurer GD, Wanka C, Hofmann U, Luger A-L, Bruns I, et al. Gene Suppression of Transketolase-Like Protein 1 (TKTL1) Sensitizes Glioma Cells to Hypoxia and Ionizing Radiation. *Int J Mol Sci*. 2018;19:2168.

S131. Li H, Niu H, Peng Y, Wang J, He P. Ubc9 promotes invasion and metastasis of lung cancer cells. *Oncol Rep*. 2013;29:1588-1594.

S132. Rulifson IC, Collins P, Miao L, Nojima D, Lee KJ, Hardy M, et al. In Vitro and in Vivo Analyses Reveal Profound Effects of Fibroblast Growth Factor 16 as a Metabolic Regulator. *J Biol Chem*. 2017;292:1951-1969.

S133. Duan Y, Wang Y, Li X, Mo J, Guo X, Li C, et al. Fibroblast growth factor 16 stimulates proliferation but blocks differentiation of rat stem Leydig cells during regeneration. *J Cell Mol Med*. 2019;23:2632-2264.

S134. Wegner KA, Mehta V, Johansson JA, Mueller BR, Keil KP, Abler LL, et al. Edar is a downstream target of beta-catenin and drives collagen accumulation in the mouse prostate. *Biol Open*. 2019;18:8: bio037945.

S135. Kowalczyk-Quintas C, Schneider P. Ectodysplasin A (EDA) - EDA receptor signalling and its pharmacological modulation. *Cytokine Growth Factor Rev*. 2014;25:195-203.

S136. Gutbier B, Schönrock SM, Ehrler C, Haberberger R, Dietert K, Gruber AD, et al. Sphingosine Kinase 1 Regulates Inflammation and Contributes to Acute Lung Injury in Pneumococcal Pneumonia via the Sphingosine-1-Phosphate Receptor 2. *Crit Care Med*. 2018;46:e258-e267.

S137. Peng C, Zhao H, Song Y, Chen W, Wang X, Liu X, et al. SHCBP1 promotes synovial sarcoma cell metastasis via targeting TGF-β1/Smad signaling pathway and is associated with poor prognosis. *J Exp Clin Cancer Res*. 2017 11;36:141.

S138. North ML, Khanna N, Marsden PA, Grasemann H, Scott JA. Functionally important role for arginase 1 in the airway hyperresponsiveness of asthma. *Am J Physiol Lung Cell Mol Physiol*. 2009;296:L911-L920.

S139. Zhang H, Liu J, Qu D, Wang L, Wong CM, Lau C-W, et al. Serum exosomes mediate delivery of arginase 1 as a novel mechanism for endothelial dysfunction in diabetes. *Proc Natl Acad Sci USA*. 2018 17;115(29):E6927-E6936.

S140. Zimmermann N, Rothenberg ME. The arginine-arginase balance in asthma and lung inflammation. *Eur J Pharmacol*. 2006;533(1–3):253-262.

S141. Beziere N, Fuchs K, Maurer A, Reischl G, Brück J, Ghoreschi K, et al. Imaging fibrosis in inflammatory diseases: targeting the exposed extracellular matrix. *Theranostics*. 2019;9:2868-2881.

S142. Jooss NJ, De Simone I, Provenzale I, Fernández DI, Brouns SLN, Farndale RW, et al. Role of Platelet Glycoprotein VI and Tyrosine Kinase Syk in Thrombus Formation on Collagen-Like Surfaces. *Int J Mol Sci*. 2019;20:2788.

S143. Zhang M, Tang J, Yin J, Wang X, Feng X, Yang X, et al. The clinical implication of serum cyclophilin A in patients with chronic obstructive pulmonary disease. *Int J Chron Obstruct Pulmon Dis*. 2018;13:357-363.

S144. Dawar FU, Wu J, Zhao L, Khattak MNK, Mei J, Lin L. Updates in understanding the role of cyclophilin A in leukocyte chemotaxis. *J Leukoc Biol*. 2017;101:823-826.

S145. Nagaprashantha LD, Vatsyayan R, Lelsani PCR, Awasthi S, Singhal SS. The sensors and regulators of cell-matrix surveillance in anoikis resistance of tumors. *Int J Cancer*. 2011;128:743-752.

S146. Du C, Lu J, Zhou L, Wu B, Zhou F, Gu L, et al. MAPK/FoxA2-mediated cigarette smoke-induced squamous metaplasia of bronchial epithelial cells. *Int J Chron Obstruct Pulmon Dis*. 2017;12:3341-3351.

S147. Yu M, Liu X, Wu H, Ni W, Chen S, Xu Y. Small interfering RNA against ERK1/2 attenuates cigarette smoke-induced pulmonary vascular remodeling. *Exp Ther Med*. 2017;14:4671-4680.

S148. Kim J-E, Kim Y-J, Lee D-S, Kim JY, Ko A-R, Hyun H-W, et al. PLPP/CIN regulates bidirectional synaptic plasticity via GluN2A interaction with postsynaptic proteins. *Sci Rep*. 2016;6:26576.

S149. Delorme-Walker V, Seo J-Y, Gohla A, Fowler B, Bohl B, DerMardirossian C. Chronophin coordinates cell leading edge dynamics by controlling active cofilin levels. *Proc Natl Acad Sci USA*. 2015;112:E5150-E5159.

S150. Cheal Yoo J, Park N, Lee B, Nashed A, Lee Y-S, Hwan Kim T, et al. 14-3-3γ regulates Copine1-mediated neuronal differentiation in HiB5 hippocampal progenitor cells. *Exp Cell Res*. 2017;356:85-92.

S151. Liu S, Tang H, Zhu J, Ding H, Zeng Y, Du W, et al. High expression of Copine 1 promotes cell growth and metastasis in human lung adenocarcinoma. *Int J Oncol*. 2018;53:2369-2378.

S152. Tian Y, Zeng T, Tan L, et al. Clinical significance of BPI-ANCA detecting in COPD patients with Pseudomonas aeruginosa colonization. *J Clin Lab Anal*. 2019;33:e22908.

S153. Holweg A, Schnare M, Gessner A. The bactericidal/permeability-increasing protein (BPI) in the innate defense of the lower airways. *Biochem Soc Trans*. 2011;39:1045-1050.

S154. Bülow S, Zeller L, Werner M, Toelge M, Holzinger J, Entzian C, et al. Bactericidal/Permeability-Increasing Protein Is an Enhancer of Bacterial Lipoprotein Recognition. *Front Immunol*. 2018;9:2768.

S155. Xu Y, Zhu J, Hu X, Wang C, Lu D, Gong C, et al. CLIC1 Inhibition Attenuates Vascular Inflammation, Oxidative Stress, and Endothelial Injury. *PLoS ONE*. 2016;11:e0166790.

S156. Ulmasov B, Bruno J, Oshima K, Cheng Y-W, Holly SP, Parise LV, et al. CLIC1 null mice demonstrate a role for CLIC1 in macrophage superoxide production and tissue injury. *Physiol Rep*. 2017;5: e13169.

S157. Vij N, Chandramani-Shivalingappa P, Van Westphal C, Hole R, Bodas M. Cigarette smoke-induced autophagy impairment accelerates lung aging, COPD-emphysema exacerbations and pathogenesis. *Am J Physiol Cell Physiol*. 2018;314:C73-C87.

S158. Lubas M, Harder LM, Kumsta C, Tiessen I, Hansen M, Andersen JS, et al. eIF5A is required for autophagy by mediating ATG3 translation. *EMBO Rep*. 2018;19: e46072.

S159. Seko Y, Fujimura T, Yao T, Taka H, Mineki R, Okumura K, et al. Secreted tyrosine sulfated-eIF5A mediates oxidative stress-induced apoptosis. *Sci Rep*. 2015;5:13737.

S160. Wang Z, Jiang J, Qin T, Xiao Y, Han L. EIF5A regulates proliferation and chemoresistance in pancreatic cancer through the sHH signalling pathway. *J Cell Mol Med*. 2019;23:2678-2688.

S161. Koplin A, Preissler S, Ilina Y, Koch M, Scior A, Erhardt M, et al. A dual function for chaperones SSB-RAC and the NAC nascent polypeptide-associated complex on ribosomes. *J Cell Biol*. 2010;189:57-68.

S162. Ponce-Rojas JC, Avendaño-Monsalve MC, Yañez-Falcón AR, Jaimes-Miranda F, Garay E, Torres-Quiroz F, et al. αβ'-NAC cooperates with Sam37 to mediate early stages of mitochondrial protein import. *FASEB J*. 2017;284:814-830.

S163. Zuo W-L, Yang J, Gomi K, Chao I, Crystal RG, Shaykhiev R. EGF-Amphiregulin Interplay in Airway Stem/Progenitor Cells Links the Pathogenesis of Smoking-Induced Lesions in the Human Airway Epithelium. *Stem Cells*. 2017;35:824-837.

S164. Wang J, Zhu M, Wang L, Chen C, Song Y. Amphiregulin potentiates airway inflammation and mucus hypersecretion induced by urban particulate matter via the EGFR-PI3Kα-AKT/ERK pathway. *Cell Signal*. 2019;53:122-131.

S165. Ogata-Suetsugu S, Yanagihara T, Hamada N, Ikeda-Harada C, Yokoyama T, Suzuki K, et al. Amphiregulin suppresses epithelial cell apoptosis in lipopolysaccharide-induced lung injury in mice. *Biochem Biophys Res Commun*. 2017;484:422-428.

S166. Bonora M, Wieckowski MR, Chinopoulos C, Kepp O, Kroemer G, Galluzzi L, et al. Molecular mechanisms of cell death: central implication of ATP synthase in mitochondrial permeability transition. *Oncogene*. 34:1475-1486.

S167. Liang Y, Shen Y, Kuang L, Zhou G, Zhang L, Zhong X, et al. Cigarette smoke exposure promotes differentiation of CD4+ T cells toward Th17 cells by CD40-CD40L costimulatory pathway in mice. *Int J Chron Obstruct Pulmon Dis.* 2018;13:959-968.

S168. Kuang L-J, Deng T-T, Wang Q, Qiu S-L, Liang Y, He Z-Y, et al. Dendritic cells induce Tc1 cell differentiation via the CD40/CD40L pathway in mice after exposure to cigarette smoke. *Am J Physiol Lung Cell Mol Physiol*. 2016;311:L581-L589.

S169. Deng TT, Zhong XN, Wang Q, Kuang LJ, Qiu SL, Liang Y, et al. Effect of CD40 on Foxp3(+) Treg cell in the lung of cigarette smoke exposure mice]. *Zhonghua Yi Xue Za Zhi*. 2016;96:1139-1143.

S170. Pyo J-H, Jeon H-J, Park J-S, Lee J-S, Chung H-Y, Yoo M-A. Drosophila PEBP1 inhibits intestinal stem cell aging via suppression of ERK pathway. *Oncotarget*. 2018;9:17980-17993.

S171. Zhao J, O'Donnell VB, Balzar S, St Croix CM, Trudeau JB, Wenzel SE. 15-Lipoxygenase 1 interacts with phosphatidylethanolamine-binding protein to regulate MAPK signaling in human airway epithelial cells. *Proc Natl Acad Sci USA*. 2011;108:14246-14251.

S172. Wenzel SE, Tyurina YY, Zhao J, St Croix CM, Dar HH, Mao G, et al. PEBP1 Wardens Ferroptosis by Enabling Lipoxygenase Generation of Lipid Death Signals. *Cell*. 2017;171:628-641.

S173. Valenti P, Frioni A, Rossi A, Ranucci S, De Fino I, Cutone A, et al. Aerosolized bovine lactoferrin reduces neutrophils and pro-inflammatory cytokines in mouse models of Pseudomonas aeruginosa lung infections. *Biochem Cell Biol*. 2017;95:41-47.

S174. Almansa R, Socias L, Sanchez-Garcia M, Martín-Loeches I, del Olmo M, Andaluz-Ojeda D, et al. Critical COPD respiratory illness is linked to increased transcriptomic activity of neutrophil proteases genes. *BMC Res Notes*. 2012;5:401.

S175. Takahashi K, Pavlidis S, Ng Kee Kwong F, Hoda U, Rossios C, Sun K, et al. Sputum proteomics and airway cell transcripts of current and ex-smokers with severe asthma in U-BIOPRED: an exploratory analysis. *Eur Respir J*. 2018;51.

S176. Ma M, Kumar S, Purushothaman L, Babst M, Ungermann C, Chi RJ, et al. Lipid trafficking by yeast Snx4 family SNX-BAR proteins promotes autophagy and vacuole membrane fusion. *Mol Biol Cell*. 2018;29:2190-2200.

S177. Mallia P, Webber J, Gill SK, Trujillo-Torralbo M-B, Calderazzo MA, Finney L, et al. Role of airway glucose in bacterial infections in patients with chronic obstructive pulmonary disease. *J Allergy Clin Immunol*. 2018;142:815-823.

S178. Hao W, Li M, Zhang Y, Zhang C, Xue Y. Expressions of MMP-12, TIMP-4, and Neutrophil Elastase in PBMCs and Exhaled Breath Condensate in Patients with COPD and Their Relationships with Disease Severity and Acute Exacerbations. *J Immunol Res*. 2019;2019:7142438.

S179. Hou H-H, Wang H-C, Cheng S-L, Chen Y-F, Lu K-Z, Yu C-J. MMP-12 activates protease-activated receptor-1, upregulates placenta growth factor, and leads to pulmonary emphysema. *Am J Physiol Lung Cell Mol Physiol*. 2018;315:L432-442.

S180. Wang J, Chen L, Chen B, Meliton A, Liu SQ, Shi Y, et al. Chronic Activation of the Renin-Angiotensin System Induces Lung Fibrosis. *Sci Rep*. 2015;5:p61.

S181. Chiang H-Y, Chu P-H, Lee T-H. MFG-E8 mediates arterial aging by promoting the proinflammatory phenotype of vascular smooth muscle cells. *J Biomed Sci*. 2019;26:61.

S182. Hirano Y, Yang W-L, Aziz M, Zhang F, Sherry B, Wang P. MFG-E8-derived peptide attenuates adhesion and migration of immune cells to endothelial cells. *J Leukoc Biol.* 2017;101:1201-1209.

S183. Khalifeh-Soltani A, Gupta D, Ha A, Podolsky MJ, Datta R, Atabai K. The Mfge8-α8β1-PTEN pathway regulates airway smooth muscle contraction in allergic inflammation. *FASEB J*. 2018;fj201800109R.

S184. Ricklin D, Reis ES, Mastellos DC, Gros P, Lambris JD. Complement component C3 - The “Swiss Army Knife” of innate immunity and host defense. *Immunol Rev.* 2016;274:33-58.

S185. Misa K, Tanino Y, Wang X, Nikaido T, Kikuchi M, Sato Y, et al. Involvement of midkine in the development of pulmonary fibrosis. *Physiol Rep.* 2017;5: e13383.

S186. Ak G, Tada Y, Shimada H, Metintas S, Ito M, Hiroshima K, et al. Midkine is a potential novel marker for malignant mesothelioma with different prognostic and diagnostic values from mesothelin. *BMC Cancer.* 2017;17:212.

S187. Linge HM, Andersson C, Nordin SL, Olin AI, Petersson A-C, Mörgelin M, et al. Midkine is expressed and differentially processed during chronic obstructive pulmonary disease exacerbations and ventilator-associated pneumonia associated with Staphylococcus aureus infection. *Mol Med.* 2013;19:314-323.

Supporting information

S4 Table: Significant correlations between differentially regulated proteins and the incidence of exacerbations and emphysema in patients with COPD at visit 1.

Biological Hallmarks	Target	Exacerbations (Y/N)		Emphysema (Y/N)	
		OR (2.5-97.5%)	FDR	OR (2.5-97.5%)	FDR
Cell fate, Remodeling and Repair	PRKCA [a]	0.57 (0.41-0.79)	0.016	-	-
	PRKACA [a]	-	-	0.62 (0.41-0.90)	0.0200
	SHC1	-	-	0.33 (0.17-0.58)	0.0026
	eIF-5A-1	-	-	0.43 (0.18-0.60)	0.0026
	TCTP	0.57 (0.36-0.87)	0.0476	0.35 (0.20-0.58)	0.0026
	PA2G4	-	-	0.40 (0.24-0.64)	0.0026
	**14-3-3 protein β/α [a]**	0.50 (0.30-0.79)	0.0281	0.41 (0.24-0.68)	0.0032
	14-3-3 protein ζ/δ [a]	0.45 (0.28-0.70)	0.0160	0.42 (0.26-0.68)	0.0031
	BAD [a]	-	-	0.45 (0.27-0.70)	0.0031
	RPS6KA3 [a]	0.61 (0.40-0.89)	0.048	0.46 (0.29-0.71)	0.0031
	**AREG**	-	-	0.46 (0.27-0.75)	0.0065
	METAP1 [a]	-	-	0.50 (0.32-0.76)	0.0044
	**FGF16**	-	-	0.52 (0.31-0.81)	0.0115
	RAC1 [a]	0.58 (0.40-0.82)	0.022	0.52 (0.35-0.76)	0.0033
	SBDS	0.66 (0.48-0.92)	0.048	0.53 (0.36-0.76)	0.0031
	hnRNP A2/B1	-	-	0.53 (0.36-0.79)	0.0060
	NSF1C	-	-	0.55 (0.39-0.77)	0.0031
	PKC-A [a]	-	-	0.55 (0.38-0.78)	0.0042
	SMAD2 [a]	0.67 (0.49-0.92)	0.048	0.58 (0.40-0.81)	0.0059
	**eIF-4H**	0.63 (0.45-0.86)	0.028	0.59 (0.41-0.82)	0.0061
	DRG-1	-	-	0.51 (0.35-0.72)	0.0026
	DLRB1	-	-	0.61 (0.39-0.91)	0.025
	PLPP	-	-	0.36 (0.20-0.62)	0.0026
	ARGI1	0.62 (0.43-0.88)	0.047	-	-
	IMB1 [a]	-	-	0.60 (0.43-0.80)	0.003
	ARGI1 [a]	0.62 (0.43-0.88)	0.047	-	-
	41	-	-	0.62 (0.44-0.86)	0.010
	GRB2 adapter protein	0.72 (0.55-0.93)	0.017	0.64 (0.49-0.85)	0.004
	**Tropomyosin 4**	0.78 (0.64-0.96)	0.024	0.71 (0.57-0.89)	0.004
	Sphingosine kinase 1	0.66 (0.47-0.91)	0.048	0.60 (0.41-0.85)	0.008
	Hemoglobin	-	-	0.72 (0.54-0.93)	0.027
	CSK	-	-	0.64 (0.47-0.86)	0.006
	FYN [a]	-	-	0.74 (0.56-0.97)	0.042
	ERK-1 [a]	0.50 (0.29-0.84)	0.0476	0.41 (0.22-0.72)	0.0062
	MK01/ERK-2	-	-	0.45 (0.26-0.74)	0.0061
	PKC-B-II	0.59 (0.43-0.80)	0.0158	0.55 (0.40-0.76)	0.0026
	Prostatic binding protein	-	-	0.41 (0.24-0.68)	0.0032
	Caspase-3	0.66 (0.47-0.91)	0.048	0.52 (0.35-0.74)	0.0030
	PDPK1 [a]	-	-	0.61 (0.43-0.87)	0.011
	LYN [a]	0.65 (0.47-0.87)	0.029	0.65 (0.47-0.89)	0.013
	LYNB [a]	0.65 (0.48-0.87)	0.028	0.68 (0.50-0.91)	0.016
	CK2-A1:B	-	-	0.56 (0.40-8.80)	0.0164
	NDP kinase B [a]	-	-	0.52 (0.36-0.74)	0.0026
	FER [a]	0.71 (0.55-0.91)	0.016	0.67 (0.51-0.87)	0.004
	RAN [a]	-	-	0.60 (0.42-0.82)	0.0063

S4 Table (continued)

Metabolism and Mitochondria	RAC1	0.58 (0.40-0.82)	0.022	0.52 (0.35-0.76)	0.0033
	α-Synuclein [a]	-	-	0.53 (0.36-0.75)	0.0031
	**Cyclophilin F**	0.72 (0.57-0.91)	0.016	0.63 (0.50-0.84)	0.003
	Cyclophilin A [a]	0.42 (0.24-0.73)	0.0222	0.31 (0.16-0.57)	0.0026
	SUMO3	-	-	0.38 (0.21-0.63)	0.0026
	UFM1	-	-	0.48 (0.31-0.70)	0.0026
	SNAA	-	-	0.47 (0.31-0.69)	0.0026
	VAV [a]	0.70 (0.53-0.90)	0.040	0.66 (0.49-0.86)	0.006
	**14-3-3 protein β/α** [a]	0.50 (0.30-0.79)	0.0281	0.41 (0.24-0.68)	0.0032
	14-3-3 protein ζ/δ [a]	0.45 (0.28-0.70)	0.0160	0.42 (0.26-0.68)	0.0031
	BAD	-	-	0.45 (0.27-0.70)	0.0031
	Triosephosphate isomerase	-	-	0.37 (0.21-0.61)	0.0026
	RPS6KA3	0.61 (0.40-0.89)	0.048	0.46 (0.29-0.71)	0.0031
	METAP1	-	-	0.50 (0.32-0.76)	0.0044
	Ubiquitin+1 [a]	-	-	0.37 (0.10-0.66)	0.0045
	UBC9	-	-	0.38 (0.21-0.67)	0.0026
	UBE2N [a]	-	-	0.46 (0.31-0.68)	0.0026
	**Carbonic anhydrase XIII**	0.80 (0.65-0.99)	0.038	0.74 (0.59-0.94)	0.011
	SRCN1	0.64 (0.49-0.83)	0.016	0.75 (0.57-0.97)	0.035
	M2-PK	0.55 (0.39-0.77)	0.016	0.72 (0.53-0.98)	0.045
	PPAC	-	-	0.64 (0.47-0.85)	0.006
	Transketolase	-	-	0.38 (0.22-0.63)	0.0026
	Aflatoxin B1 aldehyde reductase	-	-	0.61 (0.43-0.84)	0.007
	NCC27	-	-	0.46 (0.25-0.80)	0.0129
	PPID	0.60 (0.43-0.81)	0.016	0.70 (0.51-0.95)	0.033
	Myokinase	-	-	0.64 (0.48-0.85)	0.006
	NACA	-	-	0.53 (0.35-0.79)	0.0061
	SGTA	-	-	0.64 (0.46-0.88)	0.013
	**H2A3**	0.87 (0.69-1.09)	0.2495	0.81 (0.63-1.04)	0.014
	**BTK** [a]	0.72 (0.57-0.92)	0.016	0.63 (0.49-0.82)	0.003
	RPS6KA3	0.61 (0.40-0.89)	0.048	0.46 (0.29-0.71)	0.0031
	DUS3	-	-	0.50 (0.30-0.78)	0.0072
	RAC1 [a]	0.58 (0.40-0.82)	0.022	0.52 (0.35-0.76)	0.0033
	**CD40 ligand**	-	-	0.54 (0.35-0.81)	0.0074
	CPNE1 [a]	0.56 (0.35-0.88)	0.048	0.51 (0.30-0.83)	0.0142
	IMB1	-	-	0.60 (0.43-0.80)	0.003
	PTP-1C	-	-	0.63 (042-0.91)	0.023
	SRCN1	0.64 (0.49-0.83)	0.016	0.75 (0.57-0.97)	0.035
	Annexin I	-	-	0.59 (0.39-0.88)	0.0164
	BARK1	-	-	0.62 (0.41-0.92)	0.026
	GAPDH	0.64 (0.47-0.84)	0.022	0.59 (0.44-0.78)	0.003
	SP-D	-	-	0.76 (0.59-0.97)	0.034
	STAT3	0.47 (0.26-0.83)	0.0476	0.38 (0.20-0.71)	0.0026
	6-Phosphogluconate dehydrogenase	0.68 (0.53-0.85)	0.016	-	-
	ARGI1	0.62 (0.43-0.88)	0.047	-	-
	**Midkine**	-	-	1.5 (1.1-2.0)	0.014
	**Lactadherin (MFGM)**	-	-	1.9 (1.3-2.9)	0.006
**Tissue injury**	**MMP-12**	-	-	1.51 (1.07-2.15)	0.027
	**Renin**	-	-	1.88 (1.26-2.87)	0.006

[a] These proteins have multiples biological functions and, therefore, they belong to more than one biological process

Bold indicates the 15 proteins belonging to the short signature

Supporting information

S5 Table. Differences in clinical characteristics COPD patients from the MLCC cohort between Cluster 1 and Cluster 2 at inclusion

Parameter	Cluster 1 (n=34)	Cluster 2 (n=13)	p value *
Male sex – no. (%)	19 (55.9)	10 (76.9)	0.32
Age (years)	73.4 ± 9.3	70.4 ± 10.4	0.35
Caucasian origin – no. (%)	34 (100)	13 (100)	-
Other origin – no. (%)	0	0	-
GOLD stages			
GOLD 1 – no. (%)	2 (5.9)	0 (0.0)	0.80
GOLD 2 – no. (%)	12 (35.3)	4 (30.8)	0.80
GOLD 3 – no. (%)	13 (38.2)	6 (46.2)	0.80
GOLD 4 – no. (%)	7 (20.6)	3 (23.1)	0.80
Smoking history			
Never smokers – no. (%)	0	0	-
Former smoker – no. (%)	32 (94.1)	9 (69.2)	0.07
Packs per year in former smokers – no.	59.5 ± 35.0	47.7 ± 21.0	0.34
Active smokers – no. (%)	2 (5.9)	4 (30.8)	0.07
Packs per year in active smokers – no.	132.5 ± 10.6	67.5 ± 51.1	0.17
Body Mass Index (kg per m^2)	25.7 ± 5.1	23.4 ± 5.6)	0.30
Biology			
Blood leukocytes (no. per mm^3)	7700 (6400-9500)	8200 (7375-9800)	0.40
Blood eosinophils (no. per mm^3)	200 (100-300)	100 (75-200)	0.14
With blood eosinophils ≥ 300 per mm^3 – no. (%)	12 (36.4)	2 (6.7)	0.37
Blood neutrophils (no. per mm^3)	5000 (4100-6400)	5350 (4975- 6350)	0.29
Blood lymphocytes (no. per mm^3)	1500 (1100-2200)	1650 (1475-1925)	0.55
Blood monocytes (no. per mm^3)	700 (500-800)	700 (675-1000)	0.24
Hemoglobin – g per deciliter	12.9 (1.7)	13.5 (1.5)	0.32
CRP – mg per Liter	1.5 (3.4)	1.1 (1.0)	0.73
With CRP ≥ 3 mg per Liter – no. (%)	2 (8.3)	0 (0.0)	0.89
Respiratory function			
Pre-bronchodilator FEV_1 (% predicted)	44.6 (15.8)	33.0 (12.0)	0.11
Post-bronchodilator FEV_1 (% predicted)	45.9 (16.4)	35.5 (12.6)	0.17
Pre-bronchodilator FVC (% predicted)	90.5 (16.2)	84.5 (24.8)	0.49
Post-bronchodilator FVC (% predicted)	95.3 (20.5)	87.7 (22.1)	0.44
Pre-bronchodilator FEV_1 / FVC (% predicted)	38.2 (9.9)	30.0 (4.4)	0.06
Post-bronchodilator FEV_1 / FVC (% predicted)	37.8 (10.2)	30.8 (5.0)	0.13
DLCO (%)	47.3 (13.6)	37.2 (5.9)	0.13

S5 Table (continued)

Symptoms			
With cough – no (%)	28 (82.4)	10 (83.3)	1.00
With wheezing – no (%)	3 (8.8)	4 (30.8)	0.15
With emphysema no. (%)	30 (88.9)	5 (38.5)	**0.001**
Comorbidities			
Cardiovascular – no. (%)	18 (52.9)	4 (30.8)	0.20
Diabetes – no. (%)	8 (23.5)	1 (7.7)	0.11
Treatments			
On SABA – no. (%)	32 (94.1)	12 (92.3)	1.00
On LABA – no. (%)	32 (94.1)	9 (69.3)	**0.04**
On LAMA – no. (%)	32 (100.0)	10 (90.9)	0.57
On ICS – no. (%)	32 (94.1)	11 (84.6)	0.65
Daily dose of equivalents beclomethasone	864.1 ± 382.3	752.5 ± 298.2	0.45
On oxygen therapy – no. (%)	21 (61.8)	6 (46.2)	0.52

Data are n (%), or means ± SD, or median (25-75 interquartile range), or means ± SEM, for the number of exacerbations

CRP = C reactive protein; FEV_1 = Forced Expiratory Volume in 1 second; FVC = Forced Vital Capacity; DLCO = transfer factor of the lung for carbon monoxide; SABA = short-acting β2-agonists; LABA = long-acting β2-agonists; LAMA = long-lasting muscarinic antagonists; ICS = inhaled corticosteroids.

[*] Students' t test, or Fisher exact test 2-tailed, or Mann-Whitney U-test

[a] Estimated frequency of exacerbations and hospitalizations, according to the events during the study period

Supporting information

S6 Table. Main clinical characteristics of COPD patients switching of Clusters between visit 1 and visit 2

Parameter	Values at visit 1	Values at visit 2	p value *
**COPD patients switching from Cluster 1 at visit 1 to Cluster 2 at visit 2 (n = 29)**			
With emphysema - no. (%) [b]	16 (55.2)	13 (44.8)	0.44
DLCO - %	49.4 ± 13.3	54.3 ± 28.5	0.60
With exacerbations in the previous 12 months - no. (%)	20 (69.0)	11 (37.9)	**0.02**
With unscheduled medical visits in the previous 12 months - no. (%)	16 (55.2)	8 (27.6)	**0.04**
With hospitalizations for COPD in the previous 12 months – no. (%)	8 (27.6)	1 (3.4)	**0.02**
On SABA – no. (%)	19 (65.5)	18 (69.2)	1.00
On LABA – no. (%)	22 (75.9)	20 (70)	0.77
On LAMA – no. (%)	19 (65.5)	20 (70)	1.00
On ICS – no. (%)	13 (44.8)	15 (51.7)	0.79
On anti-hypertensive drugs – no. (%)	12 (41.4)	10 (38.5)	0.79
On statins – no. (%)	11 (37.9)	10 (38.5)	1.00
Adherence to treatment – no. (%)	26 (89.7)	26 (89.7)	1.00
**COPD patients switching from Cluster 2 at visit 1 to Cluster 1 at visit 2 (n = 26)**			
With emphysema - no. (%)	5 (19.2)	11 (42.3)	0.08
DLCO - %	53.6 ± 18.0	53.4 ± 10.9	0.99
With exacerbations in the previous 12 months - no. (%)	15 (57.7)	10 (38.5)	0.17
With unscheduled medical visits in the previous 12 months - no. (%)	10 (38.5)	8 (30.8)	0.56
With hospitalizations for COPD in the previous 12 months – no. (%)	1 (4)	6 (23.1)	**0.05**
On SABA – no. (%)	20 (69)	19 (73.1)	1.00
On LABA – no. (%)	18 (62.1)	21 (80.8)	0.52
On LAMA – no. (%)	12 (46.2)	20 (76.9)	**0.04**
On ICS – no. (%)	13 (50)	15 (57.7)	0.74
On anti-hypertensive drugs – no. (%)	7 (24.1)	8 (30.8)	1.00
On statins – no. (%)	2 (6.9)	3 (11.5)	1.00
Adherence to treatments - no. (%)	23 (92.0)	24 (100.0)	1.00

Data are expressed as numbers (%) and as means ± SD

DLCO = transfer factor of the lung for carbon monoxide; SABA = short-acting β2-agonists; LABA = long-acting β2-agonists; LAMA = long-lasting muscarinic antagonists; ICS = inhaled corticosteroids.

* Students' t test or Fisher exact test 2-tailed
