# Supplementary material for: Proteomic profiling of serum identifies a molecular signature that correlates with clinical outcomes in COPD
Source: PLoS One. 2022 Dec 8;17(12):e0277357. doi: 10.1371/journal.pone.0277357 (PMC9731494; doi:10.1371/journal.pone.0277357)
Supplement: S1 Table — (PDF) [file pone.0277357.s002.pdf]

## Supporting information

### S1 Table: characteristics of COPD patients of the COBRA cohort

| Parameter | n * | All patients (n = 241) | n * | Patients with 1 visit (n=78) | n * | Patients with 2 visits (n = 163) | | p value [a] | p value [b] |
|---|---|---|---|---|---|---|---|---|---|
| | | **Visit 1** | | **Visit 1 only** | | **Visit 1** | **Visit 2** | | |
| Male sex – no. (%) | 241 | 164 (68.1) | 78 | 50 (64.1) | 163 | 114 (69.9) | - | 0.37 | - |
| Age (years) | 241 | 63.1 ± 9.8 | 78 | 61.6 ± 11.4 | 163 | 63.8 ± 8.9 | 64.5 ± 9.0 | 0.14 | - |
| Caucasian origin – no. (%) | 239 | 224 (93.7) | 76 | 70 (92.1) | 163 | 154 (94.5) | - | 0.49 | - |
| Other origin – no. (%) | 239 | 15 (6.3) | 76 | 6 (7.9) | 163 | 9 (5.5) | - | 0.49 | - |
| *GOLD stages* | | | | | | | | | |
| GOLD I – no. (%) | 238 | 54 (22.7) | 75 | 24 (32.0) | 159 | 28 (17.6) | 28 (17.6) | **0.03** | 0.99 |
| GOLD II – no. (%) | 238 | 87 (36.5) | 75 | 20 (26.6) | 159 | 66 (41.5) | 66 (41.5) | **0.03** | 0.99 |
| GOLD III – no. (%) | 238 | 53 (22.3) | 75 | 14 (18.7) | 159 | 38 (23.9) | 36 (22.7) | **0.03** | 0.99 |
| GOLD IV – no. (%) | 238 | 44 (18.5) | 75 | 17 (22.7) | 159 | 27 (17.0) | 29 (18.2) | **0.03** | 0.99 |
| *Smoking history* | | | | | | | | | |
| Former smoker – no. (%) | 241 | 149 (61.8) | 78 | 41 (52.6) | 163 | 108 (66.3) | 108 (66.3) | **0.002** | 1.00 |
| Packs per year in former smokers – no. | 147 | 49.1 ± 23.9 | 39 | 49.7 ± 22.2 | 101 | 49.7 ± 25.1 | 49.9 ± 25.1 | 0.99 | 0.96 |
| Active smokers – no. (%) | 241 | 84 (34.9) | 78 | 30 (38.5) | 163 | 54 (33.1) | 54 (33.1) | **0.002** | 1.00 |
| Packs per year in active smokers – no. | 82 | 35.8 ± 21.7 | 28 | 35.1 ± 20.8 | 47 | 36.0 ± 23.8 | 34.9 ± 21.9 | 0.89 | 0.83 |
| Body Mass Index (kg per $m^2$) | 240 | 26.5 ± 6.1 | 77 | 25.7 ± 5.9 | 163 | 26.8 ± 6.2 | - | 0.21 | - |
| *Biology* | | | | | | | | | |
| Blood leukocytes (no. per $mm^3$) | 165 | 7500 (6500 - 8900) | 43 | 7400 (6400 - 10000) | 96 | 7350 (6500 - 8750) | 7100 (6150 - 8150) | 0.37 | 0.34 |
| Blood eosinophils (no. per $mm^3$) | 165 | 162 (104 - 230) | 43 | 146 (94 - 213) | 96 | 165 (103 - 225) | 162 (117 - 244) | 0.37 | 0.67 |
| With blood eosinophils ≥ 300 per $mm^3$ – no. (%) | 165 | 27 (16.4) | 43 | 4 (9.3) | 96 | 17 (17.7) | 12 (12.5) | 0.21 | 0.32 |
| Blood neutrophils (no. per $mm^3$) | 163 | 4752 (3840 - 5950) | 42 | 4479 (3782 - 6486) | 95 | 4680 (3835 - 5950) | 4560 (3843 - 5265) | 0.74 | 0.39 |
| Blood lymphocytes (no. per $mm^3$) | 165 | 1830 (1406 - 2280) | 43 | 1920 (1539 - 2438) | 96 | 1648 (1302 - 2190) | 1782 (1387 - 2184) | **0.04** | 0.49 |
| Blood monocytes (no. per $mm^3$) | 161 | 560 (444 - 720) | 40 | 603 (469 - 740) | 96 | 532 (404 - 698) | 555 (463 - 667) | **0.03** | 0.25 |
| Hemoglobin – g per deciliter | 115 | 14.4 ± 1.4 | 32 | 14.1 ± 1.6 | 72 | 14.6 ± 1.3 | 14.3 ± 1.4 | 0.10 | 0.25 |
| CRP – mg per Liter | 112 | 7.6 ± 7.6 | 22 | 6.5 ± 4.6 | 73 | 7.8 ± 8.1 | 6.8 ± 7.5 | 0.35 | 0.47 |
| With CRP ≥ 3 mg per Liter – no. (%) | 112 | 85 (75.9) | 22 | 17 (77.3) | 73 | 54 (74.0) | 54 (74.0) | 0.76 | 1.00 |
| *Blood gases* | | | | | | | | | |
| $PaO_2$ - mmHg | 181 | 74.5 ± 14.1 | 57 | 74.8 ± 14.2 | 91 | 72.9 ± 13.7 | 74.7 ± 13.2 | 0.42 | 0.37 |
| $PaCO_2$ - mmHg | 182 | 39.6 ± 4.8 | 57 | 40.0 ± 4.8 | 90 | 39.8 ± 4.9 | 40.0 ± 6.0 | 0.86 | 0.86 |
| pH | 182 | 7.42 ± 0.03 | 57 | 7.41 ± 0.03 | 92 | 7.41 ± 0.03 | 7.41 ± 0.04 | 0.78 | 0.70 |

## S1 Table (continued)

| | | | | | | | | | |
|---|---|---|---|---|---|---|---|---|---|
| *Respiratory function* | | | | | | | | | |
| Pre-bronchodilator $FEV_1$ (% predicted) | 233 | 59.6 ± 24.3 | 72 | 63.0 ± 27.7 | 144 | 56.6 ± 21.7 | 56.1 ± 21.7 | 0.10 | 0.84 |
| Post-bronchodilator $FEV_1$ (% predicted) | 171 | 63.7 ± 25.8 | 53 | 71.3 ± 30.4 | 70 | 56.6 ± 22.8 | 56.5 ± 21.5 | **0.005** | 0.98 |
| With fixed airflow obstruction – no. (%) [c] | 176 | 143 (81.3) | 55 | 34 (61.8) | 70 | 65 (92.9) | 65 (92.9) | **< 0.0001** | 1.00 |
| Pre-bronchodilator FVC (% predicted) | 233 | 86.6 ± 21.3 | 73 | 86.1 ± 21.9 | 142 | 85.0 ± 20.4 | 84.0 ± 20.4 | 0.73 | 0.68 |
| Post-bronchodilator FVC (% predicted) | 169 | 91.5 ± 21.1 | 51 | 91.9 ± 21.5 | 69 | 91.0 ± 20.4 | 88.8 ± 21.1 | 0.83 | 0.54 |
| Pre-bronchodilator $FEV_1$ / FVC (% predicted) | 235 | 53.5 ± 15.3 | 74 | 57.0 ± 16.7 | 144 | 51.4 ± 14.0 | 51.7 ± 14.2 | **0.01** | 0.89 |
| Post-bronchodilator $FEV_1$ / FVC (% predicted) | 176 | 53.9 ± 16.2 | 55 | 60.6 ± 17.3 | 70 | 47.2 ± 14.4 | 48.4 ± 14.7 | **< 0.0001** | 0.62 |
| FRC - % | 124 | 137.8 ± 38.3 | 49 | 131.5 ± 31.7 | 37 | 140.9 ± 43.4 | 139.9 ± 41.5 | 0.27 | 0.92 |
| RV - % | 135 | 156.4 ± 53.0 | 53 | 150.7 ± 43.1 | 41 | 165.9 ± 62.9 | 154.7 ± 56.6 | 0.19 | 0.41 |
| TLC - % | 137 | 113.8 ± 19.2 | 55 | 112.0 ± 16.9 | 42 | 114.1 ± 21.7 | 112.4 ± 19.5 | 0.60 | 0.70 |
| DLCO - % | 115 | 59.0 ± 21.7 | 46 | 63.9 ± 21.6 | 34 | 49.5 ± 18.8 | 49.9 ± 18.8 | **0.003** | 0.94 |
| *Symptoms* | | | | | | | | | |
| With cough – no. (%) | 241 | 97 (40.2) | 78 | 27 (34.6) | 163 | 70 (42.9) | 56 (34.4) | 0.22 | 0.12 |
| With wheezing – no. (%) | 241 | 32 (13.3) | 78 | 14 (17.9) | 163 | 18 (11.0) | 16 (9.8) | 0.14 | 0.72 |
| With emphysema no. (%) [d] | 241 | 92 (38.2) | 78 | 28 (35.9) | 163 | 64 (39.3) | 64 (39.3) | 0.62 | 1.00 |
| With exacerbations in the previous 12 months - no. (%) | 241 | 128 (53.1) | 78 | 39 (50.0) | 163 | 89 (54.6) | 62 (38.0) | 0.51 | **0.003** |
| Exacerbations in the previous 12 months - no. | 241 | 1.44 ± 0.16 | 78 | 1.26 ± 0.22 | 163 | 1.52 ± 0.21 | 1.46 ± 0.23 | 0.12 | 0.72 |
| Unscheduled medical visits in the previous 12 months - no. | 241 | 1.19 ± 0.17 | 78 | 0.96 ± 0.23 | 163 | 1.29 ± 0.22 | 1.08 ± 0.16 | **0.03** | 0.09 |
| Hospitalizations for COPD in the previous 12 months – no. | 241 | 0.32 ± 0.05 | 78 | 0.41 ± 0.11 | 163 | 0.27 ± 0.05 | 0.19 ± 0.06 | 0.10 | 0.17 |
| *Comorbidities* | | | | | | | | | |
| Cardiovascular – no. (%) | 241 | 103 (42.7) | 78 | 32 (41.0) | 163 | 71 (43.6) | - | 0.71 | - |
| Hypertension – no. (%) | 241 | 67 (27.8) | 78 | 21 (26.9) | 163 | 46 (28.2) | - | 0.84 | - |
| Dyslipidemia – no. (%) | 241 | 58 (24.1) | 78 | 14 (17.9) | 163 | 44 (27.0) | - | 0.13 | - |
| Diabetes – no. (%) | 241 | 28 (11.6) | 78 | 7 (9.0) | 163 | 21 (12.9) | - | 0.38 | - |
| Sinusitis – no. (%) | 241 | 27 (11.2) | 78 | 7 (9.0) | 163 | 20 (12.3) | - | 0.45 | - |
| Allergic rhinitis – no. (%) | 241 | 38 (15.8) | 78 | 12 (15.4) | 163 | 26 (15.9) | - | 0.92 | - |
| Obstructive sleep apnea – no. (%) | 241 | 22 (9.1) | 78 | 4 (5.1) | 163 | 18 (11.0) | - | 0.14 | - |
| Gastro Esophageal Reflux – no. (%) | 241 | 70 (29.0) | 78 | 21 (26.9) | 163 | 49 (30.1) | - | 0.62 | - |
| Bronchial dilatation – no. (%) | 241 | 11 (4.6) | 78 | 3 (3.8) | 163 | 8 (4.9) | - | 0.72 | - |

**S1 Table (continued)**

| | | | | | | | | | |
|---|---|---|---|---|---|---|---|---|---|
| *Treatments* | | | | | | | | | |
| On SABA – no. (%) | 231 | 144 (62.3) | 70 | 33 (47.1) | 158 | 110 (69.6) | 115 (72.8) | **0.002** | 0.54 |
| On LABA alone – no. (%) | 233 | 26 (11.2) | 70 | 5 (7.1) | 160 | 21 (13.1) | 18 (11.2) | 0.19 | 0.61 |
| On LAMA alone – no. (%) | 233 | 16 (6.9) | 70 | 4 (5.7) | 160 | 12 (7.5) | 12 (7.5) | 0.63 | 1.00 |
| On ICS alone – no. (%) | 233 | 4 (1.7) | 70 | 1 (1.4) | 160 | 3 (1.9) | 3 (1.9) | 0.82 | 1.00 |
| Daily dose of ICS - µg of equivalents beclomethasone | 119 | 1304 ± 669 | 28 | 1499 ± 811 | 77 | 1253 ± 633 | 1242 ± 626 | 0.11 | 0.92 |
| On OCS – no. (%) | 232 | 8 (3.4) | 69 | 7 (10.1) | 160 | 1 (0.6) | 2 (1.2) | **0.0003** | 0.57 |
| Daily dose of prednisone (mg) | 8 | 20.4 ± 10.9 | 7 | 22.6 ± 9.6 | 1 | 5 | 5 | 0.14 | - |
| On LABA + LAMA – no. (%) | 233 | 22 (9.4) | 70 | 6 (8.6) | 160 | 16 (10.0) | 21 (13.1) | 0.74 | 0.39 |
| On LABA + LAMA + ICS – no. (%) | 233 | 78 (33.5) | 70 | 22 (31.4) | 160 | 55 (34.4) | 63 (39.4) | 0.67 | 0.36 |
| On anti-histamine – no. (%) | 241 | 20 (8.3) | 78 | 8 (10.3) | 162 | 12 (7.4) | 8 (4.9) | 0.46 | 0.36 |
| On theophylline – no. (%) | 232 | 1 (0.4) | 69 | 0 (0.0) | 160 | 1 (0.6) | 1 (0.6) | 0.52 | 1.00 |
| On gastro esophageal reflux inhibitors – no. (%) | 241 | 56 (23.2) | 78 | 17 (21.8) | 163 | 39 (23.9) | 29 (17.8) | 0.72 | 0.18 |
| On anti-hypertensive drugs – no. (%) | 232 | 80 (34.5) | 70 | 24 (34.3) | 160 | 55 (34.4) | 54 (33.7) | 0.99 | 0.91 |
| On statins – no. (%) | 231 | 56 (24.2) | 69 | 11 (15.9) | 160 | 44 (27.5) | 49 (30.6) | 0.07 | 0.54 |
| On fibrates – no. (%) | 229 | 5 (2.2) | 69 | 1 (1.4) | 157 | 4 (2.5) | 3 (1.9) | 0.61 | 0.71 |
| On anti-platelet aggregation – no. (%) | 231 | 63 (27.3) | 69 | 15 (21.7) | 159 | 47 (29.6) | 42 (26.4) | 0.23 | 0.54 |
| Other – no. (%) | 227 | 102 (44.9) | 68 | 20 (29.4) | 157 | 81 (51.6) | 75 (47.8) | **0.003** | 0.50 |
| On oxygen therapy – no. (%) | 238 | 37 (15.5) | 75 | 11 (14.7) | 161 | 26 (16.1) | 16 (9.9) | 0.78 | 0.10 |
| Adherence to treatments – no. (%) | 224 | 203 (90.6) | 64 | 55 (85.9) | 155 | 144 (92.9) | 143 (92.3) | 0.11 | 0.83 |

Data are n (%), or means ± SD, or median (25-75 IQR), or means ± SEM, in case of number of exacerbations, of unscheduled hospital visits and hospitalizations for COPD in the previous 6 months

CRP = C reactive protein; $FEV_1$ = Forced Expiratory Volume in 1 second; FVC = Forced Vital Capacity; FRC = Functional Residual Capacity; RV = Residual Volume; TLC = Total Lung Capacity; DLCO = transfer factor of the lung for carbon monoxide; ICS = inhaled corticosteroids; SABA = short-acting β2-agonists; LABA = long-acting β2-agonists; OCS = oral corticosteroids; LAMA = long-lasting muscarinic antagonists.

* denotes the number of patients with each available variable

[a] p ≤ 0.05, between COPD patients with 1 and 2 visits (n=78 and n=163, respectively) (Students' t test, or Mann-Whitney U-test, or Fisher exact test, 2-tailed, or Poisson test).
[b] p ≤ 0.05, between visits 1 and 2 for n=163 COPD patients (Students' t test, or Mann-Whitney U-test, or Fisher exact test, 2-tailed, or Poisson test).
