# Supplementary material for: Proteomic profiling of serum identifies a molecular signature that correlates with clinical outcomes in COPD
Source: PLoS One. 2022 Dec 8;17(12):e0277357. doi: 10.1371/journal.pone.0277357 (PMC9731494; doi:10.1371/journal.pone.0277357)
Supplement: S2 Table — (PDF) [file pone.0277357.s003.pdf]

Supporting information

S2 Table: characteristics of COPD patients of the MLCC cohort

Parameter	n *	All patients, n = 47
Male sex – no. (%)	47	29 (61.7)
Age (years)	47	72.5 ± 9.6
Caucasian origin – no. (%)	47	47 (100)
Other origin – no. (%)	47	0
GOLD stages		
GOLD 1 – no. (%)	47	2 (4.3)
GOLD 2 – no. (%)	47	16 (34.0)
GOLD 3 – no. (%)	47	19 (40.4)
GOLD 4 – no. (%)	47	10 (21.3)
Smoking history		
Never smokers – no. (%)	47	0
Former smoker – no. (%)	47	41 (87.2)
Packs per year in former smokers – no.	47	56.9 ± 32.6
Active smokers – no. (%)	47	6 (12.8)
Packs per year in active smokers – no.	47	89.2 ± 52.1
Body Mass Index (kg per m^2)	31	25.2 ± 6.0
Biology		
Blood leukocytes (no. per mm^3)	45	7800 (6500-9800)
Blood eosinophils (no. per mm^3)	45	200 (100-300)
With blood eosinophils ≥ 300 per mm^3 – no. (%)	45	14 (31.1)
Blood neutrophils (no. per mm^3)	45	5300 (4300-6400)
Blood lymphocytes (no. per mm^3)	45	1600 (1100-2000)
Blood monocytes (no. per mm^3)	45	700 (600-800)
Hemoglobin – g per deciliter	45	13.1 ± 1.6
CRP – mg per Liter	34	1.4 ± 2.9
With CRP ≥ 3 mg per Liter – no. (%)	34	2 (5.9)
Blood gases		
PaO_2 - mmHg	9	66.1 ± 5.4
$PaCO_2$ - mmHg	9	40.5 ± 4.6
Respiratory function		
Pre-bronchodilator FEV_1 (% predicted)	28	39.1 ± 10.5
Post-bronchodilator FEV_1 (% predicted)	29	41.9 ± 14.2
Pre-bronchodilator FVC (% predicted)	28	87.1 ± 16.9
Post-bronchodilator FVC (% predicted)	30	93.1 ± 18.0
Pre-bronchodilator FEV_1 / FVC (% predicted)	28	35.5 ± 9.0
Post-bronchodilator FEV_1 / FVC (% predicted)	29	35.5 ± 10.0
With fixed airflow obstruction – no. (%) [a]	29	28 (96.6)
DLCO (%)	25	41.4 ± 12.4
Symptoms		
With cough – no (%)	46	38 (82.6)
With wheezing – no (%)	47	7 (14.9)
With emphysema no. (%)	25	24 (96.0)
Exacerbations in 12 months - no. (%) [b]	47	1.6 ± 0.7
Hospitalizations for COPD in 12 months - no. [b]	17	0.7 ± 0.7

S2 Table (continued)

Comorbidities		
Cardiovascular – no. (%)	47	22 (46.8)
Diabetes – no. (%)	47	9 (19.1)
Obstructive sleep apnea – no. (%)	47	1 (2.1)
Treatments		
On SABA – no. (%)	47	44 (93.6)
On LABA – no. (%)	43	42 (97.7)
On LAMA – no. (%)	47	25 (53.0)
On ICS – no. (%)	47	43 (91.5)
Daily dose of equivalents beclomethasone	40	842 ± 366
On OCS – no. (%)	47	4 (8.5)
Daily dose of prednisone (mg)	4	4.9 ± 2.3
On oxygen therapy – no. (%)	47	27 (57.4)
Adherence to treatment – no. (%)	12	8 (66.7)

CRP = C reactive protein; FEV_1 = Forced Expiratory Volume in 1 second; FVC = Forced Vital Capacity; DLCO = transfer factor of the lung for carbon monoxide; SABA = short-acting β2-agonists; LABA = long-acting β2-agonists; LAMA = long-lasting muscarinic antagonists; ICS = inhaled corticosteroids = OCS, oral corticosteroids.

Data are n (%), or means ± SD, or median (25-75 IQR), or means ± SEM, for the number of exacerbations

* denotes the number of patients with each available variable

[b] Estimated frequency of exacerbations and hospitalizations based on the events during the study period
