# Supplementary material for: Proteomic profiling of serum identifies a molecular signature that correlates with clinical outcomes in COPD
Source: PLoS One. 2022 Dec 8;17(12):e0277357. doi: 10.1371/journal.pone.0277357 (PMC9731494; doi:10.1371/journal.pone.0277357)
Supplement: S4 Table — (PDF) [file pone.0277357.s005.pdf]

Supporting information

S4 Table: Significant correlations between differentially regulated proteins and the incidence of exacerbations and emphysema in patients with COPD at visit 1.

Biological Hallmarks	Target	Exacerbations (Y/N)		Emphysema (Y/N)	
		OR (2.5-97.5%)	FDR	OR (2.5-97.5%)	FDR
Cell fate, Remodeling and Repair	PRKCA [a]	0.57 (0.41-0.79)	0.016	-	-
	PRKACA [a]	-	-	0.62 (0.41-0.90)	0.0200
	SHC1	-	-	0.33 (0.17-0.58)	0.0026
	eIF-5A-1	-	-	0.43 (0.18-0.60)	0.0026
	TCTP	0.57 (0.36-0.87)	0.0476	0.35 (0.20-0.58)	0.0026
	PA2G4	-	-	0.40 (0.24-0.64)	0.0026
	**14-3-3 protein β/α [a]**	0.50 (0.30-0.79)	0.0281	0.41 (0.24-0.68)	0.0032
	14-3-3 protein ζ/δ [a]	0.45 (0.28-0.70)	0.0160	0.42 (0.26-0.68)	0.0031
	BAD [a]	-	-	0.45 (0.27-0.70)	0.0031
	RPS6KA3 [a]	0.61 (0.40-0.89)	0.048	0.46 (0.29-0.71)	0.0031
	**AREG**	-	-	0.46 (0.27-0.75)	0.0065
	METAP1 [a]	-	-	0.50 (0.32-0.76)	0.0044
	**FGF16**	-	-	0.52 (0.31-0.81)	0.0115
	RAC1 [a]	0.58 (0.40-0.82)	0.022	0.52 (0.35-0.76)	0.0033
	SBDS	0.66 (0.48-0.92)	0.048	0.53 (0.36-0.76)	0.0031
	hnRNP A2/B1	-	-	0.53 (0.36-0.79)	0.0060
	NSF1C	-	-	0.55 (0.39-0.77)	0.0031
	PKC-A [a]	-	-	0.55 (0.38-0.78)	0.0042
	SMAD2 [a]	0.67 (0.49-0.92)	0.048	0.58 (0.40-0.81)	0.0059
	**eIF-4H**	0.63 (0.45-0.86)	0.028	0.59 (0.41-0.82)	0.0061
	DRG-1	-	-	0.51 (0.35-0.72)	0.0026
	DLRB1	-	-	0.61 (0.39-0.91)	0.025
	PLPP	-	-	0.36 (0.20-0.62)	0.0026
	ARGI1	0.62 (0.43-0.88)	0.047	-	-
	IMB1 [a]	-	-	0.60 (0.43-0.80)	0.003
	ARGI1 [a]	0.62 (0.43-0.88)	0.047	-	-
	41	-	-	0.62 (0.44-0.86)	0.010
	GRB2 adapter protein	0.72 (0.55-0.93)	0.017	0.64 (0.49-0.85)	0.004
	**Tropomyosin 4**	0.78 (0.64-0.96)	0.024	0.71 (0.57-0.89)	0.004
	Sphingosine kinase 1	0.66 (0.47-0.91)	0.048	0.60 (0.41-0.85)	0.008
	Hemoglobin	-	-	0.72 (0.54-0.93)	0.027
	CSK	-	-	0.64 (0.47-0.86)	0.006
	FYN [a]	-	-	0.74 (0.56-0.97)	0.042
	ERK-1 [a]	0.50 (0.29-0.84)	0.0476	0.41 (0.22-0.72)	0.0062
	MK01/ERK-2	-	-	0.45 (0.26-0.74)	0.0061
	PKC-B-II	0.59 (0.43-0.80)	0.0158	0.55 (0.40-0.76)	0.0026
	Prostatic binding protein	-	-	0.41 (0.24-0.68)	0.0032
	Caspase-3	0.66 (0.47-0.91)	0.048	0.52 (0.35-0.74)	0.0030
	PDPK1 [a]	-	-	0.61 (0.43-0.87)	0.011
	LYN [a]	0.65 (0.47-0.87)	0.029	0.65 (0.47-0.89)	0.013
	LYNB [a]	0.65 (0.48-0.87)	0.028	0.68 (0.50-0.91)	0.016
	CK2-A1:B	-	-	0.56 (0.40-8.80)	0.0164
	NDP kinase B [a]	-	-	0.52 (0.36-0.74)	0.0026
	FER [a]	0.71 (0.55-0.91)	0.016	0.67 (0.51-0.87)	0.004
	RAN [a]	-	-	0.60 (0.42-0.82)	0.0063

S4 Table (continued)

	RAC1	0.58 (0.40-0.82)	0.022	0.52 (0.35-0.76)	0.0033
	α-Synuclein [a]	-	-	0.53 (0.36-0.75)	0.0031
	**Cyclophilin F**	0.72 (0.57-0.91)	0.016	0.63 (0.50-0.84)	0.003
	Cyclophilin A [a]	0.42 (0.24-0.73)	0.0222	0.31 (0.16-0.57)	0.0026
	SUMO3	-	-	0.38 (0.21-0.63)	0.0026
	UFM1	-	-	0.48 (0.31-0.70)	0.0026
	SNAA	-	-	0.47 (0.31-0.69)	0.0026
	VAV [a]	0.70 (0.53-0.90)	0.040	0.66 (0.49-0.86)	0.006
	**14-3-3 protein β/α** [a]	0.50 (0.30-0.79)	0.0281	0.41 (0.24-0.68)	0.0032
	14-3-3 protein ζ/δ [a]	0.45 (0.28-0.70)	0.0160	0.42 (0.26-0.68)	0.0031
	BAD	-	-	0.45 (0.27-0.70)	0.0031
	Triosephosphate isomerase	-	-	0.37 (0.21-0.61)	0.0026
	RPS6KA3	0.61 (0.40-0.89)	0.048	0.46 (0.29-0.71)	0.0031
	METAP1	-	-	0.50 (0.32-0.76)	0.0044
	Ubiquitin+1 [a]	-	-	0.37 (0.10-0.66)	0.0045
	UBC9	-	-	0.38 (0.21-0.67)	0.0026
	UBE2N [a]	-	-	0.46 (0.31-0.68)	0.0026
	**Carbonic anhydrase XIII**	0.80 (0.65-0.99)	0.038	0.74 (0.59-0.94)	0.011
	SRCN1	0.64 (0.49-0.83)	0.016	0.75 (0.57-0.97)	0.035
	M2-PK	0.55 (0.39-0.77)	0.016	0.72 (0.53-0.98)	0.045
	PPAC	-	-	0.64 (0.47-0.85)	0.006
	Transketolase	-	-	0.38 (0.22-0.63)	0.0026
	Aflatoxin B1 aldehyde reductase	-	-	0.61 (0.43-0.84)	0.007
**Metabolism and Mitochondria**	NCC27	-	-	0.46 (0.25-0.80)	0.0129
	PPID	0.60 (0.43-0.81)	0.016	0.70 (0.51-0.95)	0.033
	Myokinase	-	-	0.64 (0.48-0.85)	0.006
	NACA	-	-	0.53 (0.35-0.79)	0.0061
	SGTA	-	-	0.64 (0.46-0.88)	0.013
	**H2A3**	0.87 (0.69-1.09)	0.2495	0.81 (0.63-1.04)	0.014
	**BTK** [a]	0.72 (0.57-0.92)	0.016	0.63 (0.49-0.82)	0.003
	RPS6KA3	0.61 (0.40-0.89)	0.048	0.46 (0.29-0.71)	0.0031
	DUS3	-	-	0.50 (0.30-0.78)	0.0072
	RAC1 [a]	0.58 (0.40-0.82)	0.022	0.52 (0.35-0.76)	0.0033
	**CD40 ligand**	-	-	0.54 (0.35-0.81)	0.0074
	CPNE1 [a]	0.56 (0.35-0.88)	0.048	0.51 (0.30-0.83)	0.0142
	IMB1	-	-	0.60 (0.43-0.80)	0.003
	PTP-1C	-	-	0.63 (042-0.91)	0.023
	SRCN1	0.64 (0.49-0.83)	0.016	0.75 (0.57-0.97)	0.035
	Annexin I	-	-	0.59 (0.39-0.88)	0.0164
	BARK1	-	-	0.62 (0.41-0.92)	0.026
	GAPDH	0.64 (0.47-0.84)	0.022	0.59 (0.44-0.78)	0.003
	SP-D	-	-	0.76 (0.59-0.97)	0.034
	STAT3	0.47 (0.26-0.83)	0.0476	0.38 (0.20-0.71)	0.0026
	6-Phosphogluconate dehydrogenase	0.68 (0.53-0.85)	0.016	-	-
	ARGI1	0.62 (0.43-0.88)	0.047	-	-
	**Midkine**	-	-	1.5 (1.1-2.0)	0.014
	**Lactadherin (MFGM)**	-	-	1.9 (1.3-2.9)	0.006
**Tissue injury**	**MMP-12**	-	-	1.51 (1.07-2.15)	0.027
	**Renin**	-	-	1.88 (1.26-2.87)	0.006

[a] These proteins have multiples biological functions and, therefore, they belong to more than one biological process

Bold indicates the 15 proteins belonging to the short signature
