# Supplementary material for: Proteomic profiling of serum identifies a molecular signature that correlates with clinical outcomes in COPD
Source: PLoS One. 2022 Dec 8;17(12):e0277357. doi: 10.1371/journal.pone.0277357 (PMC9731494; doi:10.1371/journal.pone.0277357)
Supplement: S5 Table — (PDF) [file pone.0277357.s006.pdf]

Supporting information

S5 Table. Differences in clinical characteristics COPD patients from the MLCC cohort between Cluster 1 and Cluster 2 at inclusion

Parameter	Cluster 1 (n=34)	Cluster 2 (n=13)	p value *
Male sex – no. (%)	19 (55.9)	10 (76.9)	0.32
Age (years)	73.4 ± 9.3	70.4 ± 10.4	0.35
Caucasian origin – no. (%)	34 (100)	13 (100)	-
Other origin – no. (%)	0	0	-
GOLD stages			
GOLD 1 – no. (%)	2 (5.9)	0 (0.0)	0.80
GOLD 2 – no. (%)	12 (35.3)	4 (30.8)	0.80
GOLD 3 – no. (%)	13 (38.2)	6 (46.2)	0.80
GOLD 4 – no. (%)	7 (20.6)	3 (23.1)	0.80
Smoking history			
Never smokers – no. (%)	0	0	-
Former smoker – no. (%)	32 (94.1)	9 (69.2)	0.07
Packs per year in former smokers – no.	59.5 ± 35.0	47.7 ± 21.0	0.34
Active smokers – no. (%)	2 (5.9)	4 (30.8)	0.07
Packs per year in active smokers – no.	132.5 ± 10.6	67.5 ± 51.1	0.17
Body Mass Index (kg per m^2)	25.7 ± 5.1	23.4 ± 5.6)	0.30
Biology			
Blood leukocytes (no. per mm^3)	7700 (6400-9500)	8200 (7375-9800)	0.40
Blood eosinophils (no. per mm^3)	200 (100-300)	100 (75-200)	0.14
With blood eosinophils ≥ 300 per mm^3 – no. (%)	12 (36.4)	2 (6.7)	0.37
Blood neutrophils (no. per mm^3)	5000 (4100-6400)	5350 (4975- 6350)	0.29
Blood lymphocytes (no. per mm^3)	1500 (1100-2200)	1650 (1475-1925)	0.55
Blood monocytes (no. per mm^3)	700 (500-800)	700 (675-1000)	0.24
Hemoglobin – g per deciliter	12.9 (1.7)	13.5 (1.5)	0.32
CRP – mg per Liter	1.5 (3.4)	1.1 (1.0)	0.73
With CRP ≥ 3 mg per Liter – no. (%)	2 (8.3)	0 (0.0)	0.89
Respiratory function			
Pre-bronchodilator FEV_1 (% predicted)	44.6 (15.8)	33.0 (12.0)	0.11
Post-bronchodilator FEV_1 (% predicted)	45.9 (16.4)	35.5 (12.6)	0.17
Pre-bronchodilator FVC (% predicted)	90.5 (16.2)	84.5 (24.8)	0.49
Post-bronchodilator FVC (% predicted)	95.3 (20.5)	87.7 (22.1)	0.44
Pre-bronchodilator FEV_1 / FVC (% predicted)	38.2 (9.9)	30.0 (4.4)	0.06
Post-bronchodilator FEV_1 / FVC (% predicted)	37.8 (10.2)	30.8 (5.0)	0.13
DLCO (%)	47.3 (13.6)	37.2 (5.9)	0.13

S5 Table (continued)

Symptoms			
With cough – no (%)	28 (82.4)	10 (83.3)	1.00
With wheezing – no (%)	3 (8.8)	4 (30.8)	0.15
With emphysema no. (%)	30 (88.9)	5 (38.5)	**0.001**
Comorbidities			
Cardiovascular – no. (%)	18 (52.9)	4 (30.8)	0.20
Diabetes – no. (%)	8 (23.5)	1 (7.7)	0.11
Treatments			
On SABA – no. (%)	32 (94.1)	12 (92.3)	1.00
On LABA – no. (%)	32 (94.1)	9 (69.3)	**0.04**
On LAMA – no. (%)	32 (100.0)	10 (90.9)	0.57
On ICS – no. (%)	32 (94.1)	11 (84.6)	0.65
Daily dose of equivalents beclomethasone	864.1 ± 382.3	752.5 ± 298.2	0.45
On oxygen therapy – no. (%)	21 (61.8)	6 (46.2)	0.52

Data are n (%), or means ± SD, or median (25-75 interquartile range), or means ± SEM, for the number of exacerbations

CRP = C reactive protein; FEV_1 = Forced Expiratory Volume in 1 second; FVC = Forced Vital Capacity; DLCO = transfer factor of the lung for carbon monoxide; SABA = short-acting β2-agonists; LABA = long-acting β2-agonists; LAMA = long-lasting muscarinic antagonists; ICS = inhaled corticosteroids.

* Students' t test, or Fisher exact test 2-tailed, or Mann-Whitney U-test

[a] Estimated frequency of exacerbations and hospitalizations, according to the events during the study period
