# Supplementary material for: Proteomic profiling of serum identifies a molecular signature that correlates with clinical outcomes in COPD
Source: PLoS One. 2022 Dec 8;17(12):e0277357. doi: 10.1371/journal.pone.0277357 (PMC9731494; doi:10.1371/journal.pone.0277357)
Supplement: S6 Table — (PDF) [file pone.0277357.s007.pdf]

Supporting information

S6 Table. Main clinical characteristics of COPD patients switching of Clusters between visit 1 and visit 2

Parameter	Values at visit 1	Values at visit 2	p value *
**COPD patients switching from Cluster 1 at visit 1 to Cluster 2 at visit 2 (n = 29)**			
With emphysema - no. (%) [b]	16 (55.2)	13 (44.8)	0.44
DLCO - %	49.4 ± 13.3	54.3 ± 28.5	0.60
With exacerbations in the previous 12 months - no. (%)	20 (69.0)	11 (37.9)	**0.02**
With unscheduled medical visits in the previous 12 months - no. (%)	16 (55.2)	8 (27.6)	**0.04**
With hospitalizations for COPD in the previous 12 months – no. (%)	8 (27.6)	1 (3.4)	**0.02**
On SABA – no. (%)	19 (65.5)	18 (69.2)	1.00
On LABA – no. (%)	22 (75.9)	20 (70)	0.77
On LAMA – no. (%)	19 (65.5)	20 (70)	1.00
On ICS – no. (%)	13 (44.8)	15 (51.7)	0.79
On anti-hypertensive drugs – no. (%)	12 (41.4)	10 (38.5)	0.79
On statins – no. (%)	11 (37.9)	10 (38.5)	1.00
Adherence to treatment – no. (%)	26 (89.7)	26 (89.7)	1.00
**COPD patients switching from Cluster 2 at visit 1 to Cluster 1 at visit 2 (n = 26)**			
With emphysema - no. (%)	5 (19.2)	11 (42.3)	0.08
DLCO - %	53.6 ± 18.0	53.4 ± 10.9	0.99
With exacerbations in the previous 12 months - no. (%)	15 (57.7)	10 (38.5)	0.17
With unscheduled medical visits in the previous 12 months - no. (%)	10 (38.5)	8 (30.8)	0.56
With hospitalizations for COPD in the previous 12 months – no. (%)	1 (4)	6 (23.1)	**0.05**
On SABA – no. (%)	20 (69)	19 (73.1)	1.00
On LABA – no. (%)	18 (62.1)	21 (80.8)	0.52
On LAMA – no. (%)	12 (46.2)	20 (76.9)	**0.04**
On ICS – no. (%)	13 (50)	15 (57.7)	0.74
On anti-hypertensive drugs – no. (%)	7 (24.1)	8 (30.8)	1.00
On statins – no. (%)	2 (6.9)	3 (11.5)	1.00
Adherence to treatments - no. (%)	23 (92.0)	24 (100.0)	1.00

Data are expressed as numbers (%) and as means ± SD

DLCO = transfer factor of the lung for carbon monoxide; SABA = short-acting β2-agonists; LABA = long-acting β2-agonists; LAMA = long-lasting muscarinic antagonists; ICS = inhaled corticosteroids.

* Students' t test or Fisher exact test 2-tailed
