# Supplementary figures and images for: Proteomic profiling of serum identifies a molecular signature that correlates with clinical outcomes in COPD
Source: PLoS One. 2022 Dec 8;17(12):e0277357. doi: 10.1371/journal.pone.0277357 (PMC9731494; doi:10.1371/journal.pone.0277357)

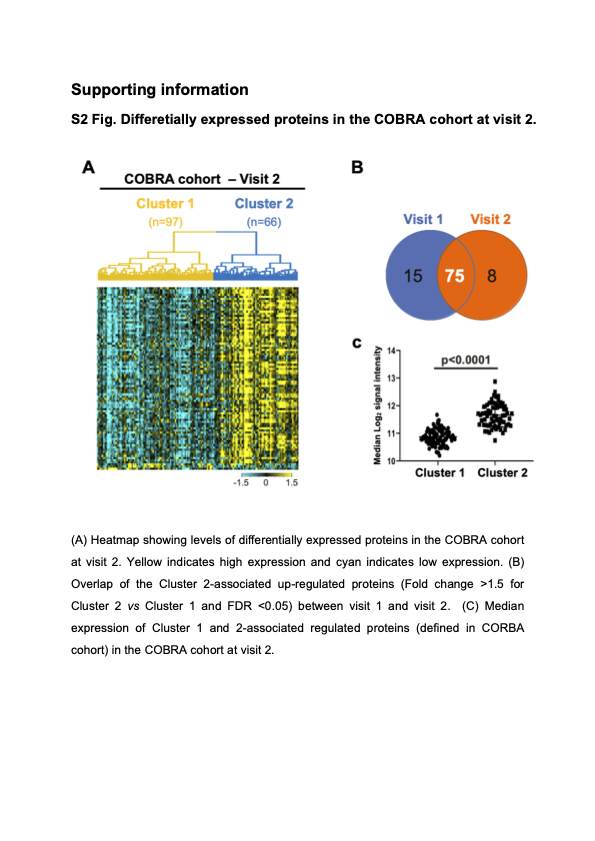

Supplement: S1 Fig — (TIFF) [file pone.0277357.s008.tiff]

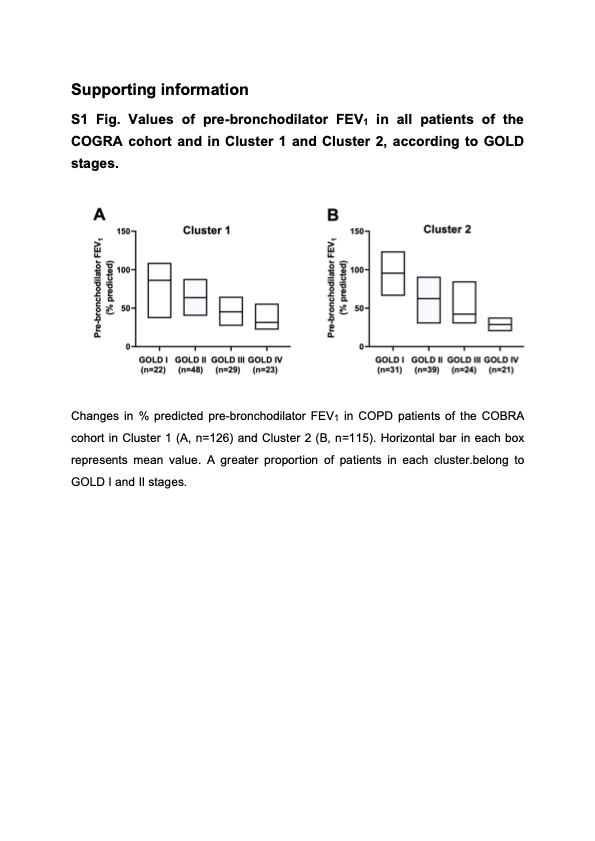

Supplement: S2 Fig — (TIFF) [file pone.0277357.s009.tiff]
